# Supplementary material for: Ragweed Major Allergen Amb a 11 Recombinant Production and Clinical Implications
Source: Biomolecules. 2023 Jan 16;13(1):182. doi: 10.3390/biom13010182 (PMC9855870; doi:10.3390/biom13010182)
Supplement: Supplementary file 1 [file biomolecules-13-00182-s001.zip › biomolecules-2125843-supplementary/QIAGEN_QIAexpressionist_EN.pdf]

Fifth Edition

The QIA*expressionist*™

A handbook for
high-level expression and purification
of 6xHis-tagged proteins

June 2003

QIAGEN

© 2001–2003 QIAGEN, all rights reserved.

QIAGEN Worldwide

QIAGEN Companies

**Australia**	**QIAGEN Pty Ltd**<br>ABN 75 072 382 944	PO Box 25 • Clifton Hill • Victoria 3068<br>Orders 03-9489-3666 • Fax 03-9489-3888 • Technical 1-800-243-066
**Canada**	**QIAGEN Inc.**	2800 Argentia Road • Unit 7 • Mississauga • Ontario • L5N 8L2<br>Orders 800-572-9613 • Fax 800-713-5951 • Technical 800-DNA-PREP (800-362-7737)
**France**	**QIAGEN S.A.**	3 avenue du Canada • LP 809 • 91974 COURTABOEUF CEDEX<br>Orders 01-60-920-920 • Fax 01-60-920-925 • Technical 01-60-920-930
**Germany**	**QIAGEN GmbH**	QIAGEN Strasse 1 • 40724 Hilden<br>Orders 02103-29-12000 • Fax 02103-29-22000 • Technical 02103-29-12400
**Italy**	**QIAGEN S.p.A.**	Via Grosio, 10/10 • 20151 Milano<br>Orders 02-33430411 • Fax 02-33430426 • Technical 02-33430414
**Japan**<br>www.qiagen.co.jp	**QIAGEN K.K.**	Forefront Tower II • 13-1, Kachidoki 3 Chome • Chuo-ku, Tokyo 104-0054<br>Telephone 03-5547-0811 • Fax 03-5547-0818 • Technical 03-5547-0811
**Switzerland**	**QIAGEN AG**	Auf dem Wolf 39 • 4052 Basel<br>Orders 061-319-30-30 • Fax 061-319-30-33 • Technical 061-319-30-31
**UK and Ireland**	**QIAGEN Ltd.**	Boundary Court • Gatwick Road • Crawley • West Sussex, RH10 9AX<br>Orders 01293-422-911 • Fax 01293-422-922 • Technical 01293-422-999
**USA**	**QIAGEN Inc.**	28159 Avenue Stanford • Valencia • CA 91355<br>Orders 800-426-8157 • Fax 800-718-2056 • Technical 800-DNA-PREP (800-362-7737)

www.qiagen.com

QIAGEN Distributors

Please see the last page for contact information for your local QIAGEN distributor.

Contents

Kit Contents 7

Storage Conditions 8

Technical Assistance 9

Product Use Limitations 9

Product Warranty and Satisfaction Guarantee 9

Safety Information 10

Introduction and General Guidelines 11

The QIA*express* System 15

QIA*express* pQE vectors 15

QIA*express* pQE-TriSystem Vector for expression in *E.coli*, mammalian and insect cells 16

Regulation of expression — pREP4 plasmid 16

E. coli host strains 17

The 6xHis tag 18

Ni-NTA technology 18

Ni-NTA chromatographic materials 19

Cloning 21

Choosing a QIA*express* construct 21

Intended use of recombinant proteins and pQE vector choice 22
Protein size 22
Codon usage 23
Internal start sites 23
Inefficient translation 23
Secretion 23

Cloning procedures and vector maps 24

Procedure for direct cloning of PCR fragments using pQE-30 UA 24

Troubleshooting: UA cloning 28

Cloning procedures with pQE vectors using restriction enzymes 29

Preparation of pQE expression constructs 29
Propagation of pQE plasmids and constructs 35
Ligation, transformation, and screening 36

Integration of 6xHis tags in other vectors 37
PCR mutagenesis to insert 6xHis tag 38
Transformation of *E. coli* 39
Analysis of transformants 41

Cloning protocols

Protocol 1. Ligation with pQE-30 UA 26
Protocol 2. Preparation of competent *E. coli* 39
Protocol 3. Transformation of competent M15 cells 40
Protocol 4. Colony-blot procedure 41
Protocol 5. Rapid screening of small expression cultures 45

Troubleshooting: cloning 47

Expression in *E. coli* 48

Basic principles 49

Culture media 49
Maintenance of the expression plasmid 50
Small-scale expression cultures 50
Time-course analysis of protein expression 50

Specific considerations 51

Low expression levels 51
Toxic gene products 51
Hydrophobic regions 52
Unstable proteins 52
High expression levels, insoluble proteins, and inclusion bodies 53

Other Expression Systems 53

Baculovirus 54
Mammalian cells 57
Yeast 57

Expression procedures 59

Expression protocols

Protocol 6. Determination of target protein solubility 59
Protocol 7. Growth of standard *E. coli* expression cultures (100 ml) 61
Protocol 8. *E. coli* culture growth for preparative purification (1 liter) 61

Troubleshooting: expression 62

Purification 63

Basic principles 65

Culture size 65
Reducing nonspecific binding 66
Protein solubility and intracellular location 66
Purification under native or denaturing conditions 67
Batch or column purification 69
Protein binding 70
Wash 70
Protein elution 71
Removal of the 6xHis tag 71

Specific considerations 72

Contaminating proteins 72
Limitations 73

Purification of 6xHis-tagged proteins produced in other expression systems 76

Purification of 6xHis-tagged proteins expressed in mammalian cells 76
Purification of 6xHis-tagged proteins expressed in insect cells 77

Purification procedures 79

Purification protocols

Protocol 9. Preparation of cleared *E. coli* lysates under native conditions 79
Protocol 10. Preparation of cleared *E. coli* lysates under denaturing conditions 80
Protocol 11. Preparation of 6xHis-tagged periplasmic proteins from *E. coli* 81
Protocol 12. Batch purification of 6xHis-tagged proteins from *E. coli* under native conditions 82
Protocol 13. FPLC purification of 6xHis-tagged proteins from *E. coli* using Ni-NTA Superflow under native conditions 83
Protocol 14. Protein minipreps of 6xHis-tagged proteins from *E. coli* under native conditions 85
Protocol 15. Purification of 6xHis-tagged proteins from transfected mammalian cells under native conditions 86
Protocol 16. Purification of 6xHis-tagged proteins from baculovirus-infected insect cells under native conditions 88
Protocol 17. Batch purification of 6xHis-tagged proteins from *E. coli* under denaturing conditions 90
Protocol 18. FPLC purification of 6xHis-tagged proteins using Ni-NTA Superflow under denaturing conditions 91
Protocol 19. 6xHis-tagged protein minipreps under denaturing conditions 92
Protocol 20. Factor Xa Protease treatment of fusion proteins containing a Factor Xa Protease recognition sequence 93

Troubleshooting: purification from *E. coli* 99
Troubleshooting: purification from mammalian cells 101
Troubleshooting: purification from insect cells 103
Troubleshooting: Factor Xa Protease cleavage 104

Protein Refolding Recommendations 106

Appendix 108

Ni-NTA Matrices 108
Specifications 108
Handling 109
Reuse of Ni-NTA Matrices 109
Preparation of guanidine-containing samples for SDS-PAGE 110
Media, solutions, and reagents 111
Bacterial media and solutions 111
Buffers for preparing competent *E. coli* 111
SDS-PAGE sample buffers 111
Solutions for colony-blot procedure 111
Alkaline phosphatase (AP) staining solutions 112
Horseradish peroxidase staining solutions 112
Buffers for purification under denaturing conditions 113
Buffers for purification under native conditions 114
Buffers for purification from mammalian cells using Ni-NTA Magnetic Agarose Beads under native conditions 114
Buffer for Factor Xa Protease digestion and removal of Factor Xa Protease with Xa Removal Resin 115
QIA*express* pQE vectors: multiple cloning sites 116
Restriction map of pREP4 118
Sequencing primers for pQE vectors 118

References 119

Ordering Information 122

QIAGEN Companies and Distributors 127

Kit Contents

QIA*express* Kit<br>Catalog No.	Type IV<br>32149	Type ATG<br>32169
pQE vectors	5 µg each<br>pQE-30,<br>pQE-31, pQE-32	5 µg each<br>pQE-60,<br>pQE-70
pREP4	1 µg	1 µg
Control expression plasmid (pQE-40)	1 µg	1 µg
E. coli host strain M15[pREP4]	1 vial	1 vial
E. coli host strain SG13009[pREP4]	1 vial	1 vial
Ni-NTA Agarose	10 ml	10 ml
Disposable columns, 5 ml bed-volume	5	5
Disposable columns, 1 ml bed-volume	5	5
Frits for 5 ml disposable columns	10	10
Frits for 1 ml disposable columns	10	10
Sodium phosphate stock solution (0.5 M NaH_2PO_4, 50 mM Tris)	100 ml	100 ml
Imidazole stock solution (1 M)	50 ml	50 ml
Urea	100 g	100 g
Guanidine hydrochloride	40 g	40 g
IPTG (for 1 ml 1 M stock solution)	238 mg	238 mg
*The QIA*expressionist™	1	1

Kit Contents

Kit	N-Terminus pQE Vector Set	C-Terminus pQE Vector Set	*cis*-Repressed pQE Vector Set	pQE-100 DoubleTag Vector
Catalog No.	32915	32903	32923	33003
pQE vectors	pQE-9, -30, -31, -32, and 40	pQE-16, -60, and 70	pQE-80L, -81L and 82L	pQE-100
*The QIA*expressionist	1	1	1	1

Product	Contents	Cat. No.
Polypropylene Columns (1 ml)	50/pack, 1 ml capacity	34924
Polypropylene Columns (5 ml)	50/pack, 5 ml capacity	34964
pQE Sequencing-Primer Set	0.1 A260 unit each of Primer – Promotor region, Primer – Type III/IV, and Primer - Reverse Sequencing (3.0, 2.8, and 3.1 µg, respectively; lyophilized)	34051
Host Strains	One stab culture each of E. coli M15[pREP4] and SG13009[pREP4]	34210
Factor Xa Protease	400 units Factor Xa Protease (2U/µl)	33223
Xa Removal Resin	2 x 2.5 ml Xa Removal Resin, 3 x 1.9 ml 1 M Tris·Cl pH 8.0	33213
pQE-30 Xa Vector	25 µg pQE-30 Xa Vector DNA	33203
pQE-TriSystem Vector	25 µg pQE-TriSystem Vector DNA	33903
QIA*express* UA Cloning Kit (20)	For 20 reactions: 2 x Ligation Master Mix (100 µl), pQE-30 UA Vector DNA (1 µg; 50 ng/µl), distilled water (1.7 ml)	32179

Storage Conditions

Ni-NTA matrices, *E. coli* host strains, sodium phosphate stock solution, and imidazole stock solution should be stored at 2–8°C. The *E. coli* host strain can be stored under these conditions for up to 3 months without significant loss of viability – we recommend establishing cultures and storing your own stabs or glycerol stocks as soon as possible after receipt of your kit. All other kit components can be stored under these conditions for up to 1 year without any reduction in performance. Ni-NTA matrices should not be frozen.

QIA*express* pQE vectors are supplied lyophilized with sucrose and bromophenol blue for visualization, and should be resuspended in a convenient volume of TE (e.g., 10 µl) and stored at –20°C. Sucrose and bromophenol blue do not interfere with restriction digestions or bacterial transformation.

QIA*express* UA Cloning Kit components should be stored at –20°C. Kit components can be stored under these conditions for up to 6 months without any reduction in performance.

Factor Xa Protease should be stored at –20°C. Factor Xa Protease can be stored under these conditions for up to 6 months without any reduction in activity.

Xa Removal Resin should be stored at 2–8°C. The resin can be stored under these conditions for up to 6 months without any reduction in performance.
Xa Removal resin should not be frozen.

Technical Assistance

At QIAGEN we pride ourselves on the quality and availability of our technical support. Our Technical Service Departments are staffed by experienced scientists with extensive practical and theoretical expertise in molecular biology and the use of QIAGEN® products. They are always available to discuss any general or specific questions you may have. If you have any questions or experience any problems regarding QIA*express*, or QIAGEN products in general, please do not hesitate to contact us.

QIAGEN customers are also a major source of information regarding advanced or specialized uses of our products. This information is helpful to other scientists as well as to the researchers at QIAGEN. We therefore encourage you to contact us if you have any suggestions about product performance or new applications and techniques.

For technical assistance and more information please call one of the QIAGEN Technical Service Departments or local distributors (see inside front cover).

Product Use Limitations

QIA*express* products are developed, designed, and sold for research purposes only. They are not to be used for human diagnostic or drug purposes or to be administered to humans unless expressly cleared for that purpose by the Food and Drug Administration in the USA or the appropriate regulatory authorities in the country of use. All due care and attention should be exercised in the handling of many of the materials described in this text.

Product Warranty and Satisfaction Guarantee

QIAGEN guarantees the performance of all products in the manner described in our product literature. The purchaser must determine the suitability of the product for its particular use. Should any product fail to perform satisfactorily due to any reason other than misuse, QIAGEN will replace it free of charge or refund the purchase price. We reserve the right to change, alter, or modify any product to enhance its performance and design. If a QIAGEN product does not meet your expectations, simply call your local Technical Service Department or distributor. We will credit your account or exchange the product — as you wish.

A copy of QIAGEN terms and conditions can be obtained on request, and is also provided on the back of our invoices. If you have questions about product specifications or performance, please call QIAGEN Technical Services or your local distributor (see inside front cover).

Safety Information

When working with chemicals, always wear a suitable lab coat, disposable gloves, and protective goggles. For more information, please consult the appropriate material safety data sheets (MSDSs). These are available online in convenient and compact PDF format at **www.qiagen.com/ts/msds.asp** where you can find, view, and print the MSDS for each QIAGEN kit and kit component.

CAUTION: DO NOT add bleach or acidic solutions directly to the sample-preparation waste.

The QIA*express* Type IV and ATG kits contain guanidine hydrochloride, which can form highly reactive compounds when combined with bleach.

In case liquid containing these buffers is spilt, clean with suitable laboratory detergent and water. If the spilt liquid contains potentially infectious agents, clean the affected area first with laboratory detergent and water, and then with 1% (v/v) sodium hypochlorite.

QIA*express* Type IV Kit and Type ATG Kit

5x phosphate buffer stock solution

Contains sodium hydroxide: Irritant. Risk and safety phrases*: R36/38. S13-26-36-46.

Guanidine hydrochloride

Contains guanidine hydrochloride. Harmful, and irritant. Risk and safety phrases*: R22-36/38. S22-26-36/37/39.

Imidazole solution

Contains imidazole. Irritant. Risk and safety phrases*: R36/37/38. S23-26-36/37/39-45.

Ni-NTA Agarose

Contains nickel-nitrilotriacetic acid. Harmful, sensitizer, and flammable. Risk and safety phrases:* R10-22-40-42/43. S13-26-36-46.

24-hour emergency information

Emergency medical information in English, French, and German can be obtained 24 hours a day from: Poison Information Center Mainz, Germany

Tel: +49-6131-19240

* *R10: Flammable. R22: Harmful if swallowed. R36/38: Irritating to eyes and skin. R36/37/38: Irritating to eyes, respiratory system and skin. R40: Possible risks of irreversible effects. R42/43: May cause sensitization by inhalation and skin contact. S13: Keep away from food, drink and animal feedingstuffs. S22: Do not breathe dust. S23: Do not breathe spray. S26: In case of contact with eyes, rinse immediately with plenty of water and seek medical advice. S36: Wear suitable protective clothing. S36/37/39: Wear suitable protective clothing, gloves and eye/face protection. S45: In case of accident or if you feel unwell, seek medical advice immediately (show the label where possible). S46: If swallowed, seek medical advice immediately and show the container or label.*

Introduction and General Guidelines

The expression and purification of recombinant proteins facilitates production and detailed characterization of virtually any protein. Although a wide variety of heterologous expression systems have been developed and are currently used to produce recombinant proteins, the purification of the proteins obtained can still be problematic. Classical purification procedures can be employed, but in most cases recombinant DNA techniques permit the construction of fusion proteins in which specific affinity tags are added to the protein sequence of interest; the use of these affinity tags simplifies the purification of the recombinant fusion proteins by employing affinity chromatography methods.

The QIA*express*® System is based on the remarkable selectivity and affinity of QIAGEN's exclusive, patented nickel-nitrilotriacetic acid (Ni-NTA) metal-affinity chromatography matrices for biomolecules which have been tagged with 6 consecutive histidine residues (6xHis tag, Figure 1). The unique features of the QIA*express* System provide a number of significant advantages (Table 1) that are not available with other affinity-tag and chromatography methods.

QIA*express* expression products comprise vectors that can be used for expression of 6xHis-tagged recombinant proteins in bacterial, baculovirus, and mammalian expression systems.

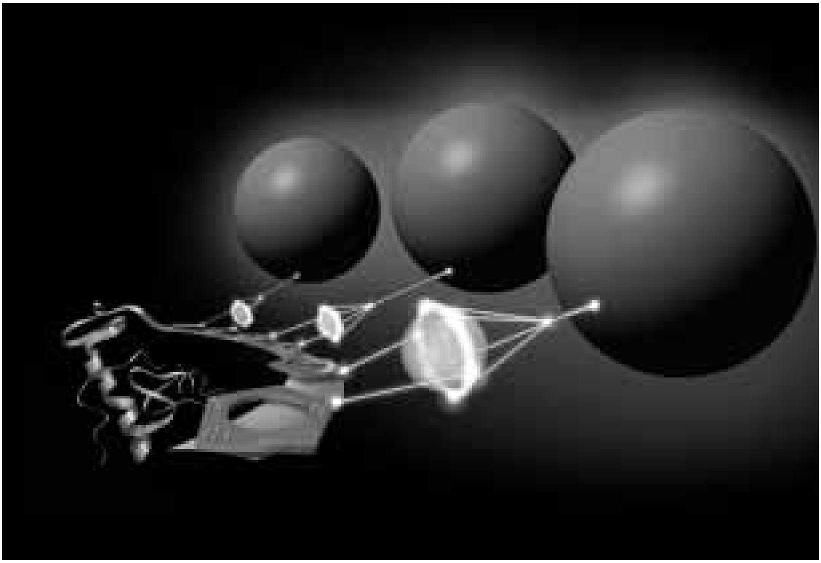

Figure 1. *Interaction between Ni-NTA and a 6xHis-tagged protein*

QIA*express* provides a complete system for the expression, purification, detection, and assay of 6xHis-tagged proteins. Protein expression with the QIA*express* System begins with constructing expression clones, followed by the expression of 6xHis-tagged proteins and purification on Ni-NTA matrices (Figure 2). For detection and assay of 6xHis-tagged proteins, additional products are available (see ordering information on page 122), and a detailed handbook describing the use of these products is available. For a free copy, call one of the QIAGEN Technical Service Departments or local distributors (see inside front cover).

Table 1. Features and benefits of the QIA*express* system

Features	Benefits
• The interaction of the 6xHis tag with Ni-NTA matrices is conformation independent.	• One-step purification can be carried out under native or denaturing conditions.
• Mild elution conditions can be used.	• Binding, washing, and elution are highly reproducible, and have no effect on protein structure.
	• Pure protein products are ready for direct use in downstream applications.
• The 6xHis tag is much smaller than other commonly used tags.	• 6xHis tags can be used in any expression system.
	• Tag does not interfere with the structure and function of the recombinant protein.
• The 6xHis tag is uncharged at physiological pH.	• The 6xHis tag does not interfere with secretion.
• The 6xHis tag is poorly immunogenic.	• The recombinant protein can be used without prior removal of the tag as an antigen to generate antibodies against the protein of interest.
• Using Factor Xa Protease, 6xHis tag can be easily and efficiently removed	• The detagged protein can be used for crystallographical or NMR studies where removal of the 6xHis tag may be preferred.
• Some QIA*express* vectors feature a 6xHis-dihydrofolate reductase tag (6xHis-DHFR tag).	• Small peptides fused to the 6xHis DHFR tag are stabilized while being expressed.
	• The 6xHis-DHFR tag is not highly immunogenic in mouse and rat, so that peptides fused to the tag can be used directly for immunizations or epitope mapping.

The following are general guidelines for using the QIA*express* System and *The QIA*expressionist.

- *The QIA*expressionist is divided into three main sections that describe the steps leading to a pure, 6xHis-tagged protein: cloning (page 21), expression (page 48), and purification (page 63). Preceding these sections and immediately following the introduction is a chapter detailing the QIA*express* System (page 15).
- If you have not worked with bacterial expression vectors before, you should first review the introduction, and the cloning section that addresses the cloning steps which include the design of the construct, preparation of insert and vector DNA, ligation, transformation, and identification of clones that express the desired protein. Specific cloning protocols (restriction digestion, ligation etc.) are not presented here, but can be found in standard laboratory manuals (Ausubel et al. 1995; Sambrook et al. 1989). Once you have a clear understanding of the strategies and the methods necessary to produce the desired 6xHis-tagged protein, proceed with the expression (page 48) and purification (page 63) sections.
- If you already have a bacterial clone that expresses a 6xHis-tagged protein, make sure that you understand the problems that can arise if the clone is not properly maintained, cultured, and stored. Review the introduction to the QIA*express* System (page 15) and then proceed to the expression (page 48) and purification (page 63) sections.
- If you are already familiar with expression vectors, but have not worked with 6xHis-tagged proteins and Ni-NTA resins, you should review the chapters detailing the QIA*express* System (page 15), protein expression (page 48), and purification of 6xHis-tagged proteins (page 63).
- If you are producing 6xHis-tagged proteins in yeast, baculovirus, or mammalian expression systems and have not worked with Ni-NTA resins, you need to be aware of some of the specific circumstances that can affect the success of your purification procedures when using Ni-NTA matrices. Guidelines and references for the purification of 6xHis-tagged proteins produced in yeast are included in the section "Yeast", page 57. For the purification of 6xHis-tagged proteins produced in baculovirus and mammalian expression systems specific protocols are included in this handbook (see pages 88 and 86 respectively). Be sure to review the purification section (page 63) and specifically "Purification of 6xHis-tagged proteins produced in other expression systems" (page 76).
- The intracellular solubility of the 6xHis-tagged protein expressed by your clone determines the choice of lysis and purification protocols that work under native conditions (for soluble proteins) or denaturing conditions (for insoluble proteins). The choice of these protocols is also influenced by the scale, i.e. the amount of the recombinant protein that is to be purified.

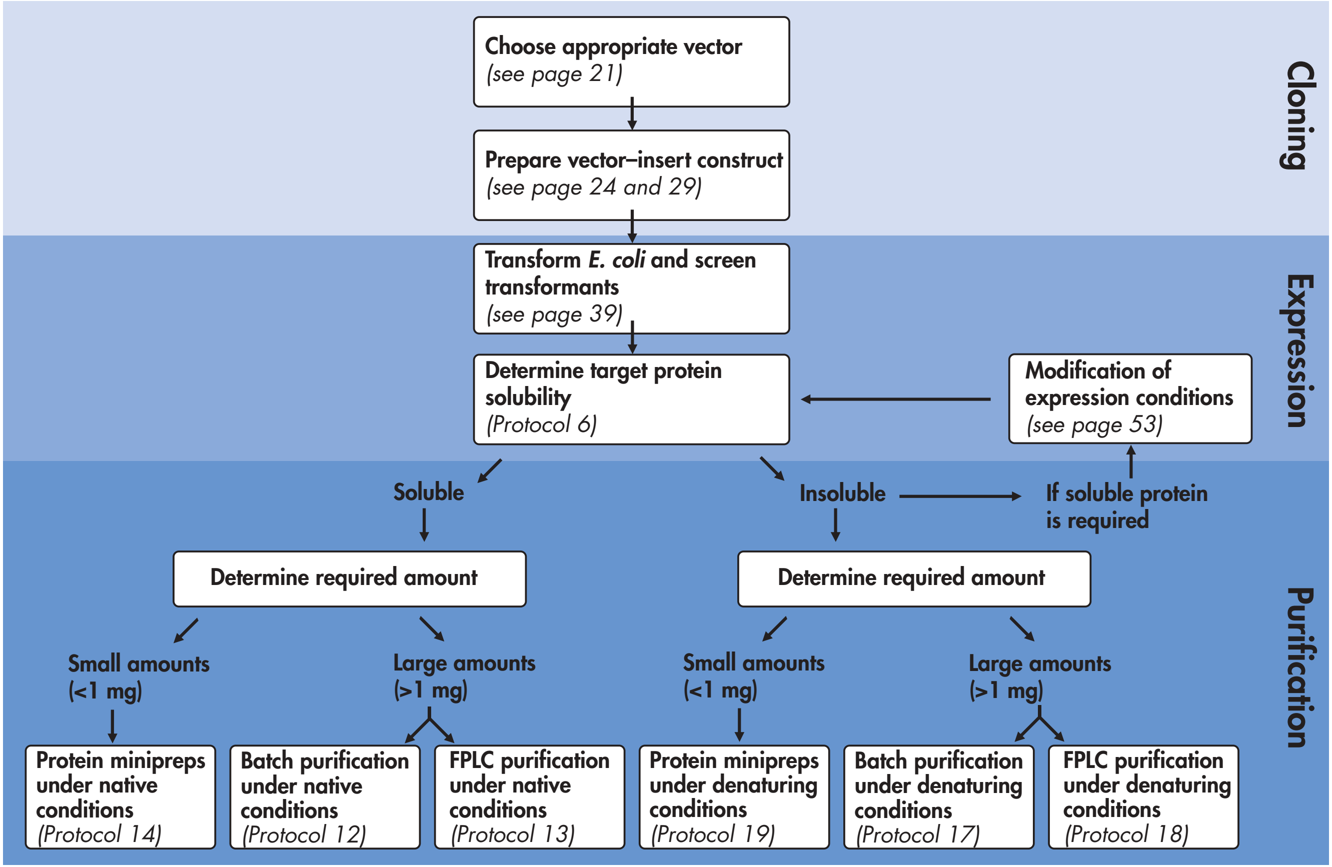

Figure 2. *Strategy for the expression and purification of 6xHis-tagged proteins using the QIAexpress System.*

The QIA*express* System

The QIA*express* System provides materials for expression, purification, detection, and assay of 6xHis-tagged proteins. *The QIA*expressionist covers expression and purification products (pQE vectors, host strains, and Ni-NTA chromatographic matrices) which allow the fast and efficient production and purification of heterologously expressed 6xHis-tagged proteins.

QIA*express* pQE vectors

High-level expression of 6xHis-tagged proteins in *E. coli* using pQE vectors is based on the T5 promoter transcription–translation system. pQE plasmids belong to the pDS family of plasmids (Bujard et al. 1987) and were derived from plasmids pDS56/RBSII and pDS781/RBSII-DHFRS (Stüber et al. 1990). These low-copy plasmids (Figure 3) have the following features:

- Optimized promoter–operator element consisting of phage T5 promoter (recognized by the *E. coli* RNA polymerase) and two *lac* operator sequences which increase *lac* repressor binding and ensure efficient repression of the powerful T5 promoter
- Synthetic ribosomal binding site, RBSII, for high translation rates
- 6xHis-tag coding sequence either 5' or 3' to the cloning region
- Multiple cloning site and translational stop codons in all reading frames for convenient preparation of expression constructs
- Two strong transcriptional terminators: t_0 from phage lambda (Schwarz et al. 1987), and T1 from the *rrnB* operon of *E. coli*, to prevent read-through transcription and ensure stability of the expression construct
- β-lactamase gene (*bla*) conferring resistance to ampicillin (Sutcliffe 1979) at 100 µg/ml. The chloramphenicol acetyl transferase gene (CAT) present between t_0 and T1 has no promoter and is not normally expressed.* Depending on the bacterial strain and insert, low CAT activities may be detectable.
- ColE1 origin of replication (Sutcliffe 1979)*

Restriction maps and sequences for the cloning regions of QIA*express* vectors are presented in the appendix on page 117. The entire sequence information is available at **www.qiagen.com/literature/vectors.asp**

* *pQE-TriSytem does not contain a CAT cassette and has a pUC origin of replication.*

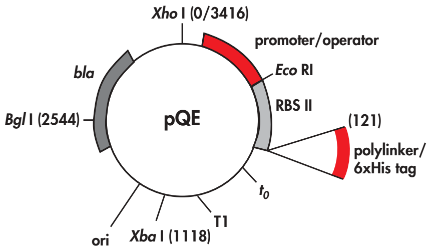

Figure 3. *pQE vectors.*

QIA*express* pQE-TriSystem Vector for expression in *E. coli*, mammalian and insect cells

High-level expression of His-tagged proteins from a single vector is possible in three different expression systems due to the presence of the T5 promoter/*lac* operator transcription–translation system for expression in *E. coli*; the p10 promoter for baculovirus-based expression in insect cells; and the CAG (CMV/actin/globin) promoter for expression in mammalian cells. The pQE-TriSystem vector has the following features:

- An optimized promoter-operator element consisting of the coliphage T5 promoter (recognized by the *E. coli* RNA polymerase) and the *lac* operator sequence which binds *lac* repressor and ensures efficient repression of the powerful T5 promoter in *E. coli*.
- Synthetic bacterial ribosomal binding site for high translation rates.
- Strong, constitutive CAG promoter that mediates transient mammalian expression. It consists of the chicken β-actin promoter with the CMV immediate-early enhancer upstream, and a splicing acceptor site of the β-globin gene downstream.
- Downstream rabbit-globin–terminator sequence containing the signals for polyadenylation of the mRNA transcript.
- Kozak consensus sequence facilitating efficient translation initiation in mammalian systems.
- Late-very late p10 promoter for controlled recombinant protein expression in insect cells.
- Two segments derived from the open reading frames 603 and 1629 of *Autographa californica* nuclear polyhedrosis virus (AcNPV) genomic DNA that allow generation of recombinant baculovirus by homologous recombination.
- 8xHis-tag coding sequence 3' of the cloning region (C-terminal His-tag)
- A multiple cloning site and translational stop codons in all reading frames for convenient preparation of expression constructs.
- Two strong transcription terminators — T7 from *E. coli* bacteriophage T7 and rabbit globin terminator region — to prevent read-through transcription and ensure stability of the expression construct.
- β-lactamase gene (*bla*) which confers resistance to ampicillin at 100 μg/ml in *E. coli*.
- pUC origin of replication, compatible with pREP4 in *E. coli*.

Regulation of expression — pREP4 plasmid

The extremely high transcription rate initiated at the T5 promoter can only be efficiently regulated and repressed by the presence of high levels of the *lac* repressor protein. *E. coli* host strains used in the QIA*express* System use a *lac* repressor gene in *trans* or *cis* to the gene to be expressed. In the *trans* system, the host strains contain the low-copy plasmid

pREP4 (see appendix, page 118) which confers kanamycin resistance and constitutively expresses the *lac* repressor protein encoded by the *lac* I gene (Farabaugh 1978). The pREP4 plasmid is derived from pACYC and contains the p15A replicon. Multiple copies of pREP4 are present in the host cells that ensure the production of high levels of the *lac* repressor protein which binds to the operator sequences and tightly regulates recombinant protein expression. The pREP4 plasmid is compatible with all plasmids carrying the ColE1 origin of replication, and is maintained in *E. coli* in the presence of kanamycin at a concentration of 25 µg/ml. The *cis*-repressed vectors pQE-80L, -81L, and -82L contain the *lacI*^{q} gene and do not require the presence of pREP4.

Expression of recombinant proteins encoded by pQE vectors is rapidly induced by the addition of isopropyl-β-D-thiogalactoside (IPTG) which binds to the *lac* repressor protein and inactivates it. Once the lac repressor is inactivated, the host cell's RNA polymerase can transcribe the sequences downstream from the promoter. The transcripts produced are then translated into the recombinant protein. The special "double operator" system in the pQE expression vectors, in combination with the high levels of the *lac* repressor protein generated by pREP4 or the *lacI*^{q} gene on pQE-80L, pQE-81L, or pQE-82L, ensure tight control at the transcriptional level. The pREP4 plasmid is already present in the QIA*express* *E. coli* strains M15[pREP4] and SG13009[pREP4]. Using pQE-80L, pQE-81L, or pQE-82L with the *cis-lacI*^{q} gene, expression rates are comparable with those obtained using pQE-30, pQE-31, or pQE-32 vectors in combination with pREP4.

E. coli host strains

Any *E. coli* host strain containing both the expression (pQE) and the repressor (pREP4) plasmids can be used for the production of recombinant proteins. The QIA*express* System uses *E. coli* strain M15[pREP4] which permits high-level expression and is easy to handle. Strain SG13009[pREP4] (Gottesman et al. 1981) is also supplied and may be useful for the production of proteins that are poorly expressed in M15[pREP4]. Both the M15 and SG13009 strains derived from *E. coli* K12 and have the phenotype Nal^S, Str^S, Rif^S, Thi$^-$, Lac$^-$, Ara$^+$, Gal$^+$, Mtl$^-$, F$^-$, RecA$^+$, Uvr$^+$, Lon$^+$.

E. coli strains that harbor the *lacI*^{q} mutation, such as XL1 Blue, JM109 and TG1, produce enough *lac* repressor to efficiently block transcription, and are ideal for storing and propagating pQE plasmids. These strains can also be used as expression hosts for expressing nontoxic proteins, but they may be less efficient than the M15[pREP4] strain, and expression is regulated less tightly than in strains harboring the pREP4 plasmid. If the expressed protein is toxic to the cell, "leaky" expression before induction may result in poor culture growth or in the selection of deletion mutants which grow faster than bacteria containing the correct plasmid. Note that *E. coli* strains M15 and SG13009 do not harbor a chromosomal copy of the *lacI*^{q} mutation, so pREP4 must be maintained by selection for kanamycin resistance. The *cis*-repressed pQE-80L series of vectors can be easily used with any *E. coli* host strain and kanamycin selection is not necessary.

The 6xHis tag

The 6xHis affinity tag facilitates binding to Ni-NTA. Using pQE vectors it can be placed at the C- or N-terminus of the protein of interest. It is poorly immunogenic, and at pH 8.0 the tag is small, uncharged, and therefore does not generally affect secretion, compartmentalization, or folding of the fusion protein within the cell. In most cases, the 6xHis tag does not interfere with the structure or function of the purified protein as demonstrated for a wide variety of proteins, including enzymes, transcription factors, and vaccines. A further advantage of the 6xHis tag is that it allows the immobilization of the protein on metal-chelating surfaces such as Ni-NTA HisSorb™ Strips or Plates and therefore simplifies many types of protein interaction studies. In addition, Anti·His Antibodies can be used for detection.

Ni-NTA technology

Immobilized-metal affinity chromatography (IMAC) was first used to purify proteins in 1975 (Porath et al. 1975) using the chelating ligand iminodiacetic acid (IDA, Figure 4). IDA was charged with metal ions such as Zn^{2+}, Cu^{2+}, or Ni^{2+}, and then used to purify a variety of different proteins and peptides (Sulkowski 1985). IDA has only 3 metal-chelating sites and cannot tightly bind metal ions. Weak binding leads to ion leaching upon loading with strongly chelating proteins and peptides or during wash steps. This results in low yields, impure products, and metal-ion contamination of isolated proteins.

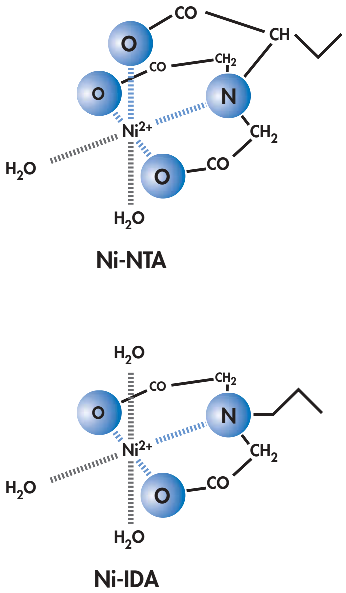

Figure 4. *Comparison of the interactions of different metal chelate matrices with nickel ions.*

Nitrilotriacetic acid (NTA), exclusively available from QIAGEN, is a tetradentate chelating adsorbent developed at Hoffmann-La Roche that overcomes these problems. NTA (Figure 4) occupies four of the six ligand binding sites in the coordination sphere of the nickel ion, leaving two sites free to interact with the 6xHis tag (Figure 5). NTA binds metal ions far more stably than other available chelating resins (Hochuli 1989) and retains the ions under a wide variety of conditions, especially under stringent wash conditions. The unique, patented NTA matrices can therefore bind 6xHis-tagged proteins more tightly than IDA matrices, allowing the purification of proteins from less than 1% of the total protein preparation to more than 95% homogeneity in just one step (Janknecht et al. 1991).

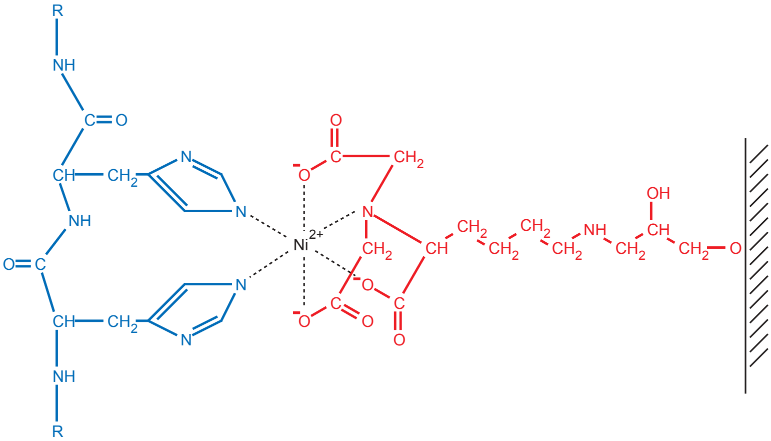

Figure 5. *Interaction between neighboring residues in the 6xHis tag and Ni-NTA matrix.*

Additional information on the interaction of 6xHis-tagged proteins with Ni-NTA matrices can be found in the section “Purification” beginning on page 63.

Ni-NTA chromatographic materials

QIAGEN supplies the following Ni-NTA matrices for the purification of 6xHis-tagged proteins:

Ni-NTA Agarose

Ni-NTA Agarose is composed of Ni-NTA coupled to Sepharose® CL-6B and offers high binding capacity and minimal nonspecific binding. This material has excellent handling properties for batch, column, and low-pressure FPLC®. The high surface concentration of the NTA ligand is sufficient for the binding of approximately 5–10 mg of 6xHis-tagged protein per milliliter of resin. Ni-NTA Agarose is very stable and easy to handle.

Ni-NTA Superflow

Ni-NTA Superflow is comprised of Ni-NTA coupled to Superflow resin. It combines superior mechanical stability with outstanding flow characteristics and high dynamic binding capacity. The capacity for 6xHis-tagged proteins is 5–10 mg/ml. This resin allows one-step purification of 6xHis-tagged proteins using high flow rates and pressures for efficient production-scale and FPLC applications.

Ni-NTA Superflow Columns

Ni-NTA Superflow is available in convenient, pre-packed columns for purification of 6xHis-tagged proteins by gravity-flow or automated purification using the BioRobot 3000 workstation. The automated procedure processes cleared cell lysates and enables purification of up to 24 samples in parallel under denaturing or native conditions. The 1.5 ml resin in each column enable purification of up to 15 mg 6xHis-tagged protein per column.

Ni-NTA Spin Kit and Columns

Ni-NTA silica combines Ni-NTA with a macroporous, silica support material optimized to suppress nonspecific hydrophobic interactions. Ni-NTA Spin Kit and Columns provide Ni-NTA silica in a convenient microspin format for easy preparation of multiple samples in parallel. Each spin column can purify up to 150 µg 6xHis-tagged protein from cellular lysates in 15 minutes using a microcentrifuge.

Ni-NTA Magnetic Agarose Beads

Ni-NTA Magnetic Agarose Beads combine all of the benefits of Ni-NTA with the convenience and speed of magnetic-bead technology. The beads provide rapid, micro-scale high-throughput protein purification as well as a wide range of magnetocapture assays using 6xHis-tagged proteins. They are also ideal for protein purification from samples that have low concentrations of 6xHis-tagged protein, such as low-expressing systems such as mammalian cells — see Protocol 15 (page 86). Ni-NTA Magnetic Agarose Beads are supplied with a comprehensive handbook containing background information and further protocols — contact QIAGEN for further details.

Automated protein purification

Fully automated, walk-away protocols are available to purify up to 15 µg of protein or to perform magnetocapture assays in 96-well format using Ni-NTA Magnetic Agarose Beads on QIAGEN BioRobot™ Systems. To purify larger amounts of 6xHis-tagged protein, the filtration and affinity-chromatography–based Ni-NTA Superflow 96 BioRobot Kit has been developed. Using an adapted protocol, up to 2 mg protein can be purified per well (Schäfer et al. 2002a). Ni-NTA Superflow Columns can be used to purify up to 15 mg 6xHis-tagged protein from cleared lysates in an automated procedure. For more information, call one of the QIAGEN Technical Service Departments or local distributors (see inside front cover).

Cloning

Choosing a QIA*express* construct

Recombinant QIA*express* constructs based on the pQE vectors can be produced by placing the 6xHis tag at the N-terminus of the protein of interest, at the C-terminus, at the C-terminus with the protein beginning with its natural ATG start codon (pQE-60, pQE-70), at the N- or C-terminus combined with the sequence for mouse DHFR, or at the N-terminus combined with the Tag·100 epitope at the C-terminus (Figure 6).

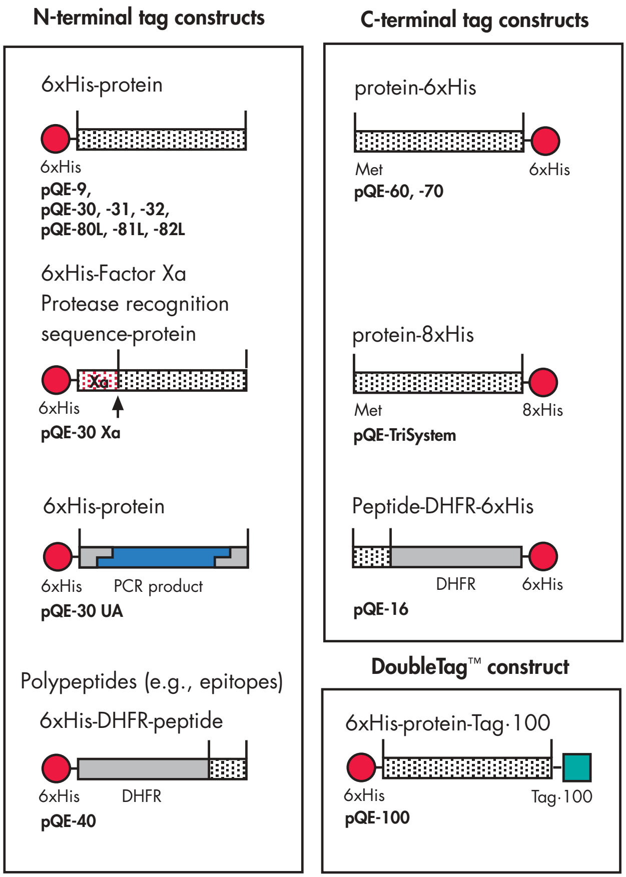

Figure 6. *QIAexpress constructs. The pQE-30 and pQE-80L vector series differ only by the lacI^q gene in the pQE-80L vectors. For vector maps, see pages 32–35.* ***Xa****: Factor Xa Protease recognition sequence. ↥ Factor Xa Protease cleavage site.*

Intended use of recombinant proteins and pQE vector choice

The intended use of the recombinant protein to be purified will determine the choice of the pQE vector and the design of the fusion protein. Constructs which place the 6xHis tag at the N-terminus of the protein, are the most commonly used and are generally the easiest to prepare. Only the 5' end of the open reading frame must be ligated in frame. Since the pQE series of vectors provide termination codons in all three reading frames, the entire coding sequence at the 3' end of the insert does not need to be determined accurately. When using C-terminal tags, the insert must be cloned in frame with both the ATG start codon and the 3' 6xHis coding sequence. When the 6xHis tag is placed at the C-terminus, only full-length proteins are purified. An undetected frame-shift or translational stop codon would lead to premature termination and therefore result in peptides lacking the 6xHis tag. In pQE-60 and pQE-70 constructs only the ribosome binding consensus sequence (Shine-Dalgarno sequence) is provided by the expression vector. These vectors allow the translation of the coding fragment to initiate at the original start codon without fusing additional amino acids to the N-terminus. The optimized translation initiation region in pQE expression vectors enables the expression of proteins in N-terminal tag constructs 2–4 times more efficiently than proteins with a C-terminal affinity tag.

For many applications, the number of amino acids added to the protein along with the 6xHis tag should be kept to a minimum. This can be achieved with pQE-60 or pQE-70, or by cloning the insert at the 5' end of the pQE-30, pQE-31, pQE-32, pQE-80L, pQE-81L, or pQE-82L multiple cloning site (MCS). If the number of additional amino acids is not important, the most convenient restriction sites in the MCS may be used, but many of the advantages conferred by the small size of the 6xHis tag will be lost. If it is desirable to detect the protein or peptide being expressed by using the RGS·His™ Antibody (QIA*express* Detection System), the RGS·His epitope (RGSHHHH) must be present. Vectors that encode the RGS·His epitope include pQE-9, pQE-30–32, pQE-80–82L, and pQE-40. Penta·His™ and Tetra·His™ Antibodies can detect any 6xHis-tagged protein expressed with all pQE vectors. For proteins intended for use in assay systems, we recommend using the pQE-100 DoubleTag Vector which places a 6xHis tag at the N-terminus and a Tag·100 epitope at the C-terminus of the protein — see page 35 for more details.

Protein size

Very small proteins and peptides are sometimes difficult to express stably in *E. coli* because they cannot fold correctly and are often subject to proteolytic degradation. These proteins can be stabilized by expressing them fused to a large protein such as the mouse DHFR protein encoded in a pQE-16 or pQE-40 construct. The DHFR protein is poorly immunogenic in mice and rats and protects the attached peptides from proteolysis after immunization. DHFR enhances the general antigenicity of the peptides to which it is attached by allowing them to fold properly.

Very long recombinant proteins may be subject to premature termination (see below). Placing the 6xHis tag at the C-terminus will select for full-length proteins during purification.

Codon usage

Some codons are rarely used in *E. coli*; for example the arginine codons AGG and AGA are the least frequently used in *E. coli*, and the tRNAs that recognize them are among the least abundant. Consecutive AGG or AGA codons can lead to a high level of frame shifting (Rosenberg et al. 1993). If the DNA sequence encoding the recombinant protein contains several such codons, a variety of truncated protein products, particularly from large recombinant proteins, are likely to be synthesized. By placing the 6xHis affinity tag at the C-terminus, only the full-length proteins will bind to the Ni-NTA resin during the purification procedure; the truncated forms will not bind and will be removed in the flow-through and the wash fractions.

Internal start sites

Initiation of translation at internal start sites can occur when a ribosome binding consensus sequence (Shine-Dalgarno sequence) is present 5' to an internal ATG or GTG codon. Placing the tag at the N-terminus of the protein will prevent the copurification of shorter proteins whose synthesis is initiated at these start sites, but internal starts should be eliminated by deleting any Shine-Dalgarno sequence in the coding region.

Inefficient translation

Some DNA sequences contain regions which interfere with the interaction between the *E. coli* ribosomes and the ribosome binding site provided by the expression vector. This may be the result of stable stem-loop structures formed in the presence of inverted repeats. In most cases this interference can be minimized by modifying the 5' end of the insert by increasing the A–T content, or by constructing a fusion protein in which the sequence is inserted at the 3' end of a fusion partner such as the DHFR protein (pQE-16 or pQE-40 construct). Placing the 6xHis-tag sequence at the 5' end of the gene often increases expression levels.

Secretion

Secretion of proteins in *E. coli* is mediated by an N-terminal signal sequence that is cleaved after protein translocation. Expression of certain gene products as secreted proteins may in some cases be necessary to promote proper folding and disulfide bond formation or to direct toxic proteins out of the cell. The 6xHis tag must be placed at the C-terminus to prevent it from interfering with the signal sequence and to prevent its loss during N-terminal processing. The location of the 6xHis tag at the C-terminus has no effect on secretion. One major drawback of secretion into the periplasm is the lower yield that is generally obtained due to the hydrophobic nature of the signal peptide. Expression may also be complicated by the formation of inclusion bodies in the periplasmic space (Bowden and Georgiou 1990).

Cloning procedures and vector maps

Procedure for direct cloning of PCR fragments using pQE-30 UA

The pQE-30 UA expression vector is designed for direct cloning of PCR products. UA-cloning technology exploits the fact that *Taq* DNA polymerase and other non-proofreading DNA polymerases add a 3'-end A overhang to PCR products. This allows the direct insertion of such PCR products into the prelinearized pQE-30 UA vector, which has a U overhang on each 3' end. This eliminates the need for restriction digestion of the vector or insert, primers with built-in restriction sites, or specially designed adapters, resulting in a much more efficient and robust cloning procedure. Ligation is performed using the 2x Ligation Master Mix supplied with pQE-30 UA vector in the QIA*express* UA Cloning Kit. The Master Mix contains all reagents and cofactors required for efficient ligation (see page 26 for protocol). The pQE-30 UA expression vector allows ampicillin selection. The vector contains several unique restriction-endonuclease recognition sites around the cloning site allowing easy restriction analysis of recombinant plasmids. The first three bases at the 5' end of the PCR product constitute the first codon that will be expressed from the insert. The MCS sequence of pQE-30 UA can be found on page 117 and online at **www.qiagen.com/literature/vectors.asp**.

PCR products generated using proofreading DNA polymerases can be used in UA cloning procedures after the addition of a 3'-end A overhang. The QIAGEN A-Addition Kit (cat. no. 231994) provides an easy and efficient method to modify blunt-ended PCR products. Optimized QIAGEN A-Addition Master Mix allows fast and efficient addition of A residues to blunt-ended PCR products in a convenient pre-mixed format.

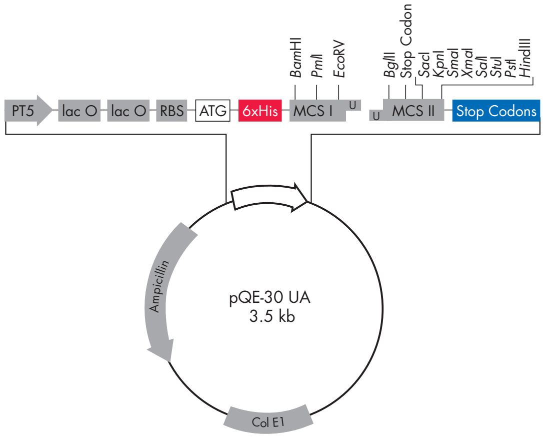

Figure 7. *pQE-30 UA vector for direct cloning of PCR products into an expression vector.* ***PT5****: T5 promoter,* ***lac O****: lac operator,* ***RBS****: ribosome binding site,* ***ATG****: start codon,* ***6xHis****: His tag sequence,* ***MCSI/MCSII****: multiple cloning sites,* ***Stop Codons****: stop codons in all three reading frames,* ***Col E1****: Col E1 origin of replication,* ***Ampicillin****: ampicillin resistance gene.*

Cloning PCR products into Vector pQE-30 UA

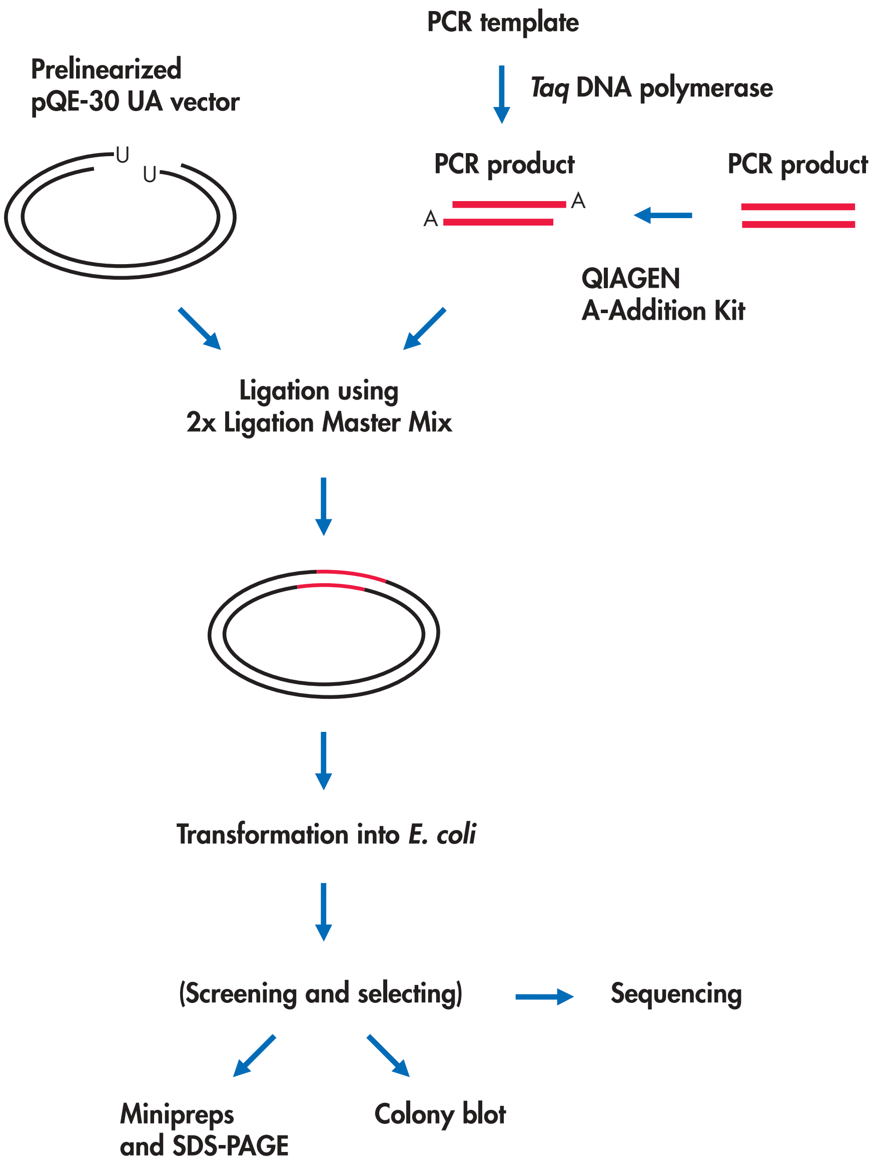

Figure 8. *Construction of QIAexpress Expression vectors using vector pQE-30 UA and PCR products.*

Cloning

Protocol 1. Ligation with pQE-30 UA

Important notes before starting

- Use of PCR products generated using proofreading DNA polymerases (i.e., DNA polymerases with 3'–5' exonuclease activity) will dramatically lower ligation efficiency as these PCR products do not have an A overhang. 3'-end A tailing of proofreading-DNA polymerase–amplified PCR fragments can be performed using the QIAGEN A-Addition Kit (see Ordering Information, page 122).
- Purification of PCR products prior to ligation (e.g., using QIAquick® or MinElute™ PCR Purification or Gel Extraction Kits) is recommended and will generally result in higher transformation efficiencies.
- Background colonies may appear following transformation if the PCR template was plasmid DNA containing a resistance gene for the antibiotic used for colony selection (i.e., an ampicillin- resistance gene). In these cases the PCR product should be gel-purified prior to ligation to remove template plasmid DNA. Gel-purification is not necessary if the template plasmid contains no ampicillin-resistance gene.
- We recommend using a 5–10-fold molar excess of PCR product DNA over pQE-30 UA vector DNA for ligation. In some cases, a lower ratio of PCR product to vector may be sufficient for efficient ligation. Table 2 shows the amount of differently-sized PCR products corresponding to a 5- and 10-fold molar excess over 50 ng pQE-30 UA cloning vector.

Table 2. Guide for the amount of PCR product to use in the ligation reaction

	Amount of PCR Product to be used in Ligation Reaction*	
Size of PCR Product	5-fold molar excess	10-fold molar excess
100 bp	7 ng	14 ng
200 bp	14 ng	28 ng
500 bp	35 ng	70 ng
1000 bp	70 ng	140 ng
1500 bp	105 ng	210 ng
2000 bp	140 ng	280 ng
3000 bp	210 ng	420 ng

* *Calculated for 50 ng pQE-30 UA cloning/expression vector using the following equation:*

$$\text{ng PCR product required} = \frac{\text{50 ng x PCR product size (bp) x molar ratio}}{\text{3504 bp}}$$

Procedure

1. **Thaw 2x Ligation Master-Mix, pQE-30 UA expression cloning vector DNA, and distilled water (provided). Place on ice after thawing.**

   It is important to mix the solutions completely before use to avoid localized concentrations of salts. Keep 2x Ligation Master Mix on ice and store immediately at –20°C or –70°C after use.

2. **Prepare a ligation-reaction mixture according to the following scheme:**

Component	Volume/reaction	Final concentration
pQE-30 UA vector (50 ng/µl)	1 µl	5 ng/µl
PCR product	1–4 µl*	–
Distilled water	Adjust to 5 µl	–
2x Ligation Master Mix†	5 µl	1x
Total volume	10 µl	–

* *Purified PCR product. If using non-purified PCR product, do not add more than 2 µl PCR product.*
† *We recommend adding the Ligation Master Mix last.*

3. **Briefly mix the ligation-reaction mixture and incubate the tube at 16°C, (e.g., in a water bath or thermal cycling block), for 2 hours.**

   Mix gently, e.g., by pipetting the ligation-reaction mixture up and down a few times.

4. **Proceed with the Transformation Protocol or store ligation-reaction mixture at –20°C until use.**

   Note: If transforming cells by electroporation we recommend incubation of the ligation reaction for 10 min at 72°C. Before electroporation the ligation reaction should be briefly centrifuged. Heat treatment increases transformation efficiency. Heat treatment is not necessary if transformation is carried out using other methods.

Troubleshooting: UA cloning

	Comments and suggestions
**Ligation efficiency is too low**	
PCR cloning insert lacks 3'-end A overhangs.	Add 3'-end A extensions to the cloning insert using QIAGEN A-Addition Kit.
Contaminants are present in the insert preparation, which reduce ligase and transformation efficiency.	Remove phenol, ethanol, salts, proteins or detergents by cleanup of the DNA (e.g., using QIAquick or MinElute PCR Purification or Gel Extraction Kits).
Insert to vector ratio is too low.	Use of a 5–10-fold molar excess of PCR product DNA over pQE-30 UA vector DNA is recommended for ligation. Use the equation for calculating the amount of PCR fragment required if using 50 ng pQE-30 UA vector (see Table 2, Protocol 1 on page 26).
Ligation reaction time is too short.	Extend the ligation reaction time or incubate the ligation mixture overnight at 16°C.
Ligation temperature is incorrect.	Ensure that the ligation temperature does not exceed 16°C. Higher temperatures may give rise to increased background and fewer recombinants.
Nuclease contamination.	Nucleases may degrade the vector U overhang. Use only the water and Ligase Master Mix provided with the kit.
Overexposure of PCR fragment to UV light.	If the PCR fragment is gel-purified, the DNA should be exposed to long-wave UV-light for as short a time as possible. Overexposure of DNA to UV light will lead to the formation of pyrimidine dimers that cannot be ligated efficiently.

Cloning procedures with pQE vectors using restriction enzymes

Preparation of pQE expression constructs

The construction of expression vectors is generally straightforward (Figure 9, page 30). The gene or cDNA sequence encoding the protein sequence to be expressed is cloned into the appropriate pQE vector in the same reading frame as the 6xHis affinity tag. The choice of vector depends on the cloning strategy, where the 6xHis tag is to be placed (N-terminal or C-terminal), whether the protein is to be expressed as a fusion protein with DHFR, and how the DNA fragment will be inserted in the correct reading frame.

The pQE expression construct is created by ligation and then transformed into the M15 or other host strain carrying the pREP4 repressor plasmid. Transformants are selected on plates containing both ampicillin and kanamycin. It is recommended that those unfamiliar with these procedures consult a practical manual, such as *Molecular Cloning: a Laboratory Manual* (Sambrook et al. 1989), or *Current Protocols in Molecular Biology* (Ausubel et al. 1995). If pQE-80L, pQE-81L, or pQE-82L are used, the presence of pREP4 in the host strain is not necessary.

Cloning

Vector preparation

The vector from the QIA*express* Kit is prepared by first dissolving it in TE buffer. The addition of 10 µl TE to 5 µg of plasmid DNA is generally convenient. A 2 µl aliquot (i.e. 1 µg) can then be linearized using the appropriate restriction enzyme according to the enzyme manufacturer's recommended buffer and incubation conditions. Many combinations of enzymes are compatible when used together in the same buffer, but different enzymes cut with different efficiencies, especially when the two sites are close together. If restriction enzymes are sufficiently active in a given buffer and their sites are more than 10 bp apart, they can be used in the same reaction. If this is not the case, the digestion should be carried out separately with a clean-up step in between. QIAquick® Kits provide a very fast and efficient method to purify DNA.

Expression vectors such as the pQE plasmids do not allow color selection of clones that contain plasmids with inserts. Care should therefore be taken to ensure that vectors are digested to completion before subcloning. If the insert is to be cloned into a single restriction site, it is especially important to dephosphorylate the vector ends after digestion. In vectors cut with two enzymes, when the sites are close together or if one of the enzymes cuts inefficiently, dephosphorylation decreases the nonrecombinant background caused by incomplete digestion with one of the enzymes. Following digestion it is usually worthwhile to gel-purify the vector prior to insert ligation in order to remove residual nicked and supercoiled plasmid. The latter will transform *E. coli* much more efficiently than ligated plasmids. Positive expression clones can be detected directly on colony blots using one of the Anti·His Antibodies or Ni-NTA Conjugates (see Protocol 4 on page 41).

Cloning target sequence into QIA*express* vectors

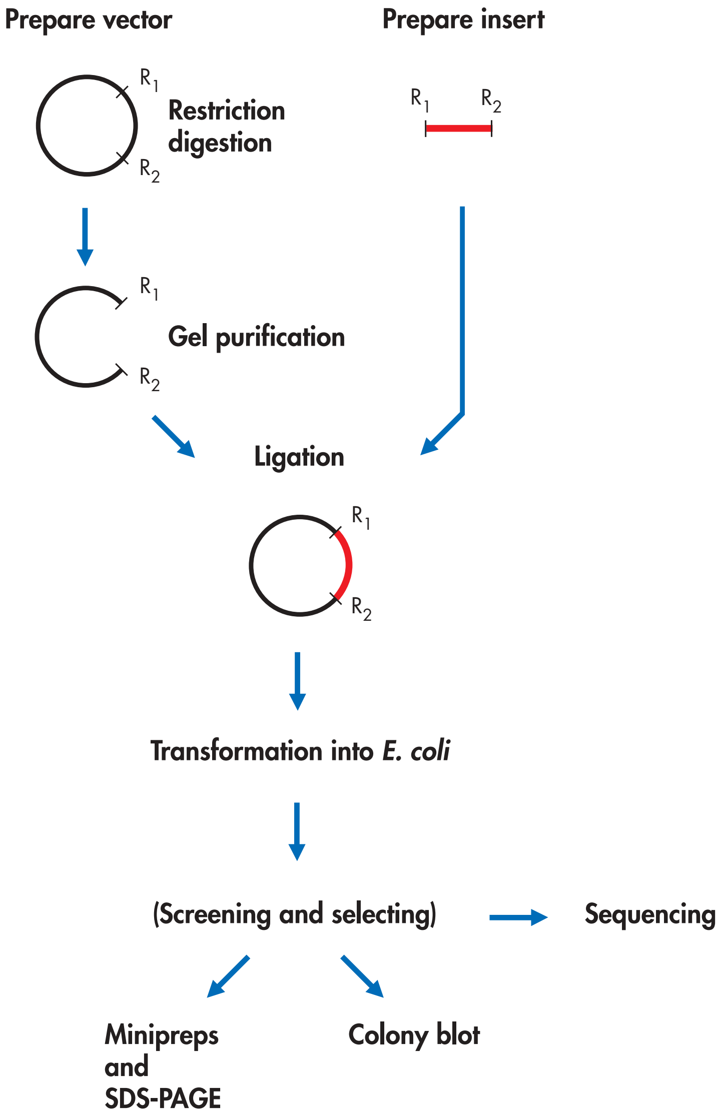

Figure 9. *Construction of QIA*express *expression vectors.* ***R_1, R_2****: restriction sites.*

Insert preparation

In general, it is only necessary to prepare the insert by restriction digestion and gel purification. The fragment can be ligated to the vector directly. If there is no appropriate restriction site, if it is desirable to minimize the number of extra codons, or if the construct must be optimized in some other way (see specific construct sections on page 32), more complicated manipulations may be necessary. The ends of coding fragments can be modified by PCR, by in vitro mutagenesis, or by the addition of linkers (Ausubel et al. 1995; Sambrook et al. 1989).

N-terminal 6xHis tag constructs

The relevant pQE vectors for these constructs contain the 6xHis tag (plus DHFR, in the case of pQE-40) 5' to the polylinker (Figure 11, page 33). The appropriate vector must only be digested with the necessary restriction enzyme(s), purified to obtain the linear form, and ligated with the insert containing the desired coding region. If only one restriction site is used, we recommend that the ends of the vector be dephosphorylated to prevent religation.

The ribosome binding site (Shine-Dalgarno sequence) and the ATG initiation codon should be removed from the fragment that is to be inserted into these vectors. Internal starts from control sequences provided by the inserted fragment itself will result in the expression of proteins that lack the 6xHis tag and thus cannot be purified. The endogenous stop codons can be retained in the inserted fragment, but are not required since pQE vectors provide translational stop codons in all three reading frames. The pQE-80L series of vectors is similar to the pQE-30 series, but also encodes a *cis-lacI*^{q} gene — this eliminates the need to use pREP4 for repression in *trans*.

pQE-30 Xa

The pQE-30 Xa vector is similar to pQE-30, but also encodes a Factor Xa Protease recognition site which is bracketed by the 6xHis-tag coding region on the 5' side and the multiple cloning site on the 3' side (Figure 10, page 32). 5'-end cloning using the blunt-end *Stu*I restriction site allows insertion of the gene of interest directly behind the Factor Xa Protease recognition site, without any intervening amino acid codons. Factor Xa Protease cleaves off the 6xHis-tag peptide behind the arginine residue of the protease recognition site (IEGR↓). Factor Xa Protease treatment results in a recombinant protein free of any vector-derived amino acids at the N-terminus.

TAGZyme™ pQE-1 and pQE-2

The two expression vectors TAGZyme pQE-1 and pQE-2 greatly facilitate the production of His-tag free proteins by encoding an N-terminal 6xHis tag that possesses a sequence optimized for TAGZyme Enzyme digestion. For more information on the TAGZyme System for exoproteolytic tag removal, call QIAGEN Technical Services or your local distributor.

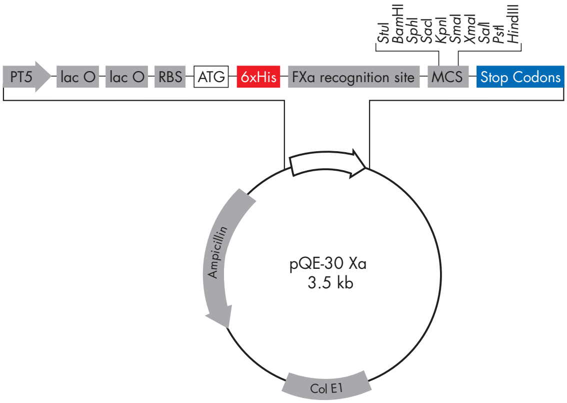

Figure 10. *pQE-30 Xa vector for the insertion of a Factor Xa Protease recognition site C-terminal of the 6xHis tag.* ***FXa recognition site****: Factor Xa Protease recognition site.*

C-terminal 6xHis tag constructs

The C-Terminus pQE Vector Set consists of pQE vectors that place the 6xHis tag at the C-terminus of expressed proteins. Inserts cloned into pQE-16 must be in frame with both the 5' start codon and the 3' sequences encoding the 6xHis tag. The DHFR sequence can be fused to the 6xHis-tagged protein or removed using the *Bam*HI and *Bgl*II restriction sites.

Type ATG constructs allow the expressed protein to initiate with the authentic ATG, but using the optimized Shine-Dalgarno region of the pQE vector. They are prepared in the same way as pQE-16 constructs, with all the same considerations, except that the sequence around the authentic ATG must be modified to create either an *Nco*I site (pQE-60) or an *Sph*I site (pQE-70) (Figure 12, page 34). Cleavage of both the vector and the coding fragment with the appropriate enzyme allows the coding fragment to be ligated into the vector such that the authentic ATG codon replaces the vector ATG codon. If the 6xHis tag cannot be cloned in the correct reading frame by restriction cloning, the insert can be amplified by PCR incorporating appropriate 5' and 3' restriction sites into the PCR primers.

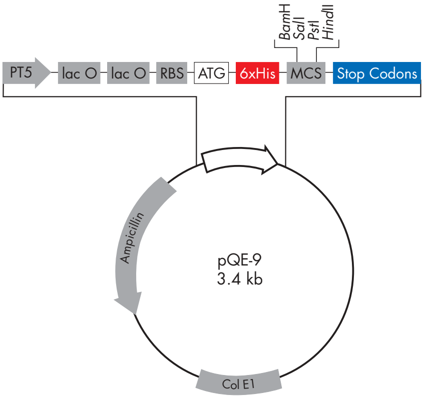

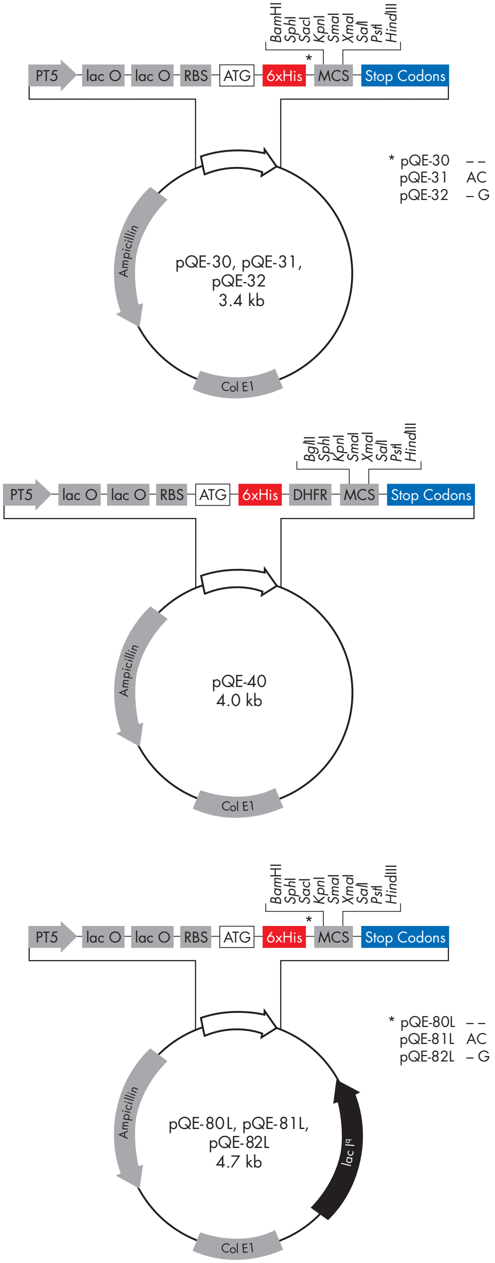

Figure 11. *pQE vectors for N-terminal 6xHis tag constructs (see also opposite page).* ***PT5****: T5 promoter,* ***lac O****: lac operator,* ***RBS****: ribosome-binding site,* ***ATG****: start codon,* ***6xHis****: 6xHis tag sequence,* ***MCS****: multiple cloning site with restriction sites indicated,* ***Stop Codons****: stop codons in all three reading frames,* ***Col E1****: Col E1 origin of replication,* ***Ampicillin****: ampicillin resistance gene,* $\boldsymbol{lacI^q}$, *$lacI^q$ repressor gene.*

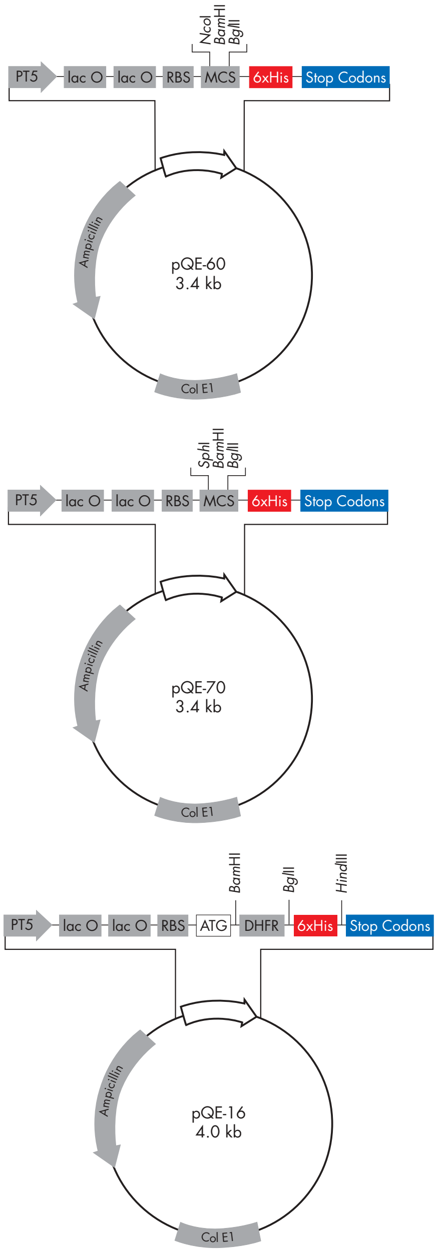

Figure 12. *pQE vectors for C-terminal 6xHis tag constructs.* ***PT5****: T5 promoter,* ***lac O****: lac operator,* ***RBS****: ribosome-binding site,* ***ATG****: start codon,* ***6xHis****: 6xHis tag sequence,* ***MCS****: multiple cloning site with restriction sites indicated,* ***Stop Codons****: stop codons in all three reading frames,* ***Col E1****: Col E1 origin of replication,* ***Ampicillin****: ampicillin resistance gene.*

DoubleTag constructs

The pQE-100 DoubleTag Vector places a tag at each end of expressed proteins. The 6xHis tag is at the N-terminus and the Tag·100 epitope at the C-terminus. The insert needs to be cloned in frame at the 5' and 3' ends. The Tag·100 epitope coded by the pQE-100 DoubleTag Vector is derived from MAP kinase 2 and has the amino acid sequence EETARFQPGYRS.

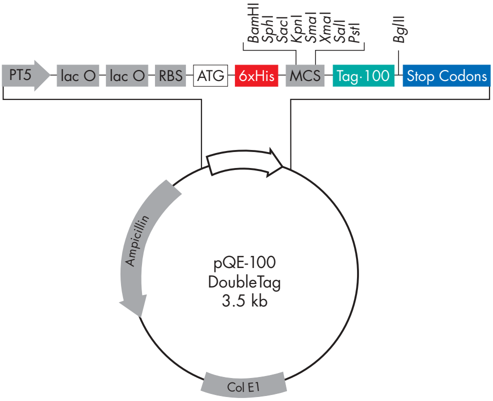

Figure 13. *pQE-100 DoubleTag Vector for double-tagged constructs.* ***PT5****: T5 promoter,* ***lac O****: lac operator,* ***RBS****: ribosome-binding site,* ***ATG****: start codon,* ***6xHis****: 6xHis tag sequence,* ***MCS****: multiple cloning site with restriction sites indicated,* ***Tag·100****: Tag·100 epitope,* ***Stop Codons****: stop codons in all three reading frames,* ***Col E1****: Col E1 origin of replication,* ***Ampicillin****: ampicillin resistance gene.*

Propagation of pQE plasmids and constructs

QIA*express* pQE vectors and constructs can be maintained in any *E. coli* strain that is ampicillin-sensitive and carries the pREP4 repressor plasmid, or harbors the *lacI*^{q} gene on the F-factor episome. M15 and SG13009 carry *lacI* on the plasmid pREP4, while XL1-Blue or the JM series contain an episomal copy of *lacI*^{q}. *lacI*^{q} is a mutation of *lacI* that produces very high levels of the *lac* repressor. Initial cloning and propagation using XL1-Blue is recommended because plasmid preparations derived from QIA*express* host strains will also contain pREP4 DNA, which could make clone analysis more difficult. Alternatively, the pQE-80L series of expression vectors which encodes a *lacI*^{q} repression module, allows use of any *E. coli* host strain.

For the most stable propagation of expression constructs, especially when toxic proteins are expressed, we recommend using the M15[pREP4] strain because of the higher repressor levels. In this case, both ampicillin and kanamycin must be applied to maintain the expression and repressor constructs.

Ligation, transformation, and screening

The ligation of the insert with the prepared vector is usually carried out using T4 DNA ligase under standard conditions (Sambrook et al. 1989). Ligation of PCR inserts into the pQE-UA vector is carried out using the 2x Ligation Master Mix provided in the QIA*express* UA Cloning Kit (see ligation protocol, page 26).

A procedure for the transformation of M15[pREP4] cells is presented on page 40, but any standard procedure may be used. The efficiency of the subcloning procedure should always be monitored by the transformation of nonligated and self-ligated vector controls. A control transformation without DNA should be performed to ensure that the antibiotic ampicillin is working effectively, and a control of transformation efficiency with a known amount of intact plasmid DNA (e.g., 1 or 10 ng per transformation) should also be included.

Transformants may be screened for correct insertion of the coding fragment by restriction analysis of the pQE plasmid DNA, sequencing of the cloning junctions, by directly screening bacterial colonies for the expressed protein (colony-blotting procedure) or by preparing small-scale expression cultures. We prefer and recommend screening the transformants directly for expression using the colony-blotting procedure because it allows simultaneous screening of transformants with the correct coding fragment, expression levels, and in-frame translation of the 6xHis tag. Clones with religated vectors that do not express a fusion protein will not generate a false positive signal. The colony-blot protocol for detecting expressed proteins is described on page 41. A protocol for direct screening of mini expression cultures is presented on page 45. Information about the priming sites of appropriate sequencing primers for the cloning region of pQE vectors can be found in the appendix on page 118.

Integration of 6xHis tags into other vectors

The 6xHis tag can be easily incorporated into any expression vector using a variety of methods including mutagenesis, PCR, or fragment integration. The linker fragment below can be created from synthetic oligonucleotides, with overlapping ends corresponding to the appropriate restriction site(s) and cloned into an appropriated location in the insert or in a vector sequence.

```
5'-Oligo    5'- XXXXX CAT CAC CAT CAC CAT CAC X     -3'
3'-Oligo    3'-     X GTA GTG GTA GTG GTA GTG XXXXX -5'
                      His His His His His His
```

It should be noted that by using a modified form of these oligonucleotides (shown below), the epitope RGS(His)$_4$ can be incorporated with the 6xHis tag to enable detection of the recombinant protein using the RGS·His antibody on colony, dot, or western blots and in complex applications such as immunocytochemistry, which requires high antibody specificity.

```
5'-Oligo    5'- XXXXX AGA GGA TCG CAT CAC CAT CAC CAT CAC X     -3'
3'-Oligo    3'-     X TCT CCT AGC GTA GTG GTA GTG GTA GTG XXXXX -5'
                      Arg Gly Ser His His His His His His
```

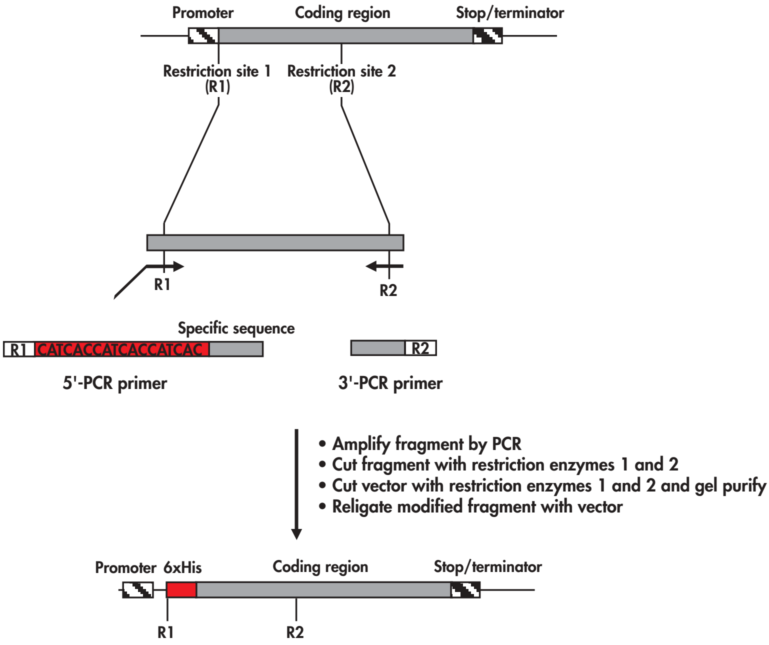

Figure 14. *Modification of inserts by PCR.*

PCR mutagenesis to insert 6xHis tag

Alternatively, if the expression vector or the existing construct being used does not encode a 6xHis tag, the tag can be easily engineered into the insert sequence itself by employing PCR techniques (Figure 14, page 37). One PCR primer includes the 5' restriction site (R1), the CAT CAC CAT CAC CAT CAC sequence for the 6xHis tag, and a priming sequence that is homologous to the 5' end of the insert DNA corresponding to the N-terminus of the protein. The second primer contains another restriction site (R2) which is located within the insert. The PCR product and the original construct are then both cut with the enzymes R1 and R2 and ligated together. The recombinant plasmid encoding the 6xHis-tagged protein is then used for transformations or transfections. A C-terminal 6xHis tag can be inserted into a construct following a similar approach and using an appropriate set of primers. If you are unfamiliar with PCR techniques, consult the technical guidelines for "Constructing Recombinant DNA Molecules by the Polymerase Chain Reaction" (Ausubel et al., 1995) or refer to the Taq *PCR Handbook* available at **www.qiagen.com/literature/handbooks**.

Protocol 2. Preparation of competent *E. coli*

Materials

M15[pREP4] cells

LB medium

LB agar

Psi broth

TFB1

TFB2

Kanamycin stock solution

Ampicillin stock solution

For composition of media and solutions, see appendix, page 111.

1. **Remove a trace of M15[pREP4] cells from the vial with a sterile toothpick or inoculating loop, and streak it out on LB agar containing 25 µg/ml kanamycin.**

   If the host strain has already been cultured and stored as recommended on page 8, streak out bacteria from those stocks.
2. **Incubate at 37°C overnight.**
3. **Pick a single colony and inoculate 10 ml of LB-kanamycin (25 µg/ml). Grow overnight at 37°C.**
4. **Add 1 ml overnight culture to 100 ml prewarmed LB medium containing 25 µg/ml kanamycin in a 250 ml flask, and shake at 37°C until an OD_{600} of 0.5 is reached (approximately 90–120 min).**
5. **Cool the culture on ice for 5 min, and transfer the culture to a sterile, round-bottom centrifuge tube.**
6. **Collect the cells by centrifugation at low speed (5 min, 4000 x *g*, 4°C).**
7. **Discard the supernatant carefully. Always keep the cells on ice.**
8. **Resuspend the cells gently in cold (4°C) TFB1 buffer (30 ml for a 100 ml culture) and keep the suspension on ice for an additional 90 min.**
9. **Collect the cells by centrifugation (5 min, 4000 x *g*, 4°C).**
10. **Discard the supernatant carefully. Always keep the cells on ice.**
11. **Resuspend the cells carefully in 4 ml ice-cold TFB2 buffer.**
12. **Prepare aliquots of 100–200 µl in sterile microcentrifuge tubes and freeze in liquid nitrogen or a dry-ice–ethanol mix. Store the competent cells at –70°C.**

Protocol 3. Transformation of competent M15 cells

1. **Transfer an aliquot of the ligation mix (10 µl or less) into a cold sterile 1.5 ml microcentrifuge tube, and keep it on ice.**
2. **Thaw an aliquot of frozen competent M15[pREP4] cells on ice.**
3. **Gently resuspend the cells and transfer 100 µl of the cell suspension into the microcentrifuge tube with the ligation mix, mix carefully, and keep it on ice for 20 min.**
4. **Transfer the tube to a 42°C water bath or heating block for 90 sec.**
5. **Add 500 µl Psi broth to the cells and incubate for 60–90 min at 37°C.**
   Shaking increases transformation efficiency.
6. **Plate out 50, 100, and 200 µl aliquots on LB-agar plates containing 25 µg/ml kanamycin and 100 µg/ml ampicillin (or another selective antibiotic, such as carbenicillin). Incubate the plates at 37°C overnight.**

Positive control to check transformation efficiency:

Transform competent cells with 1 ng of the pQE-40 control plasmid (undigested) in 20 µl of TE. Plate 1/100 and 1/10 dilutions of the transformation mix (diluted in prewarmed Psi broth) as well as undiluted transformation mix on LB-agar plates containing 25 µg/ml kanamycin and 100 µg/ml ampicillin. The cells should yield 10^6 transformants per microgram of plasmid.

Negative control to check antibiotic activity:

Transform cells with 20 µl of TE. Plate at least 200 µl of the transformation mix on a single plate.

Analysis of transformants

Protocol 4. Colony-blot procedure

The colony-blot procedure (Figure 15) is used for the identification of clones expressing a 6xHis-tagged protein. We describe the procedure using an Anti·His Antibody or Ni-NTA Conjugate because in many instances an antibody specific for the target protein is not available, and the Anti·His Antibody and Ni-NTA Conjugate also detect the presence of the 6xHis tag. However, in principle, the same protocol can be used if a protein-specific antiserum or monoclonal antibody is available. The procedure described can distinguish clones that express a 6xHis-tagged protein from those that express the short peptide sequence encoded by pQE plasmids lacking an insert. Small peptides (<30 amino acids) expressed from QIA*express* vectors pQE-9, -30, -31, -32, -60, -70, -80L, -81L, and -82L are degraded within the cells and will not yield a positive signal in the detection procedure. pQE-40, which encodes the RGS·His epitope at the N-terminus of the 26 kD DHFR protein, can be used to generate positive controls.

The Anti·His Antibodies are very specific and have very high binding constants. Stringent wash steps will aid in avoiding nonspecific signals. If these persist, it is often a result of the quality or concentration of the secondary antibody used in conjunction with the Anti·His Antibody. To differentiate between specific and nonspecific signals, *E. coli* harboring the plasmid without the insert should also be plated and treated the same way as the transformants with inserts.

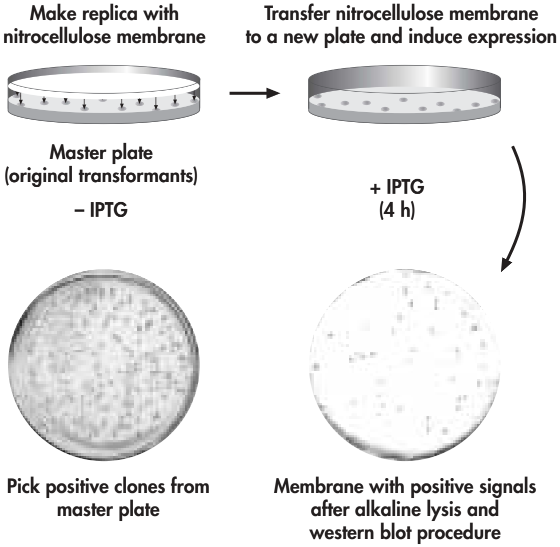

Figure 15. *Detection of positive expression clones by colony blotting.*

Some nonspecific cross-reactivity with endogenous *E. coli* proteins may be encountered, especially when polyclonal antibodies raised against the target protein are being used. In such cases it may be necessary to preadsorb the serum against an *E. coli* lysate to remove anti-*E. coli* antibodies (Sambrook et al. 1989, pp. 12.25–12.28).

Materials and reagents

LB-agar plates with relevant antibiotics

LB-agar plates with relevant antibiotics and 250 µM IPTG

Nitrocellulose filter discs

Syringe needle (with India ink — optional)

SDS solution

Denaturing solution

Neutralization solution

20x SSC

TBS buffer

TBS-Tween® buffer (for Ni-NTA Conjugates)

TBS-Tween/Triton® buffer (for Anti·His Antibodies or Ni-NTA Conjugates)

Blocking buffer

Alkaline phosphatase or horseradish peroxidase staining solutions

Stock solution of primary antibody (Anti·His Antibody) or Ni-NTA Conjugate

Stock solution of secondary AP- or HRP-conjugated antibody (when RGS·His Antibody is used).

For buffer and reagent compositions, see appendix, page 111.

1. **Plate the transformation mix on LB plates containing the relevant antibiotics and incubate them overnight (16 h) at 30°C until the colonies are about 1–2 mm in diameter.**

   After spreading the transformation mix, dry the plates inverted with the lids slightly open until small wrinkles develop on the surface of the agar. To prevent streaking, incubation should not be started until all of the liquid has been absorbed into the agar.

   To reduce expression of toxic proteins in the absence of IPTG induction (due to "leaky" promoters) and to maintain plasmid stability, incubation should be carried out at 30°C. If the expressed protein is not toxic and the plasmids are stable, incubation can be carried out at 37°C, but care should be taken that the colonies do not become too large.

2. **Remove plates from the incubator, open lids slightly, and allow any condensation to dry for 15–30 min.**

3. **Place a dry, numbered nitrocellulose filter on the agar surface in contact with the colonies, taking care to avoid introducing air bubbles under the filter.**

   Number filters with a water-resistant marking pen or pencil. Hold the filter on opposite sides with two blunt-ended forceps so that it is curved up, and align its diameter with the diameter of the plate. Lower the filter gently onto the agar surface, making contact first along the diameter and then lowering (but not dropping) the sides.

4. **Using a syringe needle, pierce the filter and agar at asymmetric positions to facilitate proper alignment following staining. Grip the filter on the sides with blunt-ended forceps and peel it off in one movement.**

5. **Transfer the filter (colony side up!) to a fresh plate containing antibiotics and IPTG as described above. Avoid introducing air bubbles.**

6. **Incubate the plates inverted for 4 h at 37°C to induce expression. Place the original master plates in the incubator to allow the colonies to regrow.**

7. **Prepare a set of polystyrene dishes for colony lysis and binding of protein to the filters.**

   Each dish should contain a sheet of 3MM paper soaked with one of the following solutions:

   SDS Solution

   Denaturing solution

   Neutralization solution

   Neutralization solution

   2x SSC

   Note: discard excess fluid so that the paper is moist but not wet. Excess liquid promotes colony swelling and diffusion which will result in blurred signals.

8. **Place the nitrocellulose filters (colony side up!) on top of the paper in each of these dishes, taking care to exclude air bubbles (colonies above air bubbles will not lyse properly and will generate a higher level of background in the final staining step).**

9. **Incubate the filters at room temperature as follows:**

SDS solution	10 min
Denaturing solution	5 min
Neutralization solution	5 min
Neutralization solution	5 min
2x SSC	15 min

10. **Wash filters twice for 10 min with TBS buffer.**

11. **Incubate for 1 h in blocking buffer at room temperature.**

12. **Wash twice for 10 min in TBS-Tween/Triton buffer.**

13. **Wash for 10 min in TBS buffer.**

14. **Incubate in RGS·His Antibody solution (1/1000 or 1/2000 dilution in blocking buffer) or conjugate solution (1/1000 dilution in TBS-Tween) at room temperature for 1 h.**

    At this stage, the filter membrane can be sealed in a plastic bag.

15. **Wash twice for 10 min in TBS-Tween/Triton buffer (antibody) or TBS-Tween (conjugates).**

16. **Wash for 10 min in TBS buffer.**

    If a conjugate is being used, proceed to step 19.

17. **Incubate the filters with 10 ml secondary antibody solution (diluted in blocking buffer) for 1 h at room temperature.**

    Both alkaline phosphatase (AP) or horseradish peroxidase (HRP) conjugated rabbit or goat anti-mouse IgG may be used. Dilute the secondary antibody according to the manufacturer's recommendations. Use the lowest recommended concentration to avoid false signals.

18. **Wash 3 x 10 min in TBS-Tween/Triton buffer.**

19. **Stain with AP or HRP staining solution until the signal is clearly visible.**

    Note: There is often only a slight difference between colonies showing a positive signal and background signals. Staining times may differ with this procedure. 2–3 min is usually sufficient, but it is very important to monitor color development.

20. **Stop the reaction by rinsing the membrane twice with water.**

If it is extremely difficult to differentiate between positive clones and background, the cause of the high background should be determined. The following controls should be included:

- A plate with the host bacteria lacking the expression plasmid
- A plate of host bacteria harboring the expression plasmid lacking the insert
- A colony blot treated only with the secondary antibody prior to staining
- A positive control expressing 6xHis-tagged DHFR from pQE-40, if possible

Protocol 5. Rapid screening of small expression cultures

The following is a basic protocol for the expression and screening of small cultures by purification of 6xHis-tagged proteins on Ni-NTA Spin Columns. Purification is performed under denaturing conditions to isolate any tagged proteins, independent of their solubility within the cell. The 6xHis tag will also be completely exposed under denaturing conditions, which increases binding and therefore yields when compared to the purification under native conditions.

Lysing cells in buffer B allows solubilization of most proteins and inclusion bodies, and facilitates their direct analysis by SDS-PAGE. To solubilize very hydrophobic receptor or membrane proteins, buffer A, containing guanidine HCl (GuHCl), must be used, sometimes in combination with detergents.

The following is a basic protocol for the expression and screening of small cultures. The protocol does not include stringent washing steps, and therefore cannot be expected to give a very pure product, but it will indicate whether the protein of interest is being expressed with the 6xHis tag and will reflect the level of expression. The procedure is performed under denaturing conditions, which will lead to the isolation of any tagged protein, independent of its location within the cell.

This procedure may also be used to perform a time course. When analyzing the time course of expression, it is best to begin with a 100 ml culture in a flask, and to take 10 ml samples at 0, 1, 2, 3, and 4 h after induction.

Culture media should contain ampicillin at 100 µg/ml and kanamycin at 25 µg/ml.

Materials

Ni-NTA Spin Columns

LB medium

Kanamycin stock solution

Ampicillin stock solution

IPTG stock solution

Buffers A–D

5x SDS-PAGE sample buffer

For composition of buffers and solutions, see appendix, page 111.

Cloning

1. **Pick single colonies of transformants into 1.5 ml of culture media containing both ampicillin (100 µg/ml) and kanamycin (25 µg/ml). Also inoculate one 1.5 ml culture with a colony transformed with the control plasmid pQE-40, which expresses 6xHis-tagged DHFR or another appropriate control. Inoculate one extra culture to serve as a noninduced control. Grow the cultures overnight.**
2. **Inoculate 10 ml of prewarmed medium (including antibiotics) with 500 µl of the overnight cultures, and grow at 37°C for 30 min, with vigorous shaking, until the OD_{600} is 0.5–0.7.**

   The short second growth from an aliquot of the saturated culture, ensures that all cultures are grown to a similar cell density before induction.

   Larger cultures can be scaled up accordingly. If expression levels are very low (<1 mg/liter), it may be necessary to purify the protein from up to 50 ml culture.
3. **Induce expression by adding IPTG to a final concentration of 1 mM.**

   Do not add IPTG to the culture which will serve as a noninduced control. If a time course of expression is being taken, the t=0 sample serves as the noninduced control.
4. **Grow the cultures for an additional 4–5 h, and transfer to microcentrifuge tubes. Harvest the cells by centrifugation for 1 min at 15,000 x *g*, and discard supernatants.**

   If a time course of expression is being performed, take 2 ml samples at hourly intervals, collect the cell pellets, and store at –20°C until all the samples are ready for processing.
5. **Resuspend cells in 400 µl buffer B. Lyse cells by gently vortexing, taking care to avoid frothing.**

   The solution should become translucent when lysis is complete.

   The culture volume used depends on the expected expression level. When the protein is expressed at very high levels, (50–100 mg/liter) a 5x-concentrated cell lysate (resuspend the pellet from a 2 ml culture in 400 µl buffer B) can be used. 400 µl of a 5x-concentrated cell lysate in buffer B will contain approximately 100–200 µg of 6xHis-tagged protein. For lower expression levels (1–5 mg/liter), 10 ml of cell culture should be used for a 25x-concentrated cell lysate. Resuspend the pellet from a 10 ml culture in 0.4 ml buffer B (0.4 ml cell lysate = 10–50 µg) of 6xHis-tagged protein.
6. **Centrifuge the lysate for 20–30 min at 15,000 x *g* to remove cellular debris, and transfer the supernatant to a fresh tube.**
7. **Equilibrate a Ni-NTA spin column with 600 µl buffer B. Centrifuge for 2 min at 2000 rpm (approximately 700 x *g*).**
8. **Load the cleared lysate supernatant containing the 6xHis-tagged protein onto an equilibrated Ni-NTA spin column.**

9. **Centrifuge the Ni-NTA spin column for 2 min at 2000 rpm (approx. 700 x *g*), and collect the flow-through.**

   It is important not to exceed 2000 rpm (approx. 700 x *g*) when centrifuging Ni-NTA Spin Columns. Higher speeds reduce binding time, resulting in inefficient binding.

   Save the flow-through for SDS-PAGE analysis.

10. **Wash the Ni-NTA spin column twice with 600 µl buffer C. Centrifuge for 2 min at 2000 rpm (approx. 700 x *g*).**

    Save the flow-through (wash fractions) for analysis by SDS-PAGE to check the stringency of the wash conditions.

    It may not be necessary to repeat the buffer C wash. The number of wash steps required to obtain highly pure protein is determined primarily by the expression level of the 6xHis-tagged protein. When the expression level is high, two wash steps are usually sufficient for removal of contaminants. For very low expression levels or highly concentrated lysates, three wash steps or washes with buffer D may be required to achieve high purity.

11. **Elute the protein with 2 x 200 µl buffer E. Centrifuge for 2 min at 2000 rpm (approximately 700 x *g*), and collect the eluates.**

    Most of the 6xHis-tagged protein (>80%) should elute in the first 200 µl eluate, especially when proteins smaller than 30 kDa are purified. The remainder will elute in the second 200 µl. If the protein should be more concentrated or if the expected expression level is low, elute in 100–150 µl aliquots and/or do not combine eluates.

12. **Add 2.5 µl of 5x SDS-PAGE sample buffer to 10 µl aliquots of all samples, including the unbound fractions, and boil for 5 min at 95°C.**

13. **Analyze the samples by SDS-PAGE.**

Troubleshooting: cloning

Problems encountered during the cloning steps should be addressed by consulting cloning manuals (Ausubel et al. 1995; Sambrook et al. 1989). Check your sequences, and your ligation and transformation steps, and refer to “Troubleshooting: expression” on page 62 and “Troubleshooting: purification” on page 99.

Expression in *E. coli*

Expression of recombinant proteins can be approached in general by starting with a plasmid that encodes the desired protein, introducing the plasmid into the required host cell, growing the host cells and inducing expression, and ending with cell lysis and SDS-PAGE analysis to verify the presence of the protein (Figure 16). With careful choice of host strains, vectors, and growth conditions, most recombinant proteins can be cloned and expressed at high levels in *E. coli*. However, many polypeptide gene products expressed in *E. coli* accumulate as insoluble aggregates that lack functional activity. Other problems with protein expression may include cell toxicity, protein instability, improper processing or post-translational modification, and inefficient translation.

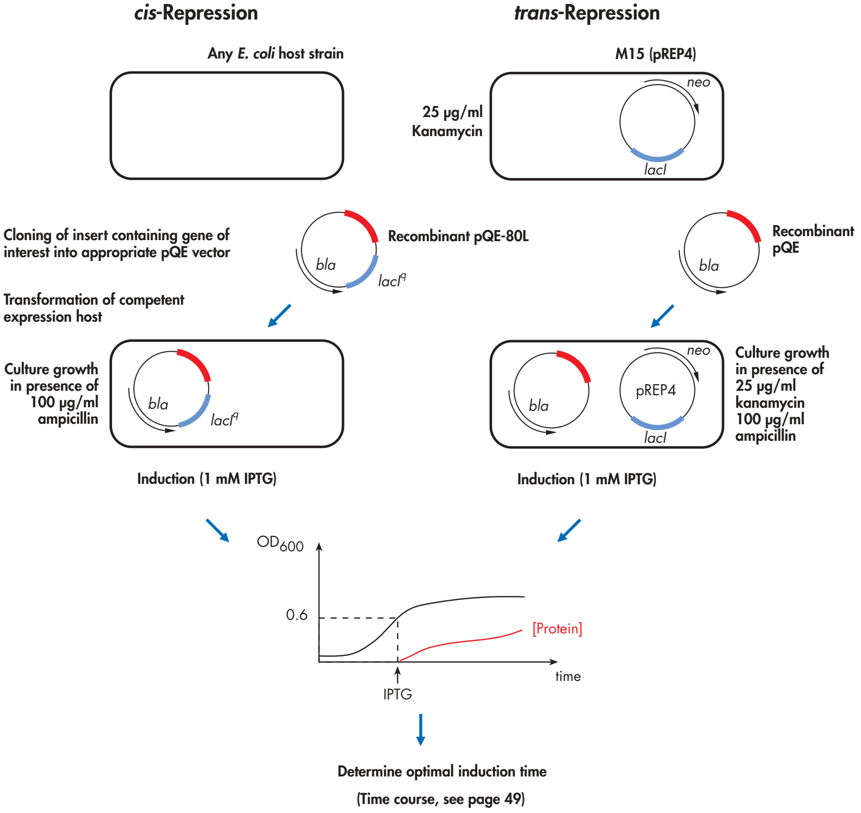

Figure 16. *Expression of 6xHis-tagged proteins with the QIAexpress System.*

A primary consideration for recombinant protein expression and purification is the experimental purpose for which the protein will be utilized. For biochemical and structural studies, it is often important to optimize conditions for the expression of soluble, functionally active protein, whereas for antigen production, the protein can be expressed either in native or denatured form. The QIA*express* System is optimized for high expression levels, but conditions for optimal expression of individual proteins must be determined empirically. Freshly transformed bacterial colonies often express recombinant proteins at different levels. Therefore, comparison of the signals produced after colony blotting to identify high-expressing colonies can help significantly while establishing expression cultures.

Optimal growth and expression conditions for the protein of interest should be established with small-scale cultures before large-scale protein purification is attempted. In order to judge the toxicity of an expressed protein, cell growth before and especially after induction of expression should be monitored. Expression of the nontoxic, 26 kDa DHFR protein encoded by the control plasmid pQE-40 is an ideal control for cell growth, expression, and purification. A much slower growth rate of the host cells expressing the protein of interest suggests that the gene product may be toxic to the *E. coli* host. Conversely, a much faster growth rate may indicate inefficient transcription or translation due to plasmid instability, inefficient induction, or deletions in the control region.

Note: Control plasmid pQE-40 encoding the mouse DHFR is optimized for high-level expression producing up to 40 mg/liter in culture. Since the protein accumulates to almost 90% in an insoluble form in inclusion bodies, purification under denaturing conditions in the presence of urea is recommended. However, approximately 10% of the DHFR protein remains in a soluble form that can be purified under native conditions. The recombinant DHFR protein migrates at 26 kDa on SDS-PAGE gels.

Many factors may contribute to difficulties encountered when expressing foreign proteins in *E. coli*. The following sections address these difficulties in more detail.

Basic principles

Culture media

The media of choice for the growth of M15 cells containing a pQE expression plasmid and the pREP4 repressor plasmid are LB medium and its modifications, 2x YT, or Super Broth, each containing 100 µg/ml ampicillin and 25 µg/ml kanamycin. Initially it is advisable to try expression in all three media in parallel, and to take a time course to monitor growth and expression after induction. Striking differences between the level of expression in different media and at different times are often noted. Using the *cis*-repressed vectors pQE-80L, pQE-81L, or pQE-82L without pREP4 kanamycin should not be included in the growth medium.

Maintenance of the expression plasmid

Poor plasmid maintenance in the cells can lead to low expression levels. Ampicillin is an unstable antibiotic and is rapidly depleted in growing cultures due in part to the β-lactamase secreted by resistant bacterial cells. It is important to check plasmid levels by plating cells from the expression culture on plates with and without ampicillin. If the stability of the expression construct is a problem, the cultures should be grown in the presence of 200 µg/ml ampicillin, and the level should be maintained by supplementing ampicillin during long growth periods. Alternatively, the cultures may be grown in the presence of carbenicillin, a more stable β-lactam, at 50 µg/ml.

Host cells containing the pREP4 repressor plasmid should be maintained in the presence of kanamycin at 25 µg/ml. Many of the *E. coli* host strains that contain the *lacI*^{q} mutation harbor it on an F-factor (see also "Propagation of pQE plasmids and constructs", page 35). Therefore these strains should be checked for the presence of the F-factor before they are transformed with the pQE vectors. For example, XL1-Blue strains can be selected on tetracycline.

Small-scale expression cultures

Small-scale expression and purification experiments are highly recommended and should be performed before proceeding with a large scale preparation. In many cases aliquots of the cells can be lysed in a small volume of sample buffer and analyzed directly by SDS-PAGE. The use of small expression cultures, and the preparation of lysates followed by purification by Ni-NTA affinity chromatography, provide a rapid way to judge the effects of varied growth conditions on expression levels and solubility of recombinant proteins. Expression levels vary between different colonies of freshly transformed cells, and small-scale preparations permit the selection of clones featuring optimal expression rates (see Protocol 5, page 45).

Time-course analysis of protein expression

To optimize the expression of a given protein construct, a time-course analysis of the level of protein expression is recommended (Figure 17). Intracellular protein content is often a balance between the amount of soluble protein in the cells, the formation of inclusion bodies, and protein degradation. By checking the 6xHis-tagged protein present at various times after induction in the soluble and insoluble fractions (see Protocol 14, page 85 and Protocol 19, page 92), the optimal induction period can be established.

A protocol using Ni-NTA spin columns is provided on page 45.

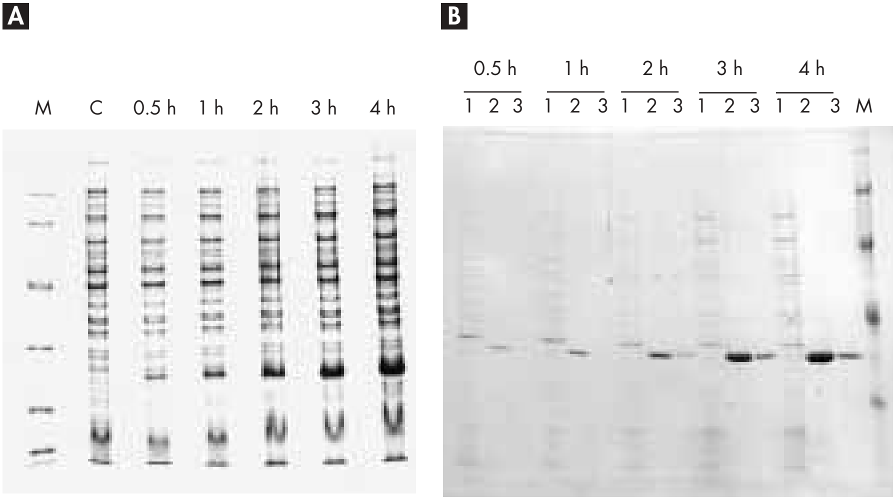

Figure 17. *Time course of expression using the QIAexpress System. Expression of 6xHis-tagged DHFR was induced with 1 mM IPTG. Aliquots were removed at the times indicated and purified on Ni-NTA Agarose under denaturing conditions. Proteins were visualized by Coomassie staining. Yields per liter culture were 2.8, 5.5, 12.3, 33.8, and 53.9 mg, respectively.* **A** *Crude cell lysate;* **B** *purification with Ni-NTA.* ***1****: flow-through,* ***2 & 3****: first and second eluates;* ***M****: markers;* ***C****: noninduced control.*

Specific considerations

Low expression levels

Low-level expression can occur because the protein is toxic or unstable, or because the expression construct is not maintained in the cells during growth. In some cases, the 5' end of the inserted DNA sequence may encode elements that interfere with transcription or translation (e.g., masking of the Shine-Dalgarno sequence by stem-loop structures resulting from inverted repeats); in these instances the sequence being expressed should be checked and modified if necessary. Modifications of growth media and different host strains may also have an effect on expression.

Toxic gene products

The expression of heterologous proteins can slow the growth of the host cells. High transcription rates lead to slow growth, and this in turn is compounded by metabolic demands imposed by translation of the recombinant protein. Gene products that affect the host cell's growth rate at low concentrations are considered to be toxic; examples include membrane proteins or proteins that interact with DNA or interfere with electron transport.

Some proteins are only mildly toxic and appear to be expressed poorly after the cells have been kept in culture media or on plates for several days. The maintenance of cells can be complicated by the fact that the desired cells are likely to be outgrown by cells that harbor mutants of the plasmid and do not express the toxic protein. Constructs encoding proteins

that are more toxic generally lower the transformation efficiencies of the host cells as compared to the original parent vector, and plasmids in transformants that do arise often have deletions and mutations.

To reduce the effects of protein toxicity on cell growth prior to induction, the level of basal transcription that occurs in the absence of induction ("leakiness") should be repressed as much as possible, and the number of generations before induction should be kept to a minimum. Problems related to the loss of plasmids can sometimes be overcome by growing the cells in the presence of high levels of ampicillin (200 µg/ml) or carbenicillin (50 µg/ml). For instable expression constructs, overnight starter cultures should be avoided. Colonies from a fresh plate should be inoculated into a small starter culture and grown for 2–3 hours, until mid-log phase. This starter culture should then be diluted 20–50-fold in prewarmed medium and grown to an OD_{600} of approximately 0.5 before induction.

For very toxic proteins, we recommend using the pQE-80L series of expression vectors in the M15[pREP4] host strain. This combination of two repressor modules results in highly efficient suppression of recombinant protein expression prior to induction and gives the best chance of successful expression of toxic proteins.

Hydrophobic regions

Recombinant proteins with hydrophobic regions often have a toxic effect on host cells, most likely due to the association of the protein with or incorporation into vital membrane systems. Sequences encoding signal peptides or transmembrane domains, unless of specific interest, should be removed from the DNA inserts before they are cloned. However, it is also possible to express proteins containing signal peptides that target the protein molecules into the periplasmic space, albeit at a lower rate (see also "Secretion", page 23). Lowering the growth temperature to 25°C before induction is recommended. Examples of 6xHis-tagged, transmembrane proteins with membrane-spanning domains that have been expressed to significant levels in *E. coli* have also been reported (Waeber et al. 1993).

Unstable proteins

Some proteins, particularly those that are smaller than 10 kDa, are not stable in *E. coli*, and may be degraded rapidly by proteases. This may be overcome by:

- Reducing the growth temperature to 30°C
- Inducing for a shorter period of time
- Using a host strain deficient in one or more proteases (e.g. $OmpT^-$, Lon^-)
- Expressing short proteins and peptides as a fusion with DHFR (plasmids pQE-16 and pQE-40)
- Including glycerol in the purification buffers

If the protein is degraded during the purification process, it may be necessary to use one or more protease inhibitor, such as PMSF, leupeptin, or aprotinin (Wingfield 1995a) and to work at 4°C all times.

High expression levels, insoluble proteins, and inclusion bodies

Eukaryotic proteins expressed intracellularly in *E. coli* are frequently sequestered into insoluble inclusion bodies. The intermolecular association of hydrophobic domains during folding is believed to play a role in the formation of inclusion bodies. For proteins with cysteine residues, improper formation of disulphide bonds in the reducing environment of the *E. coli* cytoplasm may also contribute to incorrect folding and formation of inclusion bodies.

An advantage of the QIA*express* purification system is that 6xHis-tagged proteins in insoluble inclusion bodies can be easily solubilized with denaturants such as 6 M Gu·HCl or 8 M urea or with a variety of detergents and be purified on Ni-NTA matrices. Proteins purified under denaturing conditions can then be refolded if necessary before use (Wingfield and Palmer 1995). Some general tips for the protein refolding are given on page 106 in the section "Protein refolding recommendations".

While it is possible to obtain functionally active protein using this approach, many researchers have found that their recoveries are poor when a refolding step is included in the purification protocol. An alternative approach is to adjust expression conditions such that smaller amounts of recombinant protein are produced in a soluble, native form. Even if inclusion bodies are formed, some 6xHis-tagged protein will often remain soluble in the cytoplasm, from which they can be purified on Ni-NTA matrices under native conditions (see protocols beginning on page 82). If higher levels of soluble protein are desired, a reduction in growth temperature following induction may be helpful. Growth temperature often directly affects both expression levels and protein solubility, and lower temperatures will reduce expression levels leading to a higher amount of soluble protein. Alternatively, the culture can also be grown to a higher cell density before induction and the expression period can be kept to a minimum. The IPTG concentration can be reduced from 1 mM to 0.005 mM, which would reduce the expression level by 90–95%. Furthermore, it may be sufficient to change the host strain used, since certain strains tolerate some proteins better than others and allow higher levels of expression before forming inclusion bodies. Finally, many proteins require metal cofactors in order to remain soluble, and the addition of metal salts to the culture media may be helpful. If the metal requirements of the protein are not known, a number of different supplements should be tested. Note that some divalent cations may interfere with protein binding to Ni-NTA.

Other Expression Systems

The choice of expression system is dictated by the specific applications for which the protein is being produced. Bacterial expression is recommended for obtaining maximum expression rates and if post-translational modifications of the recombinant protein are not required for protein function. After initial studies that can be carried out in bacterial expression systems, expression in another system such as insect, yeast, or mammalian cells may be required in order to obtain higher protein activity, functional disulfide bridges, or eukaryotic-specific post-translational modifications.

Baculovirus

Baculovirus vectors used to heterologously express proteins in insect cells have also become widely used (Luckow 1991; Miller 1988; O'Reilly et al. 1994). These systems are based on the ability of the baculovirus to infect and multiply in cultured insect cells. The most widely used virus is the *Autographa californica* nuclear polyhedrosis virus (AcNPV), a lytic virus that infects lepidopterans. A foreign gene is cloned into a plasmid transfer vector and then cotransfected along with double-stranded baculovirus DNA into insect cells. Homologous recombination of the plasmid and insert DNA with viral DNA in vivo leads to the insertion of the sequence encoding the recombinant protein into the viral genome. Insect cells which produce the recombinant proteins also recognize most vertebrate protein-targeting sequences and can thus express a wide variety of proteins, including cytoplasmic, nuclear, membrane-spanning, and secreted proteins. Many post-translational modifications typically encountered in vertebrate cells, such as phosphorylation, glycosylation, precursor processing, and targeting are also carried out in insect cells. Recombinant proteins can thus be either produced within the cells or secreted into the culture medium.

The pQE-TriSystem vector is a suitable shuttle vector for recombinant protein expression in insect cells. It carries two segments of the AcNPV genomic DNA (ORF 603 and ORF 1629) bracketing the p10 promoter, a multiple cloning site and transcription-terminator sequences (see vector information page 56, and vector map below). Due to the presence of the AcNPV sequences, recombinant baculovirus can be generated by homologous recombination at the *polh* locus. Generally, recombinant baculoviruses are prepared by cotransfection of the shuttle vector and linearized baculovirus genomic DNA into insect cells such as Sf9 and Sf21 cells. The pQE-TriSystem vector is compatible with the genomic baculovirus preparations derived from *Autographa californica* polyhedrosis virus (AcNPV) commercially available as BacVector®-3000 Triple Cut Virus DNA (Novagen, cat. no. 70078-3), BaculoGold™ linearized baculovirus DNA (BD Pharmingen, cat. no. 21100D) and BacPAK6 DNA (Clontech, cat. no. 6144-1). Optimized protocols for cotransfection, virus amplification and plaque assay for virus titer determination can be taken from the protocols of the different suppliers.

Several established cell lines are highly susceptible to AcNPV virus infection. The two most frequently used insect cell lines are Sf9 and Sf21 established from ovarian tissues of *Spodoptera frugiperda* larvae. Expression levels of recombinant protein vary between 0.05–50% of the total insect-cell protein content. For optimal protein production the multiplicity of infection (MOI, number of applied viruses per cell) should be between 0.2 and 10. Researchers should test different MOI to empirically determine optimum levels of protein production. With increasing MOI, protein production and subsequent cell lysis will be accelerated. Use of a MOI higher than one means that a considerable portion of the virus population may contain deletion mutants or non-recombinant viruses leading to reduced expression levels of the correct recombinant protein.

Heterologous gene-expression levels in the baculovirus system can vary by approximately 1000-fold depending on the intrinsic nature of the gene and the encoded protein. Optimization of the gene construct will generally influence protein production by only 2–5 fold. Two main factors should be considered. Firstly, the 5′-untranslated region between the promoter and the ATG start codon should be reduced in length, and secondly, additional ATG codons upstream of the gene should be avoided, since translation will start at the first ATG initiation codon downstream of the promoter. In the pQE-TriSystem vector the distance between the promoter and ATG start codon in front of the multiple cloning site is optimized for recombinant gene expression.

A protocol for the purifiction of 6xHis-tagged proteins from baculovirus-infected insect cells appears on page 88 of this handbook.

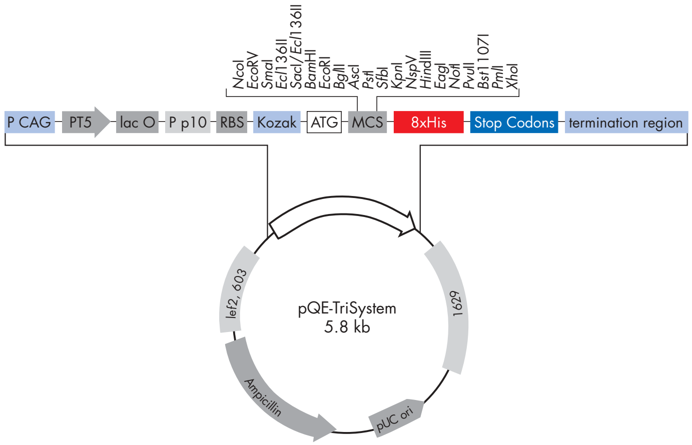

Figure 18. *pQE-TriSystem vector for parallel protein expression using a single construct in* E. coli*, insect, and mammlian cells.* ***PT5****: T5 promoter,* ***lac O****: lac operator,* ***RBS****: ribosome binding site,* ***ATG****: start codon,* ***8xHis****: His tag sequence,* ***MCS****: multiple cloning site,* ***Stop Codons****: stop codons in all three reading frames,* ***Ampicillin****: ampicillin resistance gene,* ***P CAG****: CMV/actin/globin promoter,* ***P p10****: p10 promoter,* ***Kozak****: Kozak consensus sequence,* ***termination region****: transcription terminator region,* ***lef2, 603/1629****: flanking baculovirus sequences to permit generation of recombinant baculoviruses,* ***pUC****: pUC origin of replication.*

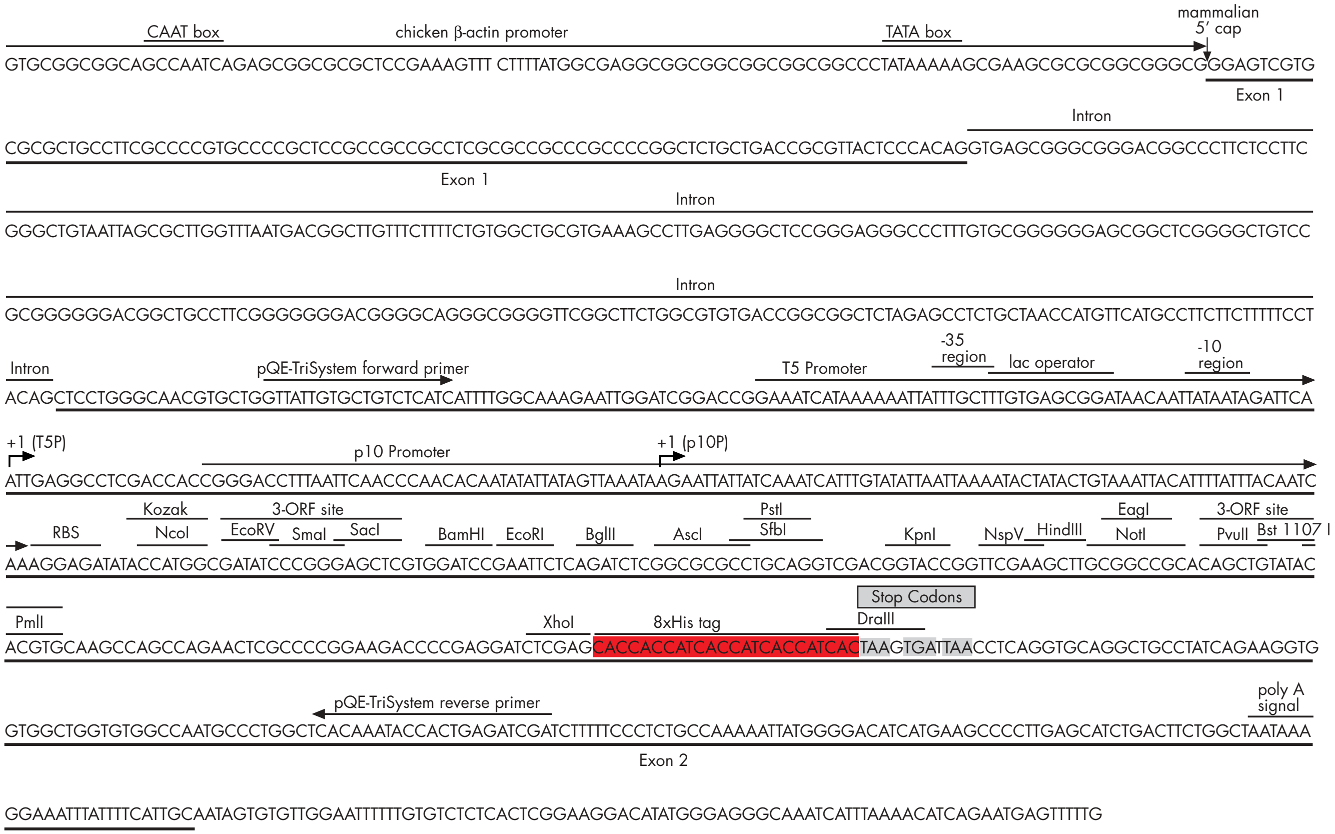

***Figure 19.** pQE-TriSystem promoter region overview and sequencing primer annealing positions*

Mammalian cells

Even though expression levels are usually low, mammalian cells are often the best host for the expression of recombinant vertebrate proteins because they produce the same post-translational modifications and recognize the same signals for synthesis, processing, and secretion utilized in the organism from which the sequence was originally derived. A wide variety of mammalian expression vectors are currently in use. In general they contain an efficient promoter element for high-level transcription initiation, mRNA processing signals such as mRNA cleavage and polyadenylation sequences, selectable markers to select mammalian cells that have stably integrated the DNA into their genome, and plasmid sequences that permit the propagation of the vectors in bacterial hosts.

pQE-TriSystem is a suitable vector for transient recombinant protein expression in mammalian cells. The combination of the CMV immediate-early enhancer fused to the chicken β-actin promoter results in a strong promoter for constitutive heterologous gene expression. The polyadenylation signals of the mRNA transcript are encoded by the downstream rabbit-globin terminator. The presence of the Kozak consensus sequence including the ATG start codon facilitates efficient translation initiation. pQE-TriSystem vector can be introduced into the cell by traditional transfection techniques such as calcium phosphate or liposome mediated transfection, and electroporation. QIAGEN offers three transfection reagents based on the latest advances in transfection technology; the non-liposomal–lipid—based Effectene™ Transfection Reagent, and the proven activated-dendrimer–based Superfect® Transfection Reagent and Polyfect® Transfection Reagent. A protocol for purification of 6xHis tagged proteins from mammalian cells is given on page 86.

Yeast

Expression of recombinant proteins in yeast combines the advantages of providing most eukaryotic post translational modifications such as phosphorylation, glycosylation, and targeting, with expression levels ranging up to several milligrams per liter of culture (up to 30% of the expression of total yeast protein). A comprehensive review is provided by Romanos and coworkers (1992).

The most widely used expression vectors are *E. coli*/yeast shuttle plasmids (Baldarini and Cesareni 1985; Clare et al. 1991) that are mitotically stabilized by autonomously replicating sequences (ARS/CEN region, 2µ locus) or by integration into the yeast genome. The episomal expression constructs are introduced into the cells by transformation into competent cells (Gietz et al. 1992) or by electroporation. Various strategies can be employed to induce expression of the recombinant proteins encoded by these constructs, but the specific method chosen depends on the physiological characteristics of the yeast strain being used. Commonly used strategies are exemplified by the galactose-inducible expression systems in *Saccharomyces cerevisae* and methanol-driven induction in the methylotropic yeast, *Pichia pastoris*. Intracellular 6xHis-tagged proteins to be purified must be released from the cells by disrupting cell walls by enzymatic, chemical, or mechanical

means (Sambrook et al. 1989; Ausubel et al. 1995; Guthrie and Fink 1991). Purification can be simplified by including an export signal in-frame with the 6xHis tag. Proteins will be targeted into the medium and can then be purified by Ni-NTA affinity chromatography.

Yeast cells may acidify the culture medium and both the cells and the medium may contain certain compounds that influence binding of 6xHis tags to Ni-NTA matrices. Methods used to remove these ingredients and purify the 6xHis-tagged proteins are described in the section "Purification of 6xHis-tagged proteins produced in other expression systems" on page 76.

Expression procedures

Protocol 6. Determination of target protein solubility

Materials

LB medium

Kanamycin stock solution

Ampicillin stock solution

Lysis buffer for purification under native conditions (page 114)

1x SDS-PAGE sample buffer

2x SDS-PAGE sample buffer

Buffer compositions are provided in the appendix on page 111.

Culture growth

1. **Inoculate 10 ml LB medium containing 100 µg/ml ampicillin and 25 µg/ml kanamycin in a 50 ml flask. Grow the cultures overnight at 37°C with shaking.**

   Kanamycin should be omitted when using the *cis*-repressed pQE-80L series of vectors.
2. **Inoculate 50 ml of prewarmed media (with antibiotics) with 2.5 ml of the overnight cultures and grow at 37°C, with vigorous shaking (~300 rpm), until the OD_{600} is 0.5–0.7 (approximately 30–60 min).**
3. **Take a 1 ml sample immediately before induction (noninduced control), pellet cells, and resuspend in 50 µl 1x SDS-PAGE sample buffer. Freeze the sample at –20°C until needed for SDS-PAGE.**
4. **Induce expression by adding IPTG to a final concentration of 1 mM.**
5. **Grow the cultures for an additional 4–5 hours. Collect a second 1 ml sample (induced control), pellet cells and resuspend in 100 µl 1x SDS-PAGE sample buffer. Freeze until use.**
6. **Harvest the cells by centrifugation at 4000 x *g* for 20 min.**

Protein extraction

1. **Resuspend cell pellet in 5 ml of lysis buffer for native purification.**
2. **Freeze sample in dry ice/ethanol, and thaw in cold water.**

   Alternatively, add lysozyme to 1 mg/ml and incubate on ice for 30 min.
3. **Sonicate 6 x 10 s with 10 s pauses at 200–300 W. Keep lysate on ice at all times.**

   Use a sonicator with a microtip probe.
4. **Centrifuge lysate at 10,000 x *g* at 4°C for 20–30 min. Decant the supernatant (crude extract A, soluble protein) and save on ice.**
5. **Resuspend the pellet in 5 ml lysis buffer. This is a suspension of the insoluble matter (crude extract B, insoluble protein).**

SDS-PAGE analysis

1. **Add 5 µl of 2x SDS-PAGE sample buffer to 5 µl of crude extracts A & B.**
2. **Heat these samples, along with the frozen noninduced and induced cell samples at 95°C for 5 min.**
3. **Microcentrifuge at 15,000 x *g* for 1 min.**
4. **Load 20 µl of the noninduced and induced cell samples, and all of the extract samples on a 12% SDS-PAGE gel. Run the gel according to standard procedures.**

Interpretation of results

If the protein of interest is in the insoluble matter (extract B), ensure that the cells are completely lysed. If the protein is still insoluble, try extracting the pellet with 0.25% Tween 20, 0.1 mM EGTA a few times; often the protein is not truly insoluble but just associated with the membrane fragments in the cell pellet. If the protein is truly insoluble under these conditions, purify under denaturing conditions.

Protocol 7. Growth of standard *E.coli* expression cultures (100 ml)

1. **Inoculate 10 ml of culture medium containing both ampicillin (100 µg/ml) and kanamycin (25 µg/ml) in a 50 ml flask. Grow the cultures at 37°C overnight.**

   Kanamycin should be omitted when using the *cis*-repressed pQE-80L series of vectors.

2. **Inoculate 100 ml of prewarmed media (with antibiotics) with 5 ml of the overnight cultures and grow at 37°C with vigorous shaking until an OD_{600} of 0.6 is reached (30–60 min).**

3. **Take a 1 ml sample immediately before induction.**

   This sample is the noninduced control; pellet cells and resuspend them in 50 µl 5x SDS-PAGE sample buffer. Freeze until SDS-PAGE analysis.

4. **Induce expression by adding IPTG to a final concentration of 1 mM.**

5. **Incubate the cultures for an additional 4–5 h. Collect a second 1 ml sample.**

   This sample is the induced control; pellet cells and resuspend them in 100 µl 5x SDS-PAGE sample buffer. Freeze and store the sample at –20°C until SDS-PAGE analysis.

6. **Harvest the cells by centrifugation at 4000 x *g* for 20 min.**

7. **Freeze the cells in dry ice–ethanol or liquid nitrogen, or store cell pellet overnight at –20°C.**

Protocol 8. *E.coli* culture growth for preparative purification (1 liter)

1. **Inoculate 20 ml of LB broth containing 100 µg/ml ampicillin and 25 µg/ml kanamycin. Grow at 37°C overnight with vigorous shaking.**

   Kanamycin should be omitted when using the *cis*-repressed pQE-80L series of vectors.

2. **Inoculate a 1 liter culture (LB, 100 µg/ml ampicillin, 25 µg/ml kanamycin) 1:50 with the noninduced overnight culture. Grow at 37°C with vigorous shaking until an OD_{600} of 0.6 is reached.**

3. **Take a 1 ml sample immediately before induction.**

   This sample is the noninduced control; pellet cells and resuspend in 50 µl 5x SDS-PAGE sample buffer. Freeze until use.

4. **Induce expression by adding IPTG to a final concentration of 1 mM.**

5. **Incubate the culture for an additional 4–5 h. Collect a second 1 ml sample.**

   This is the induced control; pellet cells in a microcentrifuge and resuspend in 100 µl 5x PAGE sample buffer. Freeze until use.

6. **Harvest the cells by centrifugation at 4000 x *g* for 20 min.**

7. **Freeze the cells in dry ice–ethanol or liquid nitrogen, or store cell pellet overnight at –20°C.**

Troubleshooting: expression

	Comments and suggestions
**No or low expression**	
Protein is poorly expressed.	Check that the protein is not found in the insoluble fraction.<br>Review “Specific considerations” beginning on page 51.
Culture conditions for expression are incorrect.	Use the same culture conditions and host cells to check the expression of DHFR encoded by a control plasmid (pQE-40).
Coding sequence is ligated into the incorrect reading frame.	Sequence the ligated junctions.
Protein is secreted.	Remove all signal sequences from the coding region.
Protein is rapidly degraded.	Perform a time course to check the kinetics of growth and induction. If the protein is small (<10 kDa), consider adding an N-terminal carrier protein such as DHFR.<br>If degradation occurs after cell lysis, consider adding protease inhibitors.<br>Keep all samples and solutions at 4°C.
**Inclusion bodies are formed**	
Expression level is too high (protein cannot fold correctly).	Reduce expression levels by modifying growth and induction conditions (see “High expression levels, insoluble proteins, and inclusion bodies” on page 53).
Protein is insoluble.	Check both soluble and insoluble fractions for protein (see Protocol 6, on page 59).<br>Try to solubilize protein with denaturants or detergents.<br>See “Specific considerations” beginning on page 51 for ways to enhance solubility of proteins by altering the growth and induction conditions.<br>**Note**: It may not be necessary to use denaturing conditions for purification if the protein of interest is insoluble or has formed inclusion bodies. Check the levels of soluble protein remaining in the cytoplasm. This may be purified with Ni-NTA matrices.
Protein is highly toxic.	Use *E. coli* M15 [pREP4] in combination with one of the pQE-80L series of expression vectors.

Purification

Optimal expression of recombinant proteins in various expression systems including *E. coli* can be easily achieved when the vectors and host cells are carefully chosen, and the growth conditions are properly controlled. Culture conditions and the induction of expression have profound effects on the way the recombinant protein is produced, and in this context directly influence the strategies employed for protein purification. It is therefore advisable to empirically establish optimal conditions with small-scale cultures before purification on a larger scale is attempted. Recombinant proteins expressed in *E. coli* can be produced in a soluble form, but in many cases, especially at high expression levels, they aggregate and form insoluble inclusion bodies. The formation of inclusion bodies is influenced by the nature of the protein, by the host cell, and by the level of expression resulting from the vector choice and the growth and induction conditions. Inclusion bodies invariably limit the utility of standard purification procedures which rely on the protein's native soluble form. Purification of 6xHis-tagged proteins by Ni-NTA affinity chromatography, however, can be performed under native or denaturing conditions and is not affected by problems arising from protein insolubility. Most proteins in inclusion bodies are simply solubilized with detergents or denaturants such as 8 M urea or 6 M GuHCl before the purification steps are initiated.

The basic principles pertaining to the Ni-NTA affinity purification procedure under native or denaturing conditions are outlined in the flowchart (Figure 20); more details appear in the following sections.

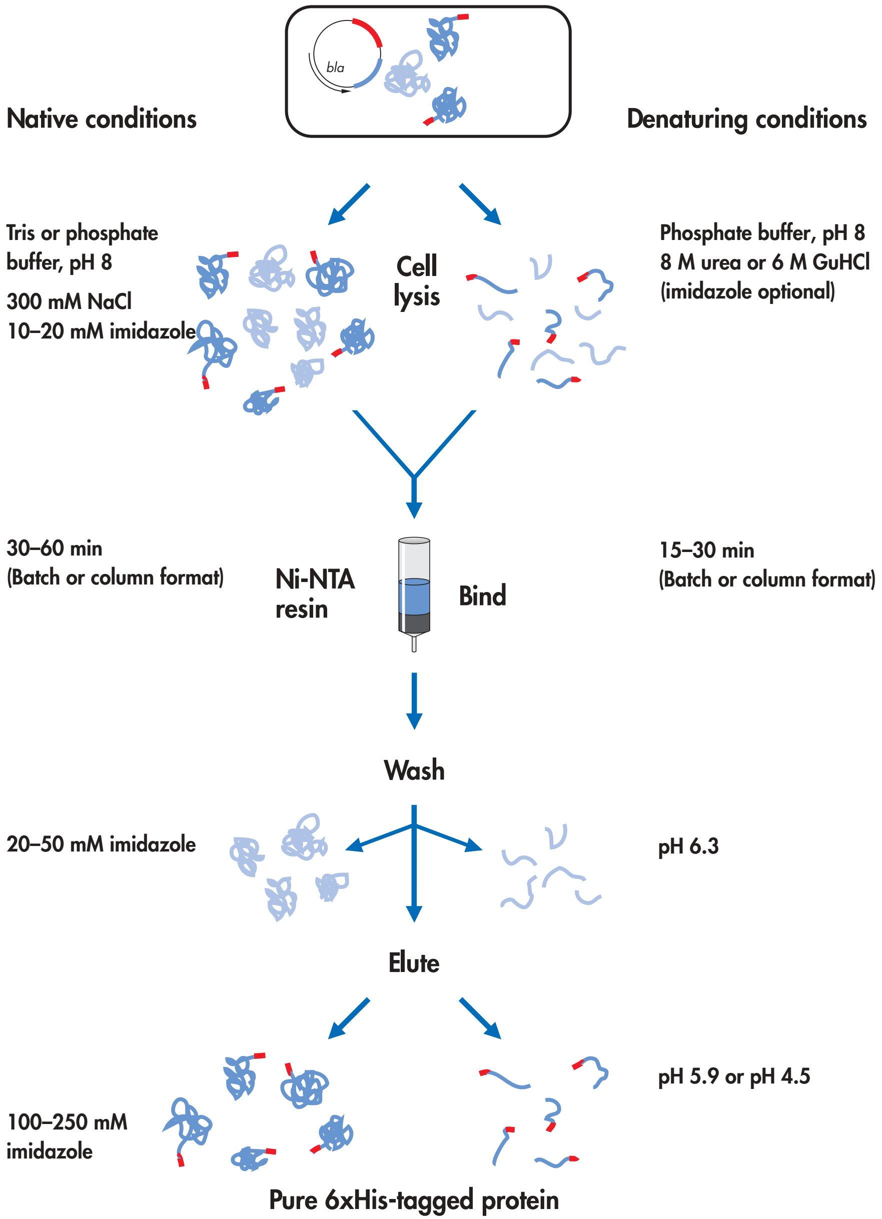

Figure 20. *Purification of 6xHis-tagged proteins using the QIAexpress System.*

Basic principles

Culture size

Optimal purification is dependent on a number of factors, including the amount of 6xHis-tagged protein required and expression level.

The amount of culture required depends on the level at which the protein is expressed, which must be determined empirically for each expression experiment. If the protein is not expressed efficiently, bacteria from a large culture volume must be lysed in a given volume of lysis buffer corresponding to a high "concentration factor". The concentration factor is defined as the ratio of the culture size to the amount of lysis buffer used. Examples of the relationship between expression levels and recommended culture volumes and concentration factors are provided in Table 3.

For example, if a protein that is expressed at 0.1 mg/liter and a miniature batch purification procedure will be used under denaturing conditions (see Protocol 19, page 92), a 100 ml culture should be concentrated 100-fold by resuspending the pellet in 1 ml buffer.

Under native conditions, it is more difficult to predict the amount of soluble protein present in the lysate. Generally, 50- to 100-fold concentration is recommended to purify significant amounts of 6xHis-tagged protein.

Table 3. Determination of cell culture volume requirements

Concentration of 6xHis-tagged protein	Expression level	Culture volume	Amount of 6xHis-tagged protein	Concentration factor*
**Denaturing conditions**				
50 mg/liter	40%	3 ml	150 µg	3x
10 mg/liter	8%	10 ml	100 µg	10x
2 mg/liter	1.6%	25 ml	50 µg	25x
0.5 mg/liter	0.4%	50 ml	25 µg	50x
0.1 mg/liter	0.08%	100 ml	10 µg	100x
**Native conditions**				
>1 mg/liter	>1%	50 ml	>50 µg	50x
<1 mg/liter	<1%	100 ml	<100 µg	100x

**After lysis in 1 ml.*

Reducing nonspecific binding

Since there is a higher potential for binding background contaminants under native conditions than under denaturing conditions, low concentrations of imidazole in the lysis and wash buffers (10–20 mM) are recommended. The imidazole ring is part of the structure of histidine (Figure 21). The imidazole rings in the histidine residues of the 6xHis tag bind to the nickel ions immobilized by the NTA groups on the matrix. Imidazole itself can also bind to the nickel ions and disrupt the binding of dispersed histidine residues in nontagged background proteins. At low imidazole concentrations, nonspecific, low-affinity binding of background proteins is prevented, while 6xHis-tagged proteins still bind strongly to the Ni-NTA matrix. Therefore, adding imidazole to the lysis buffer leads to greater purity in fewer steps. For most proteins, up to 20 mM imidazole can be used without affecting the yield. If the tagged protein does not bind under these conditions, the amount of imidazole should be reduced to 1–5 mM.

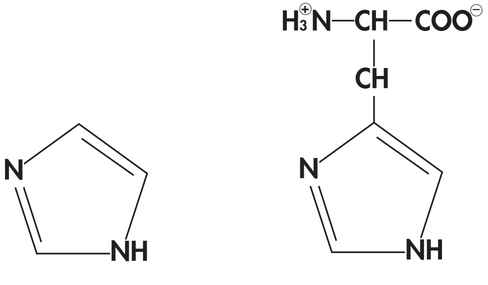

Figure 21. Chemical structures of histidine and imidazole.

Binding of tagged proteins to Ni-NTA resin is not conformation-dependent and is not affected by most detergents and denaturants (Table 4, page 74). The stability of the 6xHis–Ni-NTA interaction in the presence of low levels of β-ME (up to 20 mM) in the lysis buffer can be used to prevent the copurification of host proteins that may have formed disulfide bonds with the protein of interest during cell lysis. Detergents such as Triton X-100 and Tween 20 (up to 2%), or high salt concentrations (up to 2 M NaCl) (Table 4, page 74), also have no effect on binding, and may reduce nonspecific binding to the matrix due to nonspecific hydrophobic or ionic interactions. Nucleic acids that might associate with certain DNA and RNA-binding proteins are also removed without affecting the recovery of the 6xHis-tagged protein.

Protein solubility and intracellular location

Since the interaction between Ni-NTA and the 6xHis tag of the recombinant protein does not depend on tertiary structure, proteins can be purified either under native or denaturing conditions. To set up the best purification strategy, it is important to determine whether

the protein is soluble in the cytoplasm or located in cytoplasmic inclusion bodies (see Protocol 6, page 59). Many proteins form inclusion bodies when they are expressed at high levels in bacteria, while others are tolerated well by the cell and remain in the cytoplasm in their native configuration. Proteins that contain appropriate leader peptide sequences may be secreted into the periplasmic space, but this depends on the host cell and on the nature of both the leader peptide and the recombinant protein.

Note: When leader peptides are used, the 6xHis tag cannot be located at the N-terminus, because it would be removed together with the leader sequence following secretion.

Purification under native or denaturing conditions

The decision whether to purify 6xHis-tagged proteins under native or denaturing conditions depends on protein location and solubility, the accessibility of the 6xHis tag, the downstream application, and whether biological activity must be retained. Furthermore, if efficient renaturing procedures are available, denaturing purification and subsequent refolding may be considered.

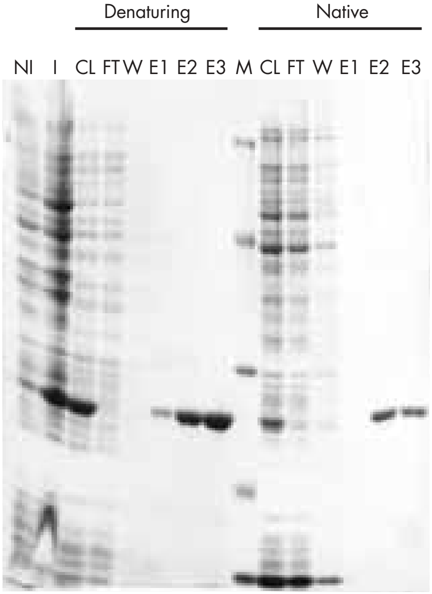

Figure 22. *Denaturing and native purification of heterologously expressed DHFR.* ***NI****: noninduced cells;* ***I****: cells induced with IPTG;* ***CL****: cleared lysate;* ***FT****: flow-through;* ***W****: wash;* ***E1–E3****: eluates. 10% of DHFR is present in soluble form, which can be effectively purified under native conditions.*

Purification

Purification under native conditions

If purification under native conditions is preferred or necessary, the 6xHis-tagged protein must be soluble. However, even when most of the protein is present in inclusion bodies, there is generally some soluble material that can be purified in its native form (Figure 22). Purification of tagged proteins under native conditions can be exploited to copurify associated proteins such as enzyme subunits and binding proteins present in the expressing cells (Le Grice and Grueninger-Leitch, 1990; Garner et al. 1992; Flachmann and Kühlbrandt 1996), added to the lysate prior to purification, or added to the Ni-NTA matrix after the 6xHis-tagged protein is bound.

The potential for unrelated, nontagged proteins to interact with the Ni-NTA resin is usually higher under native than under denaturing conditions. This is reflected in the larger number of proteins that appear in the first wash (Figure 23). Nonspecific binding can be reduced by including a low concentration of imidazole (10–20 mM) in the lysis and wash buffers.

In rare cases the 6xHis tag is hidden by the tertiary structure of the native protein, so that soluble proteins require denaturation before they can be purified on Ni-NTA. As a control, a parallel purification under denaturing conditions should always be carried out. If purification is only possible under denaturing conditions, and this is undesirable, the problem with inaccessible tags can generally be solved by moving the tag to the opposite terminus of the protein.

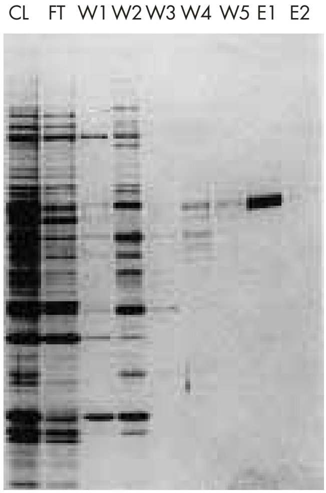

Figure 23. *Purification under native conditions. Human serum response factor (SRF) was expressed from a vaccinia virus vector in HeLa cells and purified using Ni-NTA Agarose with the indicated imidazole concentrations in the wash and elution steps. Proteins were visualized by Coomassie staining.* ***CL****: cell lysate;* ***FT****: flow-through;* ***W1****: 0.8-mM wash;* ***W2 & W3****: 8-mM wash;* ***W4 & W5****: 40-mM wash;* ***E1 & E2****: 80-mM elution. (Reproduced by kind permission of H. Stunnenberg, EMBL, Heidelberg, Germany)*

It is difficult to provide a general protocol for the purification of 6xHis-tagged proteins under native conditions because there is significant variation in protein structure, which can interfere with binding. However there are some general suggestions that may aid in optimizing the native purification procedure:

- Cells can be lysed by sonication or homogenization after treatment with lysozyme. To prevent protein degradation, cells and protein solutions should be kept at 0–4°C at all times; the addition of protease inhibitors may also be necessary.
- Low concentrations of imidazole in the lysis and wash buffers minimize nonspecific binding and reduce the amount of contaminating proteins.
- All buffers should have sufficient ionic strength to prevent nonspecific interactions between proteins and the Ni-NTA matrix. The minimum salt concentration during binding and washing steps should be 300 mM NaCl. The maximal concentration is 2 M NaCl (Table 4, page 74).

Secretion efficiency for proteins containing an export signal sequence can be determined for secreted proteins by purification of the periplasmic extract after osmotic shock (see Protocol 11, page 81).

Purification under denaturing conditions

High levels of expression of recombinant proteins in a variety of expression systems can lead to the formation of insoluble aggregates; in *E. coli*, these are known as inclusion bodies. Strong denaturants such as 6 M GuHCl or 8 M urea completely solubilize inclusion bodies, and 6xHis-tagged proteins. Other denaturants or detergents can be used as well, but the choice of these reagents and the specific concentrations needed must be established empirically. Under denaturing conditions, the 6xHis tag on a protein will be fully exposed so that binding to the Ni-NTA matrix will improve, and the efficiency of the purification procedure will be maximized by reducing the potential for nonspecific binding.

6xHis-tagged proteins purified under denaturing conditions can be used directly, or may have to be renatured and refolded. Protein renaturation and refolding can be carried out on the Ni-NTA column itself prior to elution (Holzinger et al. 1996), or in solution (Wingfield et al. 1995a); additional suggestions are included in this manual (see "Protein refolding recommendations", page 106).

Batch or column purification

Proteins may be purified on Ni-NTA resins in either a batch or a column procedure. The batch procedure entails binding the protein to the Ni-NTA resin in solution and then packing the protein–resin complex into a column for the washing and elution steps. This strategy promotes efficient binding of the 6xHis-tagged protein especially when the 6xHis tag is not fully accessible or when the protein in the lysate is present at a very low concentration.

In the column procedure, the Ni-NTA resin is first packed into the column and equilibrated with the lysis buffer. The cell lysate is then slowly applied to the column. Washing and elution steps are identical in the batch and column procedure.

Protein binding

Proteins containing one or more 6xHis affinity tags, located at either the amino and/or carboxyl terminus of the protein, can bind to the Ni-NTA groups on the matrix with an affinity far greater than that of antibody–antigen or enzyme–substrate interactions. Binding of the 6xHis tag does not depend on the three-dimensional structure of the protein. Even when the tag is not completely accessible it will bind as long as more than two histidine residues are available to interact with the nickel ion; in general, the smaller the number of accessible histidine residues, the weaker the binding will be. Untagged proteins that have histidine residues in close proximity on their surface will also bind to Ni-NTA, but in most cases this interaction will be much weaker than the binding of the 6xHis tag. Any host proteins that bind nonspecifically to the NTA resin itself can be easily washed away under relatively stringent conditions that do not affect the binding of 6xHis-tagged proteins.

Binding can be carried out in a batch or column mode (see "Batch or column purification", page 69). If the concentration of 6xHis-tagged proteins is low, or if they are expressed at low levels, or secreted into the media, the proteins should be bound to Ni-NTA in a batch procedure, and under conditions in which background proteins do not compete for the binding sites, i.e. at a slightly reduced pH or in the presence of low imidazole concentrations (10–20 mM). At low expression levels under native conditions, binding can be optimized for every protein by adjusting the imidazole concentration and/or pH of the lysis buffer. If high levels of background proteins are still present, equilibrating the Ni-NTA matrix with lysis buffer containing 10–20 mM imidazole prior to binding is recommended. The matrix is thus "shielded", and nonspecific binding of proteins that weakly interact is significantly reduced.

Wash

Endogenous proteins with histidine residues that interact with the Ni-NTA groups can be washed out of the matrix with stringent conditions achieved by lowering the pH to 6.3 or by adding imidazole at a 10–50 mM concentration. In bacterial expression systems, the recombinant proteins are usually expressed at high levels, and the level of copurifying contaminant proteins is relatively low. Therefore it generally is not necessary to wash the bound 6xHis-tagged protein under very stringent conditions. In lysates derived from eukaryotic expression systems the relative abundance of proteins that may contain neighboring histidines is higher; the resulting background problem becomes more critical especially when nondenaturing procedures are employed. In these instances it becomes necessary to increase the stringency of the wash steps considerably. This can be performed most effectively by gradually decreasing the pH of the wash buffer or by slowly increasing the concentration of imidazole in defined steps; step-gradients are preferable because they are much more effective than linear gradients when metal affinity chromatography methods are employed. The optimal pH and/or imidazole concentrations for the washes will vary slightly for each protein and must be determined empirically.

Protein elution

The histidine residues in the 6xHis tag have a pK_a of approximately 6.0 and will become protonated if the pH is reduced (pH 4.5–5.3). Under these conditions the 6xHis-tagged protein can no longer bind to the nickel ions and will dissociate from the Ni-NTA resin. Similarly, if the imidazole concentration is increased to 100–250 mM, the 6xHis-tagged proteins will also dissociate because they can no longer compete for binding sites on the Ni-NTA resin.

Elution conditions are highly reproducible, but must be determined for each 6xHis-tagged protein being purified. Monomers generally elute at approximately pH 5.9, whereas aggregates and proteins that contain more than one 6xHis tag elute at approximately pH 4.5.

Reagents such as EDTA or EGTA chelate the nickel ions and remove them from the NTA groups. This causes the 6xHis-tagged protein to elute as a protein–metal complex. NTA resins that have lost their nickel ions become white in color and must be recharged if they are to be reused.

Whereas all elution methods (imidazole, pH, and EDTA) are equally effective, imidazole is mildest and is recommended under native conditions, when the protein would be damaged by a reduction in pH, or when the presence of metal ions in the eluate may have an adverse effect on the purified protein.

Removal of the 6xHis tag

Although it is rarely necessary to remove the short 6xHis affinity tag from the recombinant protein after purification, there are some applications, such as structural analysis by X-ray crystallography or NMR, where the removal of the tag may be desirable. In order to cleave off an N-terminal tag the protease cleavage site must be inserted between the coding sequences of the 6xHis tag and the N-terminus of the protein. Factor Xa Protease recognizes the amino acid sequence Ile-Glu-Gly-Arg and cleaves the peptide bond C-terminal of the arginine residue. The expression vector pQE-30 Xa encodes a Factor Xa Protease recognition site between the N-terminal 6xHis-tag sequence and the multiple cloning site. If the gene of interest is cloned blunt ended at the 5´-end using the *Stu*I restriction site of the vector, Factor Xa Protease cleavage of the purified recombinant protein results in a protein product without any vector-derived amino acids at the N-terminus. After protease digestion, the protein of interest can be repurified in two steps. Xa Removal Resin binds Factor Xa Protease in a batch procedure, and is removed by centrifugation. Subsequently, cleaved 6xHis-tag peptides and undigested 6xHis-tagged protein can be captured by Ni-NTA affinity chromatography (see Protocol 20, page 93).

Tags can also be removed exoproteolytically using the TAGZyme System (Schäfer et al. 2002b). This complete system provides high-purity proteins free of vector-encoded amino acids for use in applications that demand the use of recombinant reagents, an absence of non-specific cleavage, and a complete removal of all contaminants. For more information on the TAGZyme System, call QIAGEN Technical Services or your local distributor.

Specific considerations

Contaminating proteins

Proteins that contain neighboring histidines are not common in bacteria, but are quite abundant in eukaryotic cells. These proteins bind to the Ni-NTA resin much more weakly than proteins with a 6xHis tag, and can thus be easily washed away, even when they are much more abundant than the tagged protein (Janknecht et al. 1991). The addition of low concentrations of imidazole in the binding and wash buffers is very effective in preventing contaminating proteins from binding to the Ni-NTA matrix in the first place. This is especially important when purifying 6xHis-tagged proteins under native conditions.

The addition of 20 mM β-mercaptoethanol (β-ME) to the lysis buffer will reduce disulfide bonds that may have formed between contaminating proteins and the 6xHis-tagged protein. Dithiothreitol (DTT) should not be used (see Table 4, page 74).

Proteins that copurify because they are linked to the 6xHis-tagged protein, proteins that associate nonspecifically with the tagged protein, and nucleic acids that associate with the tagged protein can appear as contaminants in the eluate. All of these contaminants can be easily removed by washing the resin under more stringent conditions, by adding low concentrations of a detergent (0.1–1% Triton X-100 or 0.3% Sarkosyl), by increasing the salt concentration to up to 2 M NaCl, or by including ethanol or glycerol to reduce hydrophobic interactions. The optimal amounts of any of these reagents should be determined empirically for each purification protocol.

In some rare cases nontagged, cellular proteins may bind to the carbohydrate resin of Ni-NTA Agarose or Ni-NTA Superflow itself.

Truncated 6xHis-tagged proteins are common contaminants that are copurified by Ni-NTA affinity chromatography. These contaminants result from internal initiation of translation (C-terminally tagged proteins), from premature translation termination (N-terminally tagged proteins), or from protein degradation during protein expression or purification. This can be assessed by monitoring the size of 6xHis-tagged proteins using 6xHis detection reagents (Anti·His Antibodies or Ni-NTA Conjugates) in western blots. Changing the location of the tag to the amino or carboxyl terminus may be necessary to eliminate or minimize the appearance of these truncated forms. Protease inhibitors may also have to be added before and during the lysis steps to minimize protein degradation.

It is preferable to choose column size and total binding capacity to approximately match the amount of 6xHis-tagged protein to be purified (Schmitt et al. 1993a). 6xHis-tagged proteins have a higher affinity for the Ni-NTA resin than background proteins. Consequently very few nontagged proteins will be retained on the resin if nearly all available binding sites are occupied by the tagged protein. If too much Ni-NTA matrix is used, other proteins may nonspecifically bind to unoccupied sites and elute as contaminants.

Limitations

Ni-NTA matrices should not be exposed to high concentration of strong reducing agents such as DTT or DTE; these reagents reduce the nickel ions and thereby prevent them from binding 6xHis-tagged proteins. Ni-NTA resins that have been reduced turn brown in color. In most situations, β-mercaptoethanol can be used at concentrations up to 20 mM.

EDTA, EGTA, or any other strong chelating agents bind nickel ions and strip them from the NTA matrices. NTA resins become white in the absence of nickel ions.

Use any reducing or chelating agent with care, and if in doubt, test it on a small amount of Ni-NTA resin. High concentrations of buffer components containing strong electron-donating groups or amino acids such as arginine, glutamine, glycine, or histidine in the lysate should also be avoided.

Cells should be lysed without the use of strong chelating agents such as EDTA, strong reducing agents such as DTT, or ionic detergents such as SDS. Although there are instances in which small amounts of these reagents have been used successfully, we do not recommend their use.

For more detailed information, refer to Table 4, page 74.

Table 4. Compatibility of reagents with Ni-NTA matrices

Reagent	Effect	Comments
**Buffer reagents**		
Tris, HEPES, MOPS	• Buffers with secondary or tertiary amines will reduce nickel ions	• Up to 100 mM has been used successfully in some cases<br>• Sodium phosphate or phosphate-citrate buffer is recommended
**Chelating reagents**		
EDTA, EGTA	• Strip nickel ions from resin	• Up to 1 mM has been used successfully in some cases, but care must be taken
**Sulfhydril reagents**		
β-mercaptoethanol	• Prevents disulfide cross-linkages<br>• Can reduce nickel ions at higher concentration	• Up to 20 mM
DTT, DTE	• Low concentrations will reduce nickel ions	• A maximum of 1 mM may be used, but β-mercaptoethanol is recommended
**Detergents**		
Nonionic detergents (Triton, Tween, NP-40, etc.)	• Removes background proteins and nucleic acids	• Up to 2% can be used
Cationic detergents		• Up to 1% can be used
CHAPS		• Up to 1% can be used
Anionic detergents (SDS, sarkosyl)		• Not recommended, but up to 0.3% has been used successfully in some cases
**Denaturants**	• Solubilize proteins	
GuHCl		• Up to 6 M
Urea		• Up to 8 M
**Amino acids**		
Glycine		• Not recommended
Glutamine		• Not recommended

Table continued overleaf

Purification

Table 4. Continued

Reagent	Effect	Comments
Arginine		• Not recommended
Histidine	• Binds to Ni-NTA and competes with histidine residues in the 6xHis tag	• Can be used at low concentrations (20 mM) to inhibit non-specific binding and, at higher concentrations (>100 mM), to elute the 6xHis-tagged protein from the Ni-NTA matrix
**Other additives**		
NaCl	• Prevents ionic interactions	• Up to 2 M can be used, at least 300mM should be used
$MgCl_2$		• Up to 4 M
$CaCl_2$		• Up to 5 mM
Glycerol	• Prevents hydrophobic interaction between proteins	• Up to 50%
Ethanol	• Prevents hydrophobic interactions between proteins	• Up to 20%
Imidazole	• Binds to Ni-NTA and competes with histidine residues in the 6xHis tag	• Can be used at low concentrations (20 mM) to inhibit non-specific binding and, at higher concentrations (>100 mM), to elute the 6xHis-tagged protein from the Ni-NTA matrix
Sodium bicarbonate		• Not recommended
Hemoglobin		• Not recommended
Ammonium		• Not recommended
Citrate		• Up to 60 mM has been used successfully

Purification of 6xHis-tagged proteins produced in other expression systems

Purification of 6xHis-tagged proteins expressed in mammalian cells

Purification of 6xHis-tagged recombinant proteins expressed intracellularly can pose problems. Some of the difficulties of using mammalian cells are that expression levels are typically lower than in bacterial systems and that often only small amounts of cell material are available.

- The total protein content in HeLa cells, for example, is only 3000 µg per 10^7 cells. With recombinant protein expression levels at 0.01 to 1% of total protein, the theoretical maximum protein yield is 0.3 to 30 µg per 10^7 cells.
- Cells can be lysed by sonication, by using freeze-thaw cycles, or by treatment with non-ionic detergents.
- Adjustment of the binding capacity of the Ni-NTA matrix used to the amount of solubilized 6xHis-tagged protein to be captured is crucial for optimal performance and reduced copurification of contaminants.

Ni-NTA Magnetic Agarose Beads are an ideal Ni-NTA matrix for micro-scale protein purification from 10^7–10^8 cells. The total binding capacity of the beads used (3 µg DHFR [24 kDa] binds per 10 µl magnetic bead suspension) can be easily adjusted to the amount of 6xHis-tagged protein expressed by 10^7–10^8 cells allowing efficient purification even from dilute solutions of recombinant protein. Furthermore, proteins can be eluted into very small elution volumes allowing detection of the purified proteins using Coomassie-stained SDS polyacrylamide gels (Wahle et al. 1999). A detailed protocol is described on pages 86–87 (Protocol 15). For large-scale purification from larger amounts of cells we recommend using Ni-NTA Agarose or Ni-NTA Superflow which bind 0.5–1 mg of a 24 kDa 6xHis tagged protein per 100 µl resin. Cell lysis can be performed by simply scaling up the micro-scale protocol.

In the hope of increasing the expression rates and of facilitating the purification of the protein of interest without having to resort to cell lysis, it is often attractive to exploit secretion of the 6xHis-tagged protein directly into the medium. Mammalian cell culture media are often supplemented with serum proteins which bind weakly to the Ni-NTA matrix and compete with the 6xHis-tagged protein for binding sites. Amino acids with electron-donating groups such as glutamine or histidine are commonly added to media and have a similar effect.

- First try to purify the 6xHis-tagged protein directly from your medium. Experiments with some widely used media (DMEM, DMEM+10% fetal calf serum, CHO-S-SFMII, and RPMI 1640) showed purification recovery rates of 6xHis-tagged thioredoxin in the range of 45–75% (Wahle et al. 1999).

- If the efficiency of 6xHis-tagged protein purification directly from the culture medium is not efficient enough, there are several ways to make the binding conditions more suitable.

We have obtained good results by adding 1/10 volume of a 10x buffer containing, for example, 500 mM NaH_2PO_4, pH 8.0, 1.5 M NaCl, 100 mM imidazole to the medium. This results in appropriate composition and pH (8.0) of the medium for binding 6xHis-tagged proteins. The pH as well as concentrations of NaH_2PO_4, imidazole, and NaCl are then similar to those in the lysis buffer recommended for purification under native conditions. In the example, NaCl is added to increase the final concentration by 150 mM because most media contain NaCl in physiological concentrations resulting in a final concentration of 300 mM. Variations in the concentrations of NaCl and imidazole have to be considered depending on the culture medium used and 6xHis-tagged protein to be purified.
Alternative methods such as dialysis of the medium against a buffer providing optimal binding conditions, size-exclusion chromatography, or ion-exchange chromatography can also be considered (Coligan et al. 1995; Deutscher 1990).

Purification of 6xHis-tagged proteins expressed in insect cells

If 6xHis-tagged proteins are expressed intracellularly without virus-mediated lysis of the cells, purification can be performed as described for mammalian systems (see "Purification of 6xHis-tagged proteins expressed in mammalian cells", page 76 and protocol 15, page 86).

Expression of 6xHis-tagged proteins secreted directly into the medium can pose some problems. Media used to culture insect cells usually have an acidic pH (6.0–6.5) or contain electron-donating groups that can prevent binding of the 6xHis-tagged protein to Ni-NTA. Amino acids such as glutamine, glycine, or histidine are present at significantly higher concentrations in media for growing insect cells than in media for growing mammalian cells and compete with the 6xHis-tagged protein for binding sites on Ni-NTA matrices. Grace's medium (Life Technologies), for example, contains approximately 10 mM glutamine, 10 mM glycine, and 15 mM histidine. Table 5 summarizes the results of experiments where we have analyzed recovery rates after purification directly from various media. 6xHis-tagged thioredoxin and 6xHis-tagged chloramphenicol acetyl transferase (CAT) were added to some widely used insect cell media and purified with Ni-NTA Agarose. Recovery rates were between 30 and 100%.

Table 5. Recovery rates of protein purification from insect cell media (as a percentage of the control)*

Medium	Recovery rate for 6xHis-CAT	Recovery rate for 6xHis-thioredoxin
High Five™ Serum-Free Medium	100	100
TMN-FH Serum-containing Medium	75	80
Grace's Serum-containing Medium	60	70
SF-900 Serum-Free Medium	100	95
TC-100 Serum-Free Medium	30	50
BaculoGold™ Serum-Free Medium	100	100

* *Control: Purification of CAT and thioredoxin from 50 mM NaH_2PO_4, 300 mM NaCl, 10 mM imidazole, pH 8.0*

- First try to purify the 6xHis-tagged protein directly from your media.
- If purification efficiency is not sufficient, several options for optimizing binding conditions can be tested as follows.

Dialysis of the medium against a buffer with the appropriate composition and pH (8.0) similar to the lysis buffer recommended for purification under native conditions usually restores optimal binding conditions. Note that depending on the media used a white precipitate (probably made up of insoluble salts) can occur, but normally the 6xHis-tagged protein remains in solution. This can be tested by either protein quantitation if using a protein-free medium or by monitoring the amount of 6xHis-tagged protein by western-blot analysis using the QIA*express* Detection System (RGS·His, Penta·His, or Tetra·His Antibodies). After centrifugation, 6xHis-tagged protein can be directly purified from the cleared supernatant.

Alternatively, the pH of the medium can be adjusted to 8.0 with a phosphate or Tris·Cl buffer, but again salt precipitation may occur.

Other methods such as size-exclusion chromatography or ion-exchange chromatography can also be considered (Coligan et al. 1995; Deutscher 1990).

Purification procedures

Protocol 9. Preparation of cleared *E. coli* lysates under native conditions

Materials

Cell pellet

Lysis buffer

Lysozyme

2x SDS-PAGE sample buffer

Buffer compositions are provided in the appendix on pages 111–114.

1. **Thaw the cell pellet for 15 min on ice and resuspend the cells in lysis buffer at 2–5 ml per gram wet weight.**

   The amount of cells required depends on the expression level of the 6xHis-tagged protein and the expression system used. The binding capacity of Ni-NTA resins is protein-dependent and normally lies between 5–10 mg/ml. For example, Ni-NTA Agarose or Ni-NTA Superflow has a binding capacity of 0.3 µmol/ml (8.0 mg/ml) for 6xHis-tagged DHFR (~26 kDa). **Refer to Table 3 "Determination of cell culture volume requirements" on page 65.**

   Lysis buffer contains 10 mM imidazole to minimize binding of untagged, contaminating proteins and increase purity with fewer wash steps. If the tagged protein does not bind under these conditions, the amount of imidazole should be reduced to 1–5 mM. With 6xHis-tagged proteins exhibiting high binding affinities, the imidazole concentration can be increased to 20 mM.

2. **Add lysozyme to 1 mg/ml and incubate on ice for 30 min.**
3. **Sonicate on ice using a sonicator equipped with a microtip.**

   Use six 10 s bursts at 200–300 W with a 10 s cooling period between each burst.

4. **(Optional) If the lysate is very viscous, add RNase A (10 µg/ml) and DNase I (5 µg/ml) and incubate on ice for 10–15 min.**

   Alternatively, draw the lysate through a narrow-gauge blunt-ended syringe needle several times.

5. **Centrifuge lysate at 10,000 x *g* for 20–30 min at 4°C to pellet the cellular debris. Save supernatant.**

   A certain proportion of the cellular protein, including the 6xHis-tagged protein, may remain insoluble and will be located in the pellet. For more complete recovery of the tagged protein, this material must be solubilized using denaturing conditions as described in Protocol 10 on page 80 before purification under denaturing conditions.

6. **Add 5 µl 2x SDS-PAGE sample buffer to 5 µl supernatant and store at –20°C for SDS-PAGE analysis.**
7. **Proceed to protocols for purification under native conditions beginning on page 81.**

Protocol 10. Preparation of cleared *E. coli* lysates under denaturing conditions

Materials

Cell pellet

2x SDS-PAGE sample buffer

Buffer B

Buffer compositions are provided in the appendix on pages 111–114.

1. **Thaw the cell pellet for 15 min on ice and resuspend in buffer B at 5 ml per gram wet weight.**

   Cells can be lysed in either 6 M GuHCl or 8 M urea. It is preferable to lyse the cells in the milder denaturant, urea, so that the cell lysate can be analyzed directly by SDS-PAGE. GuHCl is a more efficient solubilization and cell lysis reagent, however, and may be required to solubilize some proteins. Prior to SDS-PAGE analysis, samples containing guanidine must be treated as described in the appendix on page 115.

   The amount of cells required depends on the expression level of the 6xHis-tagged protein and the expression system used. The binding capacity of Ni-NTA resins is protein-dependent and normally lies between 5–10 mg/ml. For example, Ni-NTA Agarose or Ni-NTA Superflow has a binding capacity of 0.3 µmol/ml (8.0 mg/ml) for 6xHis-tagged DHFR (~26 kDa). **Refer to Table 3 "Determination of cell culture volume requirements" on page 65.**

2. **Stir cells for 15–60 min at room temperature or lyse them by gently vortexing, taking care to avoid foaming.**

   Lysis is complete when the solution becomes translucent.

3. **Centrifuge lysate at 10,000 x *g* for 20–30 min at room temperature to pellet the cellular debris.**

   Save supernatant (cleared lysate).

4. **Add 5 µl 2x SDS-PAGE sample buffer to 5 µl supernatant and store at –20°C for SDS-PAGE analysis.**

5. **Proceed to protocols for purification under denaturing conditions beginning on page 90.**

Protocol 11. Preparation of 6xHis-tagged periplasmic proteins from *E. coli*

Periplasmic proteins are proteins secreted into the periplasmic space located between the outer and inner membrane of *E. coli*. Proper secretion is possible only when the protein of interest has an N-terminal signal peptide which is cleaved following translocation. In order to purify proteins secreted into the periplasmic space using Ni-NTA technology, the 6xHis tag must be engineered to the C-terminus of the target protein. N-terminal 6xHis tags will be processed with the transit signal.

Materials

30 mM Tris·Cl, 20% sucrose, pH 8.0

500 mM EDTA

5 mM $MgSO_4$

Lysis buffer

Buffer compositions are provided in the appendix on pages 111–114.

1. **Grow and induce a 1 liter culture as described in Protocol 8, page 61.**
2. **Harvest the cells by centrifugation at 4,000 x *g* for 20 min. Resuspend pellet in 30 mM Tris·Cl, 20% sucrose, pH 8.0, at 80 ml per gram wet weight. Keep the cells on ice and add 500 mM EDTA dropwise to 1 mM. Incubate the cells on ice for 5–10 min with gentle agitation.**
3. **Centrifuge the cell suspension at 8000 x *g* for 20 min at 4°C, remove all the supernatant, and resuspend the pellet in the same volume of ice-cold 5 mM $MgSO_4$. Shake or stir for 10 min in an ice bath.**
4. **Centrifuge at 8000 x *g* for 20 min at 4°C.**

   The supernatant is the osmotic shock fluid containing periplasmic proteins.
5. **Dialyze supernatant extensively against lysis buffer before continuing with the purification.**

For purification under native conditions see below.

Protocols for purification under native conditions

Before purifying proteins under nondenaturing conditions, it is important to check that the protein is soluble (see Protocol 6, page 59). However, even if most of the protein is insoluble, it is often possible to purify traces of soluble material on Ni-NTA resin.

In the absence of strong denaturing agents such as urea, unstable proteins may be subject to degradation during cell harvest and lysis. It is best to work quickly and to keep the cells at 0–4°C at all times. Addition of PMSF or other protease inhibitors may be helpful on a case-by-case basis, but their potential effect on the recombinant protein must be taken into consideration.

Protocol 12. Batch purification of 6xHis-tagged proteins from *E. coli* under native conditions

The amount of cells required depends on the expression level of the 6xHis-tagged protein and the expression system used. The binding capacity of Ni-NTA resins is protein-dependent and normally lies between 5 and 10 mg/ml. For example, Ni-NTA Agarose or Ni-NTA Superflow has a binding capacity of 0.3 µmol/ml resin (8.0 mg/ml) for 6xHis-tagged DHFR (~26 kDa). The resins are supplied as 50% slurries.

For proteins that are expressed at high levels, (10–50 mg of 6xHis-tagged protein per liter of cell culture) a 10x concentrated cell lysate (resuspend the pellet from a 40 ml culture in 4 ml lysis buffer) can be used. Four ml of a 10x concentrated cell lysate in lysis buffer will contain approximately 0.4–2 mg of 6xHis-tagged protein. For much lower expression levels (1–5 mg/liter), 200 ml of cell culture should be used to obtain a 50x concentrated cell lysate (4 ml cell lysate = 0.2–1 mg of 6xHis-tagged protein). See also “Culture size”, page 65 for more information.

The lysis buffer contains 10 mM imidazole to minimize binding of untagged, contaminating proteins and increase purity with fewer wash steps. If the tagged protein does not bind under these conditions, the amount of imidazole should be reduced to 1–5 mM. With 6xHis-tagged proteins exhibiting high binding affinities, the imidazole concentration can be increased to 20 mM.

Materials

Cleared lysate from a 40–200 ml culture (see Protocol 9, page 79)

Ni-NTA matrix

Empty columns

Lysis buffer

Wash buffer

Elution buffer

Buffer compositions are provided in the appendix on page 114.

1. **Add 1 ml of the 50% Ni-NTA slurry to 4 ml cleared lysate and mix gently by shaking (200 rpm on a rotary shaker) at 4°C for 60 min.**

   The 10–20 mM imidazole in the lysis buffer suppresses the binding of nontagged contaminating proteins and leads to greater purity after fewer wash steps. If the tagged protein does not bind under these conditions, the amount of imidazole should be reduced to 1–5 mM.

2. **Load the lysate–Ni-NTA mixture into a column with the bottom outlet capped.**
3. **Remove bottom cap and collect the column flow-through.**

   Save flow-through for SDS-PAGE analysis.

4. **Wash twice with 4 ml wash buffer; collect wash fractions for SDS-PAGE analysis.**

5. **Elute the protein 4 times with 0.5 ml elution buffer. Collect the eluate in four tubes and analyze by SDS-PAGE.**

   The composition of the lysis, wash and elution buffers can be modified to suit the particular application, e.g. by adding 0.1% Tween, 5–10 mM β-ME, or 1 mM PMSF, or increasing NaCl or glycerol concentrations. For more information, see "Purification under native conditions", page 68.

Protocol 13. FPLC purification of 6xHis-tagged proteins from *E. coli* using Ni-NTA Superflow under native conditions

If larger amounts of protein are to be purified or if the purification will be performed using FPLC equipment, a Ni-NTA Superflow column is the method of choice (Figure 24). The physical stability of Ni-NTA Superflow makes it ideal for column chromatography at higher pressures and flow rates.

FPLC Purification on Ni-NTA Superflow

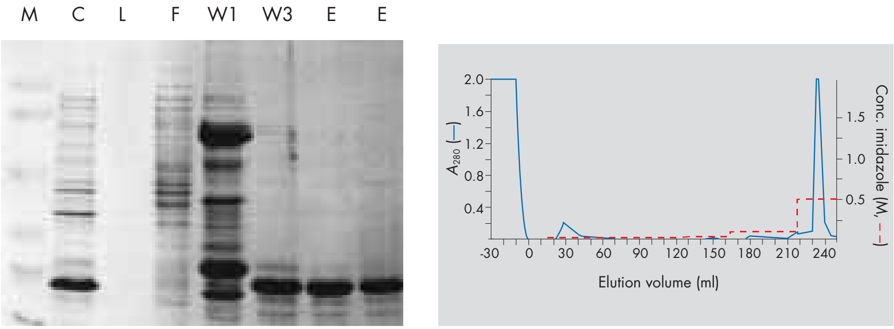

Figure 24. *FPLC purification on Ni-NTA Superflow. 6xHis-tagged phosphatase (~19 kDa) was purified from cleared lysate (1.2 liters) derived from 18 liters induced* E. coli *culture on 6 ml of Ni-NTA Superflow in a 1 cm column at 2 ml/min. Total yield was 38 mg.* ***Left****: Coomassie-stained SDS gel;* ***right****: elution profile.* ***M****: markers;* ***C****: induced cells;* ***L****: lysate;* ***F****: flow-through;* ***W1****: 20 mM wash;* ***W3****: 100 mM wash;* ***E****: eluates. (Data kindly provided by T. Schäfer, Institute for Biochemistry, University of Lübeck, Germany.)*

Materials

Cleared lysate from a 40–200 ml culture (see Protocol 9, page 79)

Ni-NTA Superflow

Lysis buffer

Wash buffer

Elution buffer

Chromatography column

Buffer compositions are provided in the appendix on page 114.

1. **Assemble the column according to the manufacturer's instructions. Remove the top adapter of the column and cap the bottom outlet.**

2. **Completely resuspend a 50% Ni-NTA Superflow slurry and pour the slurry into the column.**

   Avoid introducing air bubbles. Slowly pour the slurry down a thin glass rod inserted into the empty column. The column and bed size depends on the amount of 6xHis-tagged protein to be purified. Generally, the binding capacity of Ni-NTA Superflow is 5–10 mg protein per ml resin. Ni-NTA Superflow is supplied as a 50% slurry.

3. **Allow the resin to settle.**

   The packing procedure can be accelerated by allowing the buffer to flow through by uncapping the bottom outlet. If desired, a peristaltic pump may be used, but do not exceed flow rates of 2 ml/min.

   Do not allow resin to dry. If this should occur, resuspend resin in lysis buffer and repack the column.

   Before the bed has settled, more slurry may be added to increase bed volume.

4. **Insert top adapter and adjust to top of bed.**

   Do not trap any air bubbles. The column can now be connected to the system.

5. **Equilibrate column with 5 column volumes of lysis buffer**

   The flow rate should not exceed 2 ml/min.

   Monitor elution at 280 nm; the baseline should be stable after washing with 5 column volumes.

6. **Apply lysate to column and wash with lysis buffer until the A_{280} is stable.**

   Usually 5–10 column volumes are sufficient.
   Monitor pressure at this step. If the lysate is very viscous, the pressure may exceed the recommended value (10 bar). Reduce flow rate accordingly.
   Start with a flow rate of 0.5–1 ml/min. If the 6xHis-tagged protein does not bind, the flow rate should be reduced. The flow rate may however be increased for protein elution.
   Collect the flow-through for SDS-PAGE analysis.

7. **Wash with wash buffer until the A_{280} is stable.**

   Usually 5–10 column volumes are sufficient.
   Collect fractions for SDS-PAGE analysis.

8. **Elute the protein with elution buffer.**

   If desired, a step-gradient of elution buffer in wash buffer may be used to elute the protein. Five column volumes at each step are usually sufficient. The 6xHis-tagged protein usually elutes in the second and third column volume.

   Note: Imidazole absorbs at 280 nm, which should be considered when monitoring protein elution. If small amounts of 6xHis-tagged proteins are purified, elution peaks may be poorly visible.

Protocol 14. Protein minipreps of 6xHis-tagged proteins from *E. coli* under native conditions

Materials

Cell culture

Lysis, wash, and elution buffers

Ni-NTA matrix

Lysozyme

Buffer compositions are provided in the appendix on pages 111–114.

1. **Transfer 1 ml of bacterial culture to a microcentrifuge tube.**

   The amount of culture used depends on the protein expression level. One ml is sufficient if the protein is expressed at high rates (see Table 3, page 65). If lower expression rates are expected, larger volumes may be necessary.

   If a time course of expression is being performed, take 1 ml samples of a larger culture at 30 min intervals after induction, collect the cell pellets and store them at –20°C until all the samples are ready for processing.

2. **Harvest the cells by centrifugation for 1 min at 15,000 x *g* and discard supernatants.**

   If larger culture volumes are required, refill microcentrifuge tube and centrifuge. Repeat this step until all cells are harvested.

3. **Resuspend cells in 100 µl lysis buffer.**

   If 1 ml culture is used, the concentration factor is ten. This may not be sufficient for some proteins that are to be purified under native conditions (see Table 3, page 65).

4. **Add lysozyme to 1 mg/ml and incubate on ice for 30 min.**
5. **Lyse cells by gently vortexing, taking care to avoid frothing.**
6. **Centrifuge the lysate for 10 min at 15,000 x *g* to remove the cellular debris, and transfer the supernatant to a fresh tube.**
7. **Add 20 µl of a 50% slurry of Ni-NTA resin (10 µl resin has a capacity for 50–100 µg 6xHis-tagged protein) to each tube, and mix gently for 30 min at 4°C.**
8. **Centrifuge for 10 s at 1000 x *g* to pellet the resin, transfer 10 µl of the supernatant to a fresh tube, and discard the remaining supernatant.**

   Store the supernatant sample on ice. Supernatant samples will contain any proteins which have not bound to the resin.

9. **Wash the resin twice with 100 µl wash buffer.**

   Centrifuge for 10 s at 1000 x *g* between washes and carefully remove supernatant.

10. **Elute the protein 3 times with 20 µl elution buffer**

    Centrifuge for 10 s at 1000 x *g* between each elution step and carefully remove the supernatant to a fresh tube.

Protocol 15. Purification of 6xHis-tagged proteins from transfected mammalian cells under native conditions

The following procedure can be used as the starting point for developing a protocol for purification of 6xHis-tagged proteins expressed in mammalian cells. However, further optimization may be necessary. General guidelines for purification of 6xHis-tagged proteins using Ni-NTA Magnetic Agarose Beads are summarized in the *Ni-NTA Magnetic Agarose Beads Handbook*.

Recombinant proteins are often expressed in mammalian cells to allow eukaryotic post-translational processing. Some of the difficulties of using mammalian cells are that expression levels are typically lower than in bacterial systems and that often only small amounts of cell material are available. The total protein content in HeLa cells, for example, is only 3000 µg per 10^7 cells. With recombinant protein expression levels at 0.01 to 1% of total protein, the theoretical maximum protein yield is 0.3 to 30 µg per 10^7 cells.

Ni-NTA Magnetic Agarose Beads are an ideal Ni-NTA matrix for small-scale purification of 6xHis-tagged proteins expressed in mammalian cells (Wahle et al. 1999). They allow approximate adjustment of the binding capacity (3 µg DHFR [24 kDa] per 10 µl magnetic bead suspension) to the amount of 6xHis-tagged protein to be captured. This feature is crucial for optimal performance. In addition, the small elution volumes used provide high 6xHis-tagged protein concentrations, even with very small numbers of cells.

Materials

Cell pellet

Ni-NTA Magnetic Agarose Beads

Magnetic separator (QIAGEN 12-Tube Magnet, Cat. No. 36912)

PBS, lysis buffer, wash buffer, and elution buffer

Buffer compositions are provided in the appendix on pages 114–115.

Purification

1. **Wash the transfected cells with phosphate-buffered saline (PBS) and collect them by centrifugation for 5 min at 1000 x *g*.**
2. **Resuspend the cells in lysis buffer supplemented with 0.05% Tween® 20 using 500 µl lysis buffer per 10^7 cells.**

   The lysis buffer should always contain imidazole. For most 6xHis-tagged proteins, up to 20 mM imidazole can be used without affecting the binding properties. However, if the tagged protein does not bind under these conditions, the amount of imidazole should be reduced to 5–10 mM.

   If higher concentrations of non-ionic detergent are required to solubilize the 6xHis-tagged protein, use up to 1% detergent in the lysis, wash, and elution buffers. Compatible detergents are Tween 20, Triton® X-100, Igepal® CA-630, and CHAPS.

3. **Lyse the cells by sonication on ice.**

   Use six 15 s bursts at 75 W with a 10 s cooling period between each burst. Use a sonicator equipped with a microtip.

   Optional:

   A Lyse cells by three consecutive freeze/thaw cycles with freezing on dry ice and thawing at room temperature.

   B With 0.5–1% non-ionic detergent in the lysis buffer for solubilization of a hydrophobic 6xHis-tagged protein, it is sufficient to incubate on an end-over-end shaker for 10 min at 4°C. Additional sonication or freeze/thaw cycles are not necessary.

4. **Centrifuge the lysate at 10,000 x *g* for 10 min at 4°C to pellet cellular debris and DNA. Save the supernatant.**

   The supernatant should contain the 6xHis-tagged protein.

5. **Resuspend the Ni-NTA Magnetic Agarose Beads by vortexing for 2 s and then immediately add 10 µl of the 5% Ni-NTA Magnetic Agarose Bead suspension to 500 µl of the cleared lysate containing the 6xHis-tagged protein.**

   Note: Care is necessary to ensure that constant amounts of beads are pipetted. The beads tend to settle out if the suspension is not agitated regularly.

   10 µl magnetic-bead suspension has a binding capacity of 3 µg 6xHis-tagged DHFR (24 kDa). Use of volumes less than 10 µl is not recommended due to the associated handling problems. (Smaller volumes are difficult to pipet and may lead to uneven distribution of beads and reduced reproducibility.)

6. **Mix the suspension gently on an end-over-end shaker for 1–2 h at 4°C.**

7. **Place the tube on the QIAGEN 12-Tube Magnet for 1 min and remove the supernatant from the separated beads using a pipet.**

   To collect suspension droplets from the tube caps, it is helpful to briefly centrifuge the tubes before placing them on the 12-Tube Magnet.

8. **Remove the tube from the magnet, add 1 ml of wash buffer, mix the suspension, place the tube on the 12-Tube Magnet for 1 min, and remove wash buffer from the separated beads using a pipet.**

9. **Repeat step 8 two or three times.**

   After the final washing step, residual buffer should be removed completely.

10. **Add 50 µl of elution buffer, mix the suspension, incubate the tube for 1 min, place on the 12-Tube Magnet for 1 min, and collect the eluate using a pipet.**

    To collect suspension droplets from the tube caps, it is helpful to briefly centrifuge the tubes before placing them on the 12-Tube Magnet.

    If a more concentrated protein solution is required, elute in two aliquots of 25 µl each.

Protocol 16. Purification of 6xHis-tagged proteins from baculovirus-infected insect cells under native conditions

The following procedure can be used as a starting point for developing a protocol for purification of 6xHis-tagged proteins expressed intracellularly in insect cells. However, further optimization may be necessary. General guidelines for purification of 6xHis-tagged proteins using Ni-NTA Agarose or Superflow are summarized on pages 66–72.

Although expression rates are normally higher in insect cells than in mammalian cells, there are some difficulties connected with using baculovirus-infected insect cells for expression. Expressed-protein levels are typically lower than those obtained in bacterial systems, and in general, smaller amounts of cell material are available. The estimated total protein content in insect cells is approximately 20 mg per 10^7 cells. With recombinant protein expression levels ranging between 0.05 % and 50% the theoretical maximum protein yield is 10 µg – 10 mg per 10^7 cells.

Ni-NTA Agarose and Ni-NTA Superflow are ideal matrices for purification of 6xHis-tagged proteins expressed in baculovirus-infected insect cells. Use of an amount of matrix (binding capacity 500–1000 µg protein per 100 µl settled matrix) appropriate for the amount of 6xHis-tagged protein to be captured is crucial for optimal performance of the purification procedure.

Lysis buffer contains 1 % Igepal® CA-630 (Nonidet P40) for lysis of the cells and 10 mM imidazole to minimize binding of non-tagged, contaminating proteins, and to increase purity with fewer wash steps.

Materials

Cell pellet

Ni-NTA matrix

Empty columns

PBS, lysis buffer, wash buffer and elution buffer

Buffer compositions are provided in the appendix on pages 114–115

1. **Wash the transfected cells with phosphate buffered saline (PBS) and collect them by centrifugation for 5 min at 1000 x *g*.**
2. **Lyse the cells in lysis buffer supplemented with 1% Igepal CA-630 using 4 ml lysis buffer per 1–2 x 10^7 cells. Incubate for 10 min on ice.**

   The lysis buffer should always contain imidazole. For most 6xHis-tagged proteins, up to 20 mM imidazole can be used without affecting the binding properties. However, if the tagged protein does not bind under these conditions, the concentration of imidazole should be reduced to 5–10 mM.

3. **Centrifuge the lysate at 10,000 x *g* for 10 min at 4°C to pellet cellular debris and DNA. Save the cleared lysate (supernatant).**

   The supernatant should contain the 6xHis-tagged protein.

4. **Add 200 µl 50% Ni-NTA slurry per 4 ml of the cleared lysate and mix gently by shaking (200 rpm on a rotary shaker) at 4°C for 1–2 h.**

   Equilibrate the Ni-NTA with PBS before adding to the lysate.

5. **Load the lysate-Ni-NTA mixture into a column in which the outlet is capped.**

6. **Remove the outlet cap and collect the column flow-through fraction.**

   Save the flow-through fraction for SDS-PAGE analysis.

7. **Wash twice with 800 µl wash buffer, collecting wash fractions.**

   Save wash fractions for SDS-PAGE analysis.

8. **Elute the protein 4 times with 100 µl elution buffer. Collect the eluates in four tubes and analyze by SDS-PAGE.**

   The composition of the lysis, wash and elution buffers can be modified to suit the particular application, e.g. by adding 5-10 mM β-ME, or 1 mM PMSF, or increasing NaCl concentrations. For more information, see "Purification under native conditions", page 68.

Protocol 17. Batch purification of 6xHis-tagged proteins from *E. coli* under denaturing conditions

Materials

Cleared lysate (see Protocol 10, page 80) from 20–200 ml culture

Ni-NTA resin

Empty columns

Buffers A–E

Buffer compositions are provided in the appendix on page 113.

1. **Add 1 ml of the 50% Ni-NTA slurry to 4 ml lysate and mix gently by shaking (e.g., 200 rpm on a rotary shaker) for 15–60 min at room temperature.**

   The amount of lysate required depends on the expression level of the 6xHis-tagged protein and the expression system used. The binding capacity of Ni-NTA resins is protein-dependent and normally lies between 5–10 mg/ml. For example, Ni-NTA Agarose or Ni-NTA Superflow has a binding capacity of 0.3 µmol/ml (8.0 mg/ml) for 6xHis-tagged DHFR (~26 kDa).

   For proteins that are expressed at very high levels (50–100 mg of 6xHis-tagged protein per liter of cell culture), a 5x concentrated cell lysate (resuspend the pellet from a 20 ml culture in 4 ml buffer B) can be used. 4 ml of a 5x concentrated cell lysate in buffer B will contain approximately 1–2 mg of 6xHis-tagged protein. For much lower expression levels (1–5 mg/liter), 200 ml of cell culture should be used for a 50x concentrated cell lysate (4 ml cell lysate = 0.2–1 mg of 6xHis-tagged protein).

   See also "Culture size" on page 65 for more information.

2. **Load lysate–resin mixture carefully into an empty column with the bottom cap still attached.**

3. **Remove the bottom cap and collect the flow-through.**

   Collect flow-through for SDS-PAGE analysis.

4. **Wash twice with 4 ml buffer C.**

   Keep wash fractions for SDS-PAGE analysis.

5. **Elute the recombinant protein 4 times with 0.5 ml buffer D, followed by 4 times with 0.5 ml buffer E. Collect fractions and analyze by SDS-PAGE.**

   Monomers generally elute in buffer D, while multimers, aggregates, and proteins with two 6xHis tags will generally elute in buffer E.

Protocol 18. FPLC purification of 6xHis-tagged proteins using Ni-NTA Superflow under denaturing conditions

If larger amounts of protein are to be purified or if the purification will be performed using FPLC equipment, a Ni-NTA Superflow column is the method of choice. The physical stability of Ni-NTA Superflow makes it ideal for column chromatography at higher pressures and flow rates.

Materials

Ni-NTA Superflow

Buffers A–E

Chromatography column

1. **Assemble the column according to the manufacturer's instructions. Remove the top adapter of the column and cap the bottom outlet.**
2. **Thoroughly resuspend a 50% Ni-NTA Superflow slurry and pour the slurry into the column.**

   Avoid introducing air bubbles. Slowly pour the slurry down a thin glass rod inserted into the empty column.

   The column and bed size depends on the amount of 6xHis-tagged protein to be purified. Generally, the binding capacity of Ni-NTA Superflow is 5–10 mg protein per ml resin.
3. **Allow the resin to settle.**

   The packing procedure can be accelerated by allowing the buffer to flow through by uncapping the bottom outlet. If desired, a peristaltic pump may be used, but a flow rate of 2 ml/min should not be exceeded.

   Do not allow resin to dry. If this should occur, resuspend resin in buffer B and repack the column.

   Before the bed has settled, more slurry may be added to increase bed volume.
4. **Insert top adapter and adjust to top of bed.**

   Do not trap any air bubbles. The column can now be connected to the system.
5. **Equilibrate column with 5 column volumes of buffer B.**

   Monitor elution at 280 nm; the baseline should be stable after washing with 5 column volumes.
6. **Apply lysate to column and wash with buffer B until the A_{280} is below 0.01.**

   Usually 5–10 column volumes are sufficient.

   Begin with a flow rate of 1 ml/min. Monitor pressure at this step. If the lysate is very viscous, the pressure may exceed the recommended value (10 bar). If necessary reduce flow rate.

   Collect the flow-through for SDS-PAGE analysis.

7. **Wash with buffer C until the A_{280} is below 0.01.**

   Usually 5–10 column volumes are sufficient. The buffer C wash removes proteins that bind nonspecifically to the resin. Collect the wash fractions for SDS-PAGE analysis.

8. **Elute protein with buffer D or buffer E.**

   If elution is incomplete with buffer D, buffer E should be used. Monomers usually elute in buffer D, whereas multimers, aggregates, and proteins with two 6xHis tags will generally elute in buffer E. Proteins usually elute in the second and third column volume.

Protocol 19. 6xHis-tagged protein minipreps under denaturing conditions

1. **Transfer 1 ml bacterial culture to a microcentrifuge tube.**

   The amount of culture used depends on the protein expression level. 1 ml is sufficient if the protein is expressed at high rates (see Table 3, page 65). If lower expression rates are expected, larger volumes may be necessary.

   If a time course of expression is being performed, take 1 ml samples of a larger culture at 30 min intervals after induction, collect the cell pellets and store them at –20°C until all the samples are ready for processing.

2. **Harvest the cells by centrifugation for 1 min at 15,000 x *g* and discard supernatants.**

   If larger culture volumes are required, refill microcentrifuge tube and centrifuge. Repeat this step until all cells are harvested.

3. **Resuspend cells in 200 µl buffer B. Lyse cells by gently vortexing, taking care to avoid frothing.**

   The solution should become translucent when lysis is complete. Most proteins are soluble in buffer B. If the solution does not become translucent, lyse cells with buffer A.

4. **Centrifuge the lysate for 10 min at 15,000 x *g* to remove the cellular debris, and transfer the supernatant to a fresh tube.**

5. **Add 50 µl of a 50% slurry of Ni-NTA resin (25 µl resin has a capacity for 125–250 µg 6xHis-tagged protein) to each tube, and mix gently for 30 min at room temperature.**

6. **Centrifuge 10 sec at 15,000 x *g* to pellet the resin, transfer 10 µl of the supernatant to a fresh tube, and discard the remaining supernatant. Store the supernatant samples on ice.**

   The supernatant samples will contain any proteins which have not bound to the resin.

7. **Wash the resin twice with 250 µl of buffer C.**

   Centrifuge for 10 sec at 15,000 x *g* between each wash step and carefully remove the supernatant.

8. **Elute the protein 3 times with 25 µl buffer E.**

   Centrifuge for 10 sec at 15,000 x *g* between each elution step and carefully remove the supernatant to a fresh tube.

Protocol 20. Factor Xa Protease treatment of fusion proteins containing a Factor Xa Protease recognition sequence

Treatment of fusion proteins containing a Factor Xa Protease recognition sequence consists of three steps: a) Factor Xa Protease Cleavage, b) removal of Factor Xa Protease, and c) cleanup of the digested protein. Protocols for all three steps can be found below.

Protocol 20 a. Factor Xa Protease cleavage

Factor Xa Protease is a site-specific endoprotease that preferentially cleaves the C-terminal peptide bond of the recognition sequence Ile-Glu-Gly-Arg. Factor Xa Protease consists of two polypeptides linked by a disulfide bond. The optimal cleavage conditions must be determined individually for each protein to be cleaved. Accessibility of the cleavage site, the adjacent amino acid sequence, and the degree of protein aggregation all affect the cleavage efficiency. Optimization of Factor Xa Protease concentration, temperature (4° to 37°C), and incubation time (2 to 16 h) is recommended. Bear in mind that excess Factor Xa Protease may result in nonspecific proteolysis at secondary sites. Therefore, optimal enzyme specificity is achieved using the lowest amount of protease necessary to achieve complete cleavage.

Optimization of the cleavage conditions should be performed in small-scale reactions using the following protocol as a starting point. We recommend using 20 mM Tris·Cl, pH 6.5; 50 mM NaCl; 1 mM $CaCl_2$ as the reaction buffer.

Materials

Fusion protein containing a Factor Xa Protease recognition sequence

Factor Xa Protease

1x reaction buffer (20 mM Tris·Cl, pH 6.5; 50 mM NaCl; 1 mM $CaCl_2$)

5x SDS-PAGE sample buffer

Buffer compositions are provided in the appendix on pages 111–115.

1. **Prepare four solutions each containing 10 µg of the protein to be cleaved, in 1x reaction buffer. The solutions should have a protein concentration of at least 0.25 µg/µl.**

   Since Factor Xa Protease is sensitive to various buffer constituents we recommend that the protein to be cleaved is prepared in 1x reaction buffer before cleavage. If your individual protein requires other specific buffering conditions, please see the important notes below for the compatibility of some commonly used buffer components with Factor Xa Protease. We also recommend changing the buffer system if you have purified your protein by Ni-NTA affinity chromatography under native conditions, because Factor Xa Protease activity is sensitive to phosphate buffers as well as to high imidazole and NaCl concentrations.

2. **Prepare three serial dilutions of Factor Xa Protease in 1x reaction buffer with concentrations of 1.0, 0.2 and 0.05 Units per µl.**

   It is important to mix the dilutions completely before use to avoid localized differences in enzyme concentration. Diluted protease should be used immediately after preparation.

3. **Add 1 µl of each Factor Xa Protease dilution to one solution of the protein to be cleaved, and adjust the reaction volume to 40 µl using 1x reaction buffer. Adjust the volume of the fourth protein solution to 40 µl using 1x reaction buffer. This sample will serve as a negative control to monitor the progress of cleavage.**

Component	Volume/reaction	Final concentration
10 µg fusion protein	variable	0.25 µg/µl
Factor Xa Protease dilutions	1 µl	1.0, 0.2, and 0.05 U/40 µl
1x reaction buffer	Adjust to 40 µl	

4. **Incubate the reactions at room temperature (15–25°C). Take an 8 µl aliquot from each reaction after 3, 6, 9 and 16 h. Add 2 µl 5x SDS-PAGE sample buffer to each aliquot and mix thoroughly.**

   It is important to mix the aliquots with SDS-PAGE sample buffer immediately to completely quench Factor Xa Protease activity.

5. **Analyze the efficiency of cleavage in each sample by SDS-PAGE.**

   Since the cleaved-off peptide may be very small, the bands corresponding to cleaved and uncleaved protein will run very close together. We therefore recommend using a gradient gel that will give good resolution in the size-range of the protein being analyzed.

Important notes for optimization of cleavage

Protease concentration: Excess Factor Xa Protease may result in nonspecific proteolysis at secondary sites. Optimal enzyme specificity is achieved using the lowest amount of protease necessary to achieve complete cleavage.

Concentration of protein to be cleaved: Factor Xa Protease activity is sensitive to the concentration of protein to be cleaved. A minimum of 10 µg protein per 40 µl reaction (0.25 µg/µl) is recommended.

Incubation temperature: Factor Xa Protease activity increases with increasing incubation temperature from 4°C to 37°C. However, it should be taken into account that reduced incubation temperatures can minimize the accessibility of secondary cleavage sites.

pH: Factor Xa Protease activity decreases with increasing pH from pH 6.5 to pH 9.0. Therefore, we recommend a pH of 6.5 for the reaction buffer. If your individual protein is sensitive to pH, for example, with relation to protein activity or solubility, increase the pH to 7.5.

Buffer system: Use of phosphate buffers will result in reduced Factor Xa Protease activity compared to Tris·Cl or HEPES buffer systems.

Presence of detergents: Up to 1% Triton X-100, Nonidet P-40 and *n*-octylglucoside has no significant effect on Factor Xa Protease specificity or activity. The presence of SDS will significantly reduce enzyme activity.

Presence of denaturants: Significant loss of protease activity is observed at urea concentrations above 100 mM, and guanidine·HCl concentrations above 10 mM.

Presence of reductants: The presence of reductants in the reaction should be avoided, because the subunits of the heterodimeric protease enzyme are linked via a disulfide bridge. However, in some experiments up to 5 mM β-mercaptoethanol has been used with no significant influence on enzyme activity. The use of DTT is not recommended.

NaCl sensitivity: Factor Xa Protease activity decreases with increasing NaCl concentration. Significant inhibition is observed above 100 mM NaCl.

Imidazole sensitivity: Factor Xa Protease is significantly inhibited at or above 100 mM imidazole.

Anionic polymeric sugars: Some experiments have shown that addition of dextran sulfates (5000–10,000 Da) or heparin (5000 or 15,000 Da) to the reaction mixture results in increased Factor Xa Protease activity. 0.5–1 µg polymeric sugar can be added for cleavage of 10 µg recombinant protein in a 40 µl total reaction volume.

Amino acid sequence: Factor Xa Protease preferentially cleaves at the C-terminal side of the recognition sequence Ile-Glu-Gly-Arg↓X. It has been reported that X is in general unspecific, but Ile and Thr are preferred. Where X is a hydrophobic residue, cleavage efficiency is reduced (Owen et al., 1974, He et al., 1993).

Scaleup

Once the optimal cleavage conditions have been found, the reaction can be scaled up proportionally. Following the above protocol, 1 mg recombinant protein would be digested in a total volume of 4 ml. If there is a need to reduce the total reaction volume, perform small scale experiments in which the reaction volume is varied while the protease:recombinant protein ratio and incubation conditions are kept constant.

Protocol 20 b. Removal of Factor Xa Protease

After protease digestion, Factor Xa Protease can be removed by affinity chromatography using Xa Removal Resin. The Xa Removal Resin binds the protease in the reaction mixture while the cleaved recombinant protein remains in solution. After the resin is pelleted by centrifugation, the cleaved recombinant protein is recovered in the supernatant.

Materials

Xa Removal Resin

1x Reaction buffer (20 mM Tris·Cl, pH 6.5; 50 mM NaCl; 1 mM $CaCl_2$)

1. **Calculate the required amount of Xa Removal Resin necessary to capture the Factor Xa Protease present in the cleavage reaction.**

   50 µl bed volume (100 µl slurry) is sufficient to bind 4 Units Factor Xa Protease enzyme in 1x reaction buffer. Use of slurry volumes of less than 25 µl is not recommended due to associated handling problems. If your individual recombinant protein requires cleavage buffer other than the recommended 1x reaction buffer, bear in mind that the protease capture step may be sensitive to the use of other buffers. Binding of Factor Xa Protease to the Xa Removal Resin is unaffected by increasing the pH to 7.5, the presence of 20–100 mM Tris·HCl, and up to 1% Triton X-100 or Nonidet P-40. High salt concentrations will reduce binding capacity. For example, increasing NaCl concentration from 50 mM to 500 mM will result in a 20–40% reduction in binding. The recommended 1x reaction buffer (20 mM Tris·HCl, pH 6.5; 50 mM NaCl; 1 mM $CaCl_2$) supports high-efficiency cleavage and capture.

2. **Resuspend the Xa Removal Resin completely by gentle inversion and then immediately transfer the required amount of slurry into a centrifuge tube of appropriate size.**

   Note: The beads will quickly fall out of suspension. For transfer, use a wide-mouth pipette.

3. **Centrifuge the beads for 5 min at 1000 x *g* and discard the supernatant.**

4. **Resuspend the beads in ten bed-volumes of 1x reaction buffer by gently mixing, centrifuge for 5 min at 1000 x *g*, and discard the supernatant.**

   Equilibration of the beads with 1x reaction buffer is necessary for maximum capture efficiency and prevents contamination of the cleaved recombinant protein with resin storage buffer. Use Xa Removal Resin immediately after equilibration.

5. **Add the cleavage reaction to the equilibrated resin. Mix gently to resuspend the resin and incubate for 10 min at room temperature. Shake on an orbital shaker or place sealed tube on a roller-table to keep beads in suspension.**

   If the cleaved protein is temperature sensitive, binding can be performed at 4°C without any loss of binding efficiency.

6. **Centrifuge the reaction at 1000 x *g* for 5 min to pellet the resin. Collect the supernatant which contains the cleaved protein. Factor Xa Protease remains bound to the resin.**

Protocol 20 c. Removal of undigested 6xHis-tagged protein and cleaved 6xHis peptides after Factor Xa Protease cleavage

After digestion of 6xHis-tagged recombinant proteins with Factor Xa Protease, undigested proteins and released 6xHis-tag/Factor Xa Protease recognition site-peptides are present in the cleavage reaction mixture. Both these species can be removed by Ni-NTA affinity chromatography using a batch procedure, leaving pure, de-tagged recombinant protein in the supernatant. The protocol can be carried out either directly after Factor Xa Protease cleavage or subsequent to the removal of the protease with Xa Removal Resin. The protocol consists of two steps: adjusting the pH to 7.5, and binding of 6xHis-tagged contaminants to Ni-NTA resin.

Materials

1M Tris·Cl pH 8

Ni-NTA Agarose

1. **Adjust the pH of the cleavage reaction mixture to 7.5.**

   This can be accomplished by the addition of ~1/100 volume of 1M Tris·Cl, pH 8.0 when the recommended 1x reaction buffer (20 mM Tris·Cl, pH 6.5; 50 mM NaCl; 1 mM $CaCl_2$) was used for cleavage. It is not necessary to adjust the pH if the protease digestion was performed at pH 7.5. A pH of 7.5 is essential for efficient binding of 6xHis tags to Ni-NTA resin.

2. **Calculate the required amount of Ni-NTA Agarose needed to capture the 6xHis-tagged contaminants.**

   1 ml bed volume (2 ml slurry) is sufficient to bind 5–10 mg 6xHis-tagged protein. For optimal performance, the binding capacity of the Ni-NTA Agarose used for removal should match the total amount of 6xHis-tagged protein that was subjected to Factor Xa Protease cleavage.

3. **Resuspend the Ni-NTA Agarose completely by gently inverting the bottle 4–6 times and then immediately transfer the required amount of slurry into a centrifuge tube of appropriate size.**

4. **Centrifuge the resin for 1 min at 1000 x *g* and discard the supernatant.**

   Optional: To prevent contamination of the recombinant protein with Ni-NTA storage buffer, the pelleted beads can be washed with two bed volumes of 20 mM Tris·Cl, pH 7.5, 50 mM NaCl prior to incubation with the reaction mixture.

5. **Transfer the reaction mixture to the equilibrated resin. Mix gently to resuspend the resin and incubate for 10 min at room temperature. Shake on an orbital shaker or place sealed tube on a roller-table to keep beads in suspension.**

   If the recombinant protein is temperature sensitive, binding can be performed at 4°C without any loss of binding efficiency.

6. **Centrifuge the suspension at 1000 x *g* for 1 min to pellet the resin. Collect the supernatant that contains the pure recombinant protein. 6xHis-tagged contaminants remain bound to the resin.**

Troubleshooting: purification from *E. coli*

	Comments and suggestions
**Protein does not bind to the Ni-NTA resin**	
6xHis tag is not present.	Sequence ligation junctions to ensure that the reading frame is correct.
	Check for possible internal translation starts (N-terminal tag) or premature termination sites (C-terminal tag).
6xHis tag is inaccessible.	Purify protein under denaturing conditions.
	Move tag to the opposite end of the protein.
6xHis tag has been degraded.	Check that the 6xHis tag is not associated with a portion of the protein that is processed.
Binding conditions incorrect.	Check pH and composition of all buffers and solutions. Dissociation of urea often causes a shift in pH. The pH values should be checked immediately prior to use.
	Ensure that there are no chelating or reducing agents present, and that the concentration of imidazole is not too high.
**Protein elutes in the wash buffer**	
Wash stringency is too high.	Lower the concentration of imidazole or increase the pH slightly.
6xHis tag is partially hidden.	Reduce wash stringency. Purify under denaturing conditions.
Buffer conditions incorrect.	Check pH and composition of wash buffer.
	Ensure that there are no chelating or reducing agents present.
**Protein precipitates during purification**	
Temperature is too low.	Perform purification at room temperature.
Protein forms aggregates.	Try adding solubilization reagents such as 0.1% Triton X-100 or Tween-20, up to 20 mM β-ME, up to 2 M NaCl, or stabilizing cofactors such as Mg^{2+}. These may be necessary in all buffers to maintain protein solubility.

	Comments and suggestions
**Protein does not elute**	
Elution conditions are too mild (protein may be in an aggregate or multimer form).	Elute with a pH or imidazole step-gradient to determine the optimal elution conditions.
Protein has precipitated in the column.	Elute under denaturing conditions.
	Perform binding and elution in batch format to avoid high local protein concentrations.
**Protein elutes with contaminants**	
Binding and wash conditions not stringent enough.	Include 10–20 mM imidazole in the binding and wash buffers.
Column is too large.	Reduce the amount of Ni-NTA resin.
Contaminants are associated with tagged protein.	Add β-ME to a maximum of 20 mM to reduce disulfide bonds.
	Increase salt and/or detergent concentrations, or add ethanol/glycerol to wash buffer to disrupt nonspecific interactions.
Contaminants are truncated forms of the tagged protein.	Check for possible internal translation starts (C-terminal tag) or premature termination sites (N-terminal tag).
	Prevent protein degradation during purification by working at 4°C or by including protease inhibitors.
**Discoloration of resin**	
Nickel ions are removed or reduced.	Ensure that there are no chelating compounds (resin turns white in color) or reducing agents (resin turns brown in color) present in the buffers.

Troubleshooting: purification from mammalian cells

	Comments and suggestions
**No protein band in SDS-PAGE analysis of the eluate.**	
Expression is too low.	Check the expression level by western blotting using an Anti·His Antibody or a protein-specific antibody. Alternatively, perform an immunoassay with Ni-NTA Magnetic Agarose Beads (see the *Ni-NTA Magnetic Agarose Beads Handbook*) or ELISA using Ni-NTA HisSorb Strips (see the *QIA*express *Detection and Assay Handbook*). If only small amounts of 6xHis-tagged protein are present in the lysate, increase the amount of starting cell material and purify with an equal amount of magnetic beads. Do not exceed lysis volumes of 2 ml — this allows purification in a single 2 ml tube.
6xHis tagged protein has been degraded.	Check that the 6xHis tag is not removed from the protein during post-translational processing.<br>Work at 4°C and add protease inhibitors, such as PMSF.
6xHis-tagged protein partially elutes in the wash buffer or flow-through.	The binding capacity used is too low to bind all of the 6xHis-tagged protein. 10 µl magnetic-bead suspension has a binding capacity of 3 µg 6xHis-tagged DHFR (24 kDa). If significantly larger amounts of 6xHis-tagged protein are present in the lysate, increase the amount of beads accordingly.
**Binding of contaminants**	
Too much Ni-NTA matrix was used.	Match the total binding capacity of the beads to the amount of 6xHis-tagged protein to be purified by simply adjusting the amount of Ni-NTA Magnetic Agarose Beads suspension used.<br>Proteins that contain neighboring histidines are not common in bacteria, but do occur in eukaryotic cells. These proteins, as well as endogenous proteins with metal-binding sites, normally bind with lower affinity to the Ni-NTA matrix than do 6xHis-tagged proteins. If the binding capacity of the amount of beads used greatly exceeds the amount of 6xHis-tagged protein to be purified, these proteins will bind to the Ni-NTA matrix to a considerably higher extent, and will be subsequently recovered in the eluate.

	Comments and suggestions
Binding and wash conditions are not stringent enough.	Always include 10–20 mM imidazole in the binding buffer and 20 mM imidazole in the wash buffer.
Large amount of nontagged proteins in the lysate when purifying from cells with a very low expression rate.	Perform a second round of purification from the eluate after adjusting the imidazole concentration to 10–20 mM using binding buffer without imidazole. Significantly smaller amounts of background proteins in the binding step reduce the level of contaminants in the final preparation.

Troubleshooting: purification from insect cells

	Comments and suggestions
**No protein band in SDS-PAGE analysis of the fractions**	
No or low expression.	Check the expression level by western blotting using an Anti·His Antibody or a protein-specific antibody. Alternatively perform an ELISA using Ni-NTA HisSorb Strips (see *QIA*express *Detection and Assay Handbook*). If low amounts of 6xHis-tagged protein are present in the lysate, increase the amount of starting cell material and purify with an equal amount of Ni-NTA matrix.
6xHis-tagged protein has been degraded.	Check that the 6xHis tag is not removed from the protein during post-translational processing or by endogenous proteases during the purification procedure.
	Work at 4°C and add protease inhibitors, such as PMSF.
6xHis-tagged protein partially elutes in the wash buffer or flow-through.	The amount of matrix used is too low to bind all of the 6xHis-tagged protein. 100 µl Ni-NTA agarose has a binding capacity of 500–1000 µg 6xHis-tagged protein. Adjust the amount of matrix used for purification accordingly.
**Contaminants bind to resin**	
Too much Ni-NTA matrix was used.	Match the total binding capacity of the matrix used to the amount of 6xHis-tagged protein to be purified. Endogenous proteins with metal-binding sites normally bind with lower affinity to the Ni-NTA matrix than do 6xHis-tagged proteins. If the binding capacity of the amount of matrix used greatly exceeds the amount of 6xHis-tagged protein to be purified, these proteins will bind to the Ni-NTA matrix to a considerably higher extent, and subsequently will be recovered in the eluate.
Binding and wash conditions are not stringent enough.	Always include 10–20 mM imidazole in the binding buffer and 20 mM imidazole in the wash buffer.

Troubleshooting: Factor Xa Protease cleavage

	Comments and suggestions
**Factor Xa Protease cleavage**	
**No or incomplete cleavage of the recombinant protein**	
Insufficient Factor Xa Protease.	Increase the amount of Factor Xa Protease while keeping the recombinant protein concentration constant.
Recombinant protein concentration is too low.	Increase recombinant protein concentration. A minimum concentration of 0.25 µg/µl is recommended
Buffer components inhibit Factor Xa Protease.	Ensure that there are no buffer components that inhibit Factor Xa Protease activity (see important notes for optimization of cleavage, page 94). Dialyze the 6xHis-tagged protein sample against 20 mM Tris·Cl, pH 6.5; 50 mM NaCl; 1 mM $CaCl_2$ to achieve optimal cleavage conditions.
Incubation time and temperature are insufficient.	Increase incubation temperature up to 37°C and prolong incubation time up to 24 h.
**Multiple bands are observed on SDS-gel following cleavage with Factor Xa Protease**	
Bands derive from Factor Xa Protease.	If large amounts of Factor Xa Protease are used, two bands (approximately 17–20 kDa and 28–30 kDa) may appear on the gel under reducing conditions. Run Factor Xa Protease alone in an adjacent gel lane as a control.
Protease cleaves within the recombinant protein.	Check that no additional Factor Xa Protease recognition site is present in the recombinant protein. Reduce incubation temperature (RT or 4°C) to minimize exposure of secondary cleavage sites. Reduce amount of Factor Xa Protease used for cleavage.
	Adjust reaction conditions to obtain partial digestion which may result in selective scission at the desired Factor Xa Protease recognition site.

Factor Xa Removal

Factor Xa Protease not efficiently removed

Amount of Xa Removal Resin used is too low.	Check that you have correctly calculated the amount of resin necessary (see Protocol).<br>Perform a second capture reaction.
Buffer contains components which affect the Factor Xa Protease binding reaction (see protocol).	Dialyze the cleavage reaction mixture against 1x Reaction Buffer and repeat the binding protocol.<br>Try to eliminate incompatible buffer components during the Factor Xa Protease cleavage reaction and repeat the cleavage and capture steps.

Removal of 6xHis-tagged polypeptides

6xHis-tagged peptides bind incompletely or not at all to the Ni-NTA resin.

Binding conditions are incorrect.	Check the pH and composition of reaction mixture. Ensure that there are no chelating or reducing agents present.
Binding capacity of Ni-NTA resin does not match total amount of 6xHis-tagged polypeptides.	Perform a second capture reaction.

Cleaved recombinant protein band intensity is reduced in SDS-PAGE analysis

Cleaved recombinant protein binds to Ni-NTA resin.	Add 20 mM imidazole to the reaction mixture to prevent non-specific binding of the cleaved recombinant protein.

Protein Refolding Recommendations

It is generally preferable to be able to purify recombinant proteins under native conditions, but for many proteins, particularly when large yields are required, this may not always be possible. If proteins are purified under denaturing conditions for use in antibody induction, there is usually no need to renature before injection into the animal. If renaturation is necessary, refolding conditions must be determined empirically for each individual protein. Detailed examples of refolding procedures have been described (Wingfield et al. 1995a). The following are a few recommendations that may be helpful in designing refolding experiments.

- Maintain low protein concentration (10–50 µg/ml).
- Include thiol reagents.

  Disulfide bonds contribute to the stability of the native conformation of many proteins. Secondary structure formation may be so favorable that the correct cysteine residues spontaneously bind, i.e., weakly oxidizing conditions are required. If, however, this is not the case, stronger oxidizing conditions may be required. A redox pair of reduced glutathione (GSH) and oxidized glutathione (GSSG) creates the necessary oxidizing potential to make and break disulfide bonds in folding intermediates, thereby allowing the optimal, native conformation to be reached. A GSH:GSSG ratio of 10:1 (at a concentration of 2–5 mM GSH) has been shown to be effective for a number of proteins.
- Remove denaturants slowly by dilution or dialysis.

  Glycine often has remarkable solubilization properties (50 mM, pH 9.0; 5 mM EDTA). If GuHCl must be used as a denaturant, urea included in the renaturation buffer (2 M) may stabilize the protein upon refolding. Very low concentrations of detergents may also be included (0.1–0.5% NP-40 or 0.005% (v/v) Tween 20).
- Include cosolvents.

  Many cosolvents such as glycerol (5–20%) and ethylene glycol, as well as glucose and sucrose (10%) can stabilize proteins. Certain anions (e.g., phosphate and sulfate) and cations (e.g., MES, HEPES) also have positive effects. They exhibit little or no effect on the folding rate constant, but decrease the unfolding rate constant. They act by stabilizing hydrophobic interactions, but care should be taken because they can also stabilize aggregate formation.
- Include salt and maintain neutral pH.

  100 mM KCl, or 150–500 mM NaCl, 2 mM $MgCl_2$

  10–50 mM HEPES-KOH (pH 7.5–7.9)
- Suppress proteolytic degradation with protease inhibitors.

  0.5 mM PMSF, 0.05–2 µg/ml aprotinin, 2 µg/ml pepstatin, or 2–5 µg/ml leupeptin

Many proteins that are insoluble when refolded in solution can be successfully refolded while immobilized on the Ni-NTA matrix. Immobilizing one end of the protein during renaturation appears to prevent intermolecular interactions which lead to aggregate formation. Renature using a linear 6 M – 1 M urea gradient in 500 mM NaCl; 20% glycerol; 20 mM Tris·Cl, pH 7.4, containing protease inhibitors. The gradient should be performed using FPLC equipment to ensure linearity (conventional gradient makers often produce nonlinear gradients), and the renaturation should be performed over a period of 1.5 h. After renaturation, proteins can be eluted by the addition of 250 mM imidazole. See also Holzinger et al. (1996).

Appendix

Ni-NTA Matrices

Specifications

	**Ni-NTA Agarose**	**Ni-NTA Superflow**
Application:	Gravity-flow column or batch purification; small to medium scale	FPLC, gravity-flow column, or batch purification; small to production scale
Binding capacity:	5–10 mg/ml (300–500 nmol @ ~20 kDa)	5–10 mg/ml (300–500 nmol @ ~20 kDa)
Support:	Sepharose CL-6B	Superflow
Bead structure:	Cross-linked 6% agarose	Highly cross-linked 6% agarose
Bead size:	45–165 µm	60–160 µm
Exclusion limit (MW):	>>4 x 10^7	4 x 10^6
Max. linear flow rate:	75–150 cm/h*	3000 cm/h*
Max. volumetric flow rate:	0.5–1.0 ml/min*	20 ml/min*
Recommended flow rate†:	0.5 ml/min	1–3 ml/min
Max. pressure:	2.8 psi (0.2 bar)	140 psi (10 bar)
pH stability (≤2 h):	2–14	2–14
pH stability (>2 h):	3–12	3–12
Form:	50% resin suspension in 30% ethanol, precharged with Ni^{2+}	50% resin suspension in 30% ethanol, precharged with Ni^{2+}
Antimicrobial agent:	30% ethanol	30% ethanol
Storage:	RT or 4°C‡ (do not freeze)	RT or 4°C‡ (do not freeze)
Availability:	Separately or in QIA*express* Kits	Bulk resin

** Determined in a column of 1 cm diameter, 8 ml bed volume*
† For binding step
‡ 4°C is recommended for long-term storage

Handling

Ni-NTA matrices are stable under a wide variety of conditions and need not be refrigerated, except to inhibit growth of microorganisms for long-term storage. After use they should be washed for 30 minutes with 0.5M NaOH. Ni-NTA matrices should be stored in 30% ethanol to inhibit microbial growth. The matrix can be stored for up to one week in any of the denaturing buffers.

Reuse of Ni-NTA Resin

The reuse of Ni-NTA resin depends on the nature of the sample and should only be performed with identical recombinant proteins. Based on the experience of Hoffmann-La Roche Ltd. (Basel, Switzerland), who have purified more than 100 different proteins on Ni-NTA resin, we recommend a maximum of 5 runs per column.

If the Ni-NTA Agarose changes from light blue to brownish-gray, the following regeneration procedure is recommended.

Procedure:

1. Wash the column with 2 volumes of Regeneration Buffer (6 M GuHCl, 0.2 M acetic acid).
2. Wash the column with 5 volumes of H_2O.
3. Wash the column with 3 volumes of 2% SDS.
4. Wash the column with 1 volume of 25% EtOH.
5. Wash the column with 1 volume of 50% EtOH.
6. Wash the column with 1 volume of 75% EtOH.
7. Wash the column with 5 volumes of 100% EtOH.
8. Wash the column with 1 volume of 75% EtOH.
9. Wash the column with 1 volume of 50% EtOH.
10. Wash the column with 1 volume of 25% EtOH.
11. Wash the column with 1 volume of H_2O.
12. Wash the column with 5 volumes of 100 mM EDTA, pH 8.0.
13. Wash the column with H_2O.
14. Recharge the column with 2 volumes of 100 mM $NiSO_4$.
15. Wash the column with 2 volumes of H_2O.
16. Wash the column with 2 volumes of Regeneration Buffer.
17. Equilibrate with 2 volumes of a suitable buffer (e.g., Buffer A or B).

Preparation of guanidine-containing samples for SDS-PAGE

Since fractions that contain GuHCl will form a precipitate when treated with SDS, they must either be diluted with H_2O (1:6), dialyzed before analysis, or separated from the GuHCl by trichloroacetic acid (TCA) precipitation.

TCA precipitation

1. Dilute samples to 100 µl; add equal volumes of 10% TCA.
2. Leave on ice for 20 min; centrifuge for 15 min in a microcentrifuge.
3. Wash pellet with 100 µl of ice-cold ethanol, dry, and resuspend in sample buffer.

   In case there are any traces of GuHCl present, samples should be loaded immediately after boiling for 7 min at 95°C.

Media, solutions, and reagents

Bacterial media and solutions

LB medium:	10 g/liter tryptone, 5 g/liter yeast extract, 10 g/liter NaCl
LB agar:	LB medium containing 15 g/liter agar
Psi broth:	LB medium, 4 mM $MgSO_4$, 10 mM KCl
Kanamycin stock solution:	25 mg/ml in H_2O, sterile filter, store in aliquots at –20°C
Ampicillin stock solution:	100 mg/ml in H_2O, sterile filter, store in aliquots at –20°C
IPTG (1 M):	238 mg/ml in H_2O, sterile filter, store in aliquots at –20°C

Buffers for preparing competent *E. coli*

TFB1:	100 mM RbCl, 50 mM $MnCl_2$, 30 mM potassium acetate, 10 mM $CaCl_2$, 15% glycerol, pH 5.8;* sterile-filter
TFB2:	10 mM MOPS, 10 mM RbCl, 75 mM $CaCl_2$, 15% glycerol, adjust to pH 6.8 with KOH, sterile filter

SDS-PAGE sample buffers

2x SDS-PAGE sample buffer:	0.09 M Tris·Cl, pH 6.8; 20% glycerol; 2% SDS; 0.02% bromophenol blue; 0.1 M DTT
5x SDS-PAGE sample buffer:	0.225 M Tris·Cl, pH 6.8; 50% glycerol; 5% SDS; 0.05% bromophenol blue; 0.25 M DTT

Solutions for colony-blot procedure

SDS solution:	10% (w/v) sodium dodecyl sulfate
Denaturing solution:	0.5 M NaOH, 1.5 M NaCl
Neutralization solution:	1.5 M NaCl; 0.5 M Tris·Cl, pH 7.4 (25°C)
20x SSC:	500 ml: 87.65 g NaCl, 50.25 g trisodium citrate·$2H_2O$
TBS buffer:	10 mM Tris·Cl, pH 7.5; 150 mM NaCl
TBS-Tween buffer:	20 mM Tris·Cl, pH 7.5; 500 mM NaCl; 0.05% Tween 20 (Sigma, P1379)
TBS-Tween/Triton buffer:	20 mM Tris·Cl, pH 7.5; 500 mM NaCl; 0.05% Tween 20 (Sigma, P1379); 0.2% Triton X-100 (Sigma, X-100)

** Adjust pH carefully as insoluble Mn precipitates can form.*

Blocking buffer:	3% BSA (Sigma, A7906) in TBS buffer
Antibody stock solution:	Dissolve the lyophilized RGS·His Antibody in 500 µl H_2O per vial (final concentration, 0.2 mg/ml). Can be stored at 4°C for up to 6 months or in aliquots at –20°C for up to one year.

Alkaline phosphatase (AP) staining solutions

Development buffer:	100 mM Tris·Cl, pH 9.5; 100 mM NaCl; 5 mM $MgCl_2$
NBT stock solution:	5% NBT (nitro blue tetrazolium chloride, Sigma N5514) in 70% dimethylformamide (store in aliquots at –20°C)
BCIP stock solution:	5% BCIP (5-bromo-4 chloro-3-indolyl phosphate, Sigma B0274) in 100% dimethylformamide (store in aliquots at –20°C)
Staining solution:	**Note**: prepare immediately before staining. 66 µl NBT and 33 µl BCIP stock solutions in 10 ml development buffer (final concentration: 0.33 mg/ml NBT; 0.165 mg/ml BCIP). As an alternative, BCIP/NBT tablets can be obtained from Sigma (B5655).

Horseradish peroxidase staining solutions

Tris-saline:	0.9% NaCl in 0.1 M Tris·Cl, pH 8.0
Staining solution:	**Note**: Prepare immediately before use. Dissolve 18 mg 4-chloro-1-naphthol (Sigma, C8890) in 6 ml methanol, add 24 ml Tris-saline followed by 60 µl 30% hydrogen peroxide (H_2O_2).

Buffers for purification under denaturing conditions (protocols 5, 10, 17, 18, and 19)

Lysis buffers

Buffer A (1 liter):

100 mM NaH_2PO_4	13.8 g $NaH_2PO_4 \cdot H_2O$ (MW 137.99 g/mol)
10 mM Tris·Cl	1.2 g Tris base (MW 121.1 g/mol)
6 M GuHCl	573 g guanidine hydrochloride

Adjust pH to 8.0 using NaOH.

Buffer B (1 liter):

100 mM NaH_2PO_4	13.8 g $NaH_2PO_4 \cdot H_2O$ (MW 137.99 g/mol)
10 mM Tris·Cl	1.2 g Tris base (MW 121.1 g/mol)
8 M urea	480.5 g (MW 60.06 g/mol)

Adjust pH to 8.0 using NaOH.

Wash buffer

Buffer C (1 liter):

100 mM NaH_2PO_4	13.8 g $NaH_2PO_4 \cdot H_2O$ (MW 137.99 g/mol)
10 mM Tris·Cl	1.2 g Tris base (MW 121.1 g/mol)
8 M urea	480.5 g (MW 60.06 g/mol)

Adjust pH to 6.3 using HCl.

Elution buffers

Buffer D (1 liter):

100 mM NaH_2PO_4	13.8 g $NaH_2PO_4 \cdot H_2O$ (MW 137.99 g/mol)
10 mM Tris·Cl	1.2 g Tris base (MW 121.1 g/mol)
8 M urea	480.5 g (MW 60.06 g/mol)

Adjust pH to 5.9 using HCl.

Buffer E (1 liter):

100 mM NaH_2PO_4	13.8 g $NaH_2PO_4 \cdot H_2O$ (MW 137.99 g/mol)
10 mM Tris·Cl	1.2 g Tris base (MW 121.1 g/mol)
8 M urea	480.5 g (MW 60.06 g/mol)

Adjust pH to 4.5 using HCl.

Note: Due to the dissociation of urea, the pH of Buffers B, C, D, and E should be adjusted immediately prior to use. Do not autoclave.

Buffers for purification under native conditions (from *E. coli* and insect cells; protocols 6, 9, 11, 12, 13, 14, and 16)

Lysis buffer (1 liter):

50 mM NaH_2PO_4	6.90 g $NaH_2PO_4 \cdot H_2O$ (MW 137.99 g/mol)
300 mM NaCl	17.54 g NaCl (MW 58.44 g/mol)
10 mM imidazole	0.68 g imidazole (MW 68.08 g/mol)

Adjust pH to 8.0 using NaOH.

Wash buffer (1 liter):

50 mM NaH_2PO_4	6.90 g $NaH_2PO_4 \cdot H_2O$ (MW 137.99 g/mol)
300 mM NaCl	17.54 g NaCl (MW 58.44 g/mol)
20 mM imidazole	1.36 g imidazole (MW 68.08 g/mol)

Adjust pH to 8.0 using NaOH.

Elution buffer (1 liter):

50 mM NaH_2PO_4	6.90 g $NaH_2PO_4 \cdot H_2O$ (MW 137.99 g/mol)
300 mM NaCl	17.54 g NaCl (MW 58.44 g/mol)
250 mM imidazole	17.00 g imidazole (MW 68.08 g/mol)

Adjust pH to 8.0 using NaOH.

Buffers for purification from mammalian cells using Ni-NTA Magnetic Agarose Beads under native conditions (Protocol 15)

PBS 50 mM potassium phosphate, pH 7.2; 150 mM NaCl

Lysis buffer (1 liter):

50 mM NaH_2PO_4	6.90 g $NaH_2PO_4 \cdot H_2O$ (MW 137.99 g/mol)
300 mM NaCl	17.54 g NaCl (MW 58.44 g/mol)
10 mM imidazole	0.68 g imidazole (MW 68.08 g/mol)
0.05% Tween 20	5 ml of a 10% Tween 20 stock solution

Adjust pH to 8.0 using NaOH.

Wash buffer (1 liter):

50 mM NaH_2PO_4	6.90 g $NaH_2PO_4 \cdot H_2O$ (MW 137.99 g/mol)
300 mM NaCl	17.54 g NaCl (MW 58.44 g/mol)
20 mM imidazole	1.36 g imidazole (MW 68.08 g/mol)
0.05% Tween 20	5 ml of a 10% Tween 20 stock solution

Adjust pH to 8.0 using NaOH.

Elution buffer (1 liter):

50 mM NaH_2PO_4	6.90 g $NaH_2PO_4 \cdot H_2O$ (MW 137.99 g/mol)
300 mM NaCl	17.54 g NaCl (MW 58.44 g/mol)
250 mM imidazole	17.00 g imidazole (MW 68.08 g/mol)
0.05% Tween 20	5 ml of a 10% Tween 20 stock solution

Adjust pH to 8.0 using NaOH.

Buffer for Factor Xa Protease digestion and removal of Factor Xa Protease with Xa Removal Resin (Protocols 20 a and 20 b)

1x reaction buffer (1 liter):

20 mM Tris·Cl	2.42 g Tris base (MW 121.1 g/mol)
50 mM NaCl	2.92 NaCl (MW 58.44 g/mol)
1 mM $CaCl_2$	0.147 g $CaCl_2 \cdot 2H_2O$ (MW 147.02 g/mol)

Adjust to pH 6.5 using HCl.

QIA*express* pQE vectors: multiple cloning sites

pQE vectors are designed for cloning and expression of 6xHis-tagged proteins. Complete sequences of pQE vectors can be found at **www.qiagen.com**.

pQE-9

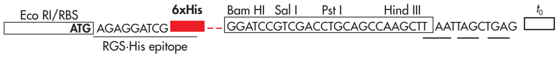

pQE-16

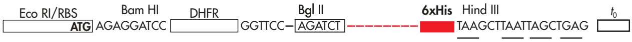

pQE-30/pQE-80 L

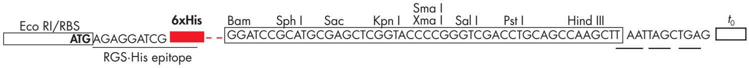

pQE-31/pQE-81 L

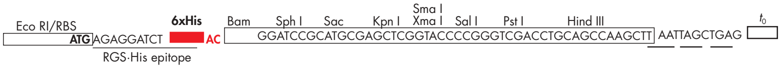

pQE-32/pQE-82 L

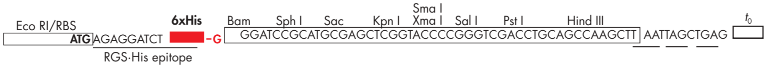

pQE-40

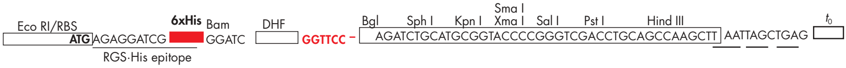

pQE-60

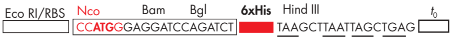

pQE-70

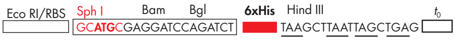

pQE-100 DoubleTag

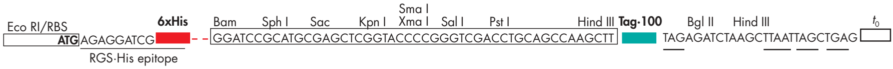

pQE-30 UA

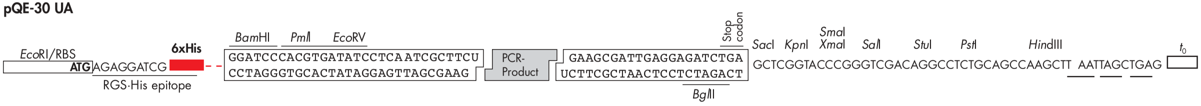

pQE-30 Xa

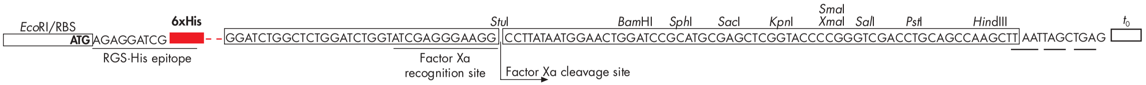

pQE-TriSystem*

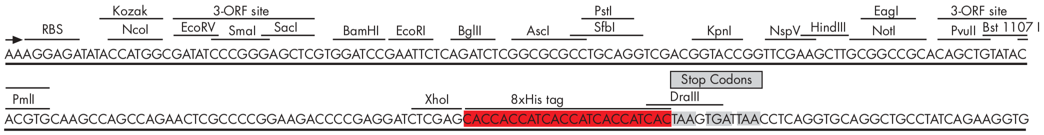

*The GC-rich region upstream of the intron may cause problems in sequencing. We recommend linearization of the vector using a restriction site in the intron, eg., XbaI.

Restriction map of pREP4

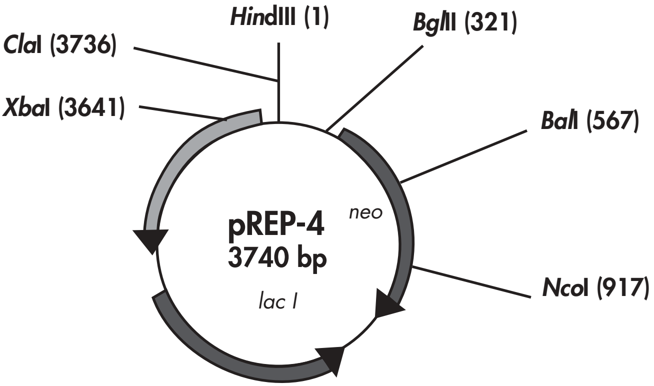

Restriction map of pREP4

Sequencing primers for pQE vectors*

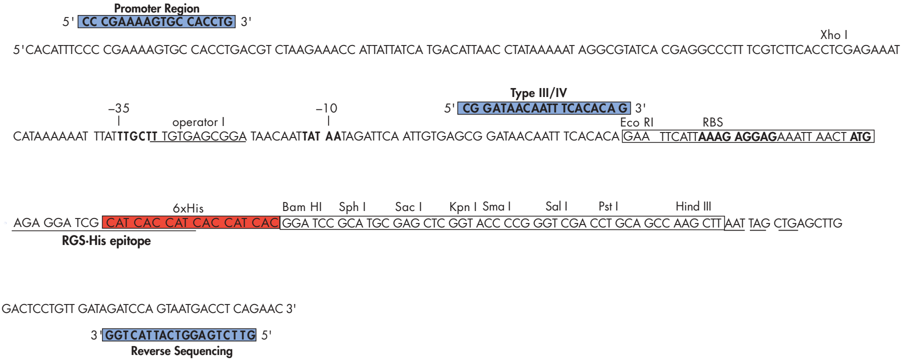

* *Sequencing primers can be used for all pQE vectors except pQE-TriSystem.*

Appendix

References

Abate, C., Luk, D., Gentz, R., Rauscher III, F. J., and Curran, T. (1990) Expression and purification of the leucine zipper and DNA-binding domains of Fos and Jun: both Fos and Jun contact DNA directly. Proc. Natl. Acad. Sci. USA ***87****, 1032–1036.*

Ausubel, F.M., Brent, R., Kingston, R.E., Moore, D.D., Sedman, J.G., Smith, J.A., and Struhl, K. eds. (1995) Current Protocols in Molecular Biology. New York: John Wiley and Sons.

Baldarini, C. and Cesareni, G. (1985) Plasmid pEMBLy: new single-stranded shuttle vectors for the recovery and analysis of yeast DNA sequences. Gene ***35****, 27–32.*

Bowden, G.A. and Georgiou, G. (1990) Folding and aggregation of ß-lactamase in the periplasmic space of Escherichia coli. *J. Biol. Chem.* ***265****, 16760–16766.*

Bradley, M.K. (1990) Overexpression of proteins in eukaryotes. Methods Enzymol. ***182****, 112–143.*

Bujard, H., Gentz, R., Lanzer, M., Stüber, D. Müller, M., Ibrahimi, I. Häuptle, M.T., and Dobberstein, B. (1987) A T5 promotor based transcription-translation system for the analysis of proteins in vivo and in vitro. Methods Enzymol. ***155****, 416–433.*

Bush, G.L., Tassin, A., Friden, H., and Meyer, D.I. (1991) Secretion in yeast: purification and in vitro translocation of chemical amounts of prepro-α-factor. J. Biol. Chem. ***266****, 13,811–13,814.*

Chang, A.C.Y. and Cohen, S.N. (1978). Construction and characterization of amplifiable multicopy DNA cloning vehicles derived from the PI5A cryptic miniplasmid. J. Bacteriol. ***134****, 1141–1156.*

Clare, J. J., Romanos, M. A., Rayment, F. B., Rowedder, J. E., Smith, M. A., Payne, M. M., Sreekrishna, K., Henwood, C. A., (1991) Production of mouse epidermal growth factor in yeast: high-level secretion using Pichia pastoris *strains containing multiple copies. Gene* ***105****, 205–212.*

Coligan, J. E., Dunn, B. M., Ploegh, H. L., Speicher, D. W., and Wingfield, P.T. eds. (1995) Current protocols in protein science, vol. 1, John Wiley and Sons, New York.

Deutscher, M.P. ed. (1990). Methods in Enzymology. Volume 185. Guide to Protein Purification. Academic Press, San Diego, CA, USA.

Dobeli, H., Trecziak, A., Gillessen, D., Matile, H., Srivastava, I. K., Perrin, L. H., Jakob, P. E., and Certa, U. (1990) Expression, purification, biochemical characterization and inhibition of recombinant Plasmodium falciparum *aldolase. Molec. Biochem. Parasitol.* ***41****, 259-268.*

Farabaugh, P.J. (1978) Sequence of the lac I gene. Nature 274, 765.

Flachmann, R. and Kühlbrandt, W. (1996) Crystallization and assembly defect of recombinant antenna complexes produced in transgenic tobacco plants. Proc. Nat. Acad. Sci. USA ***93****, 14966–14971.*

Gentz, R., Certa, U., Takacs, B. J., Matile, H., Dobeli, H., Pink, R., Mackay, M., Bone, N., and Scaife, J. G. (1988) Major surface antigen pl90 of Plasmodium falciparum*: detection of common epitopes present in a variety of plasmodia isolates. EMBO J.* ***7****, 225–230.*

Gentz, R., Chen, C., and Rosen, C. A. (1989) Bioassay for trans-activation using purified immunodeficiency virus tat-encoded protein: trans-activation requires mRNA synthesis. Proc. Natl. Acad. Sci. USA ***86****, 821–824.*

Gietz, D., Jean, A. S., Woods, R. A., and Schiestl, R. H. (1992) Improved method for high efficiency transformation of intact yeast cells. Nucl. Acids Res. ***20****, 1425.*

Gottesman, S., Halpern, E., and Trisler, P. (1981) Role of sulA and sulB in filamentation by lon mutants of Escherichia coli K-12. J. Bacteriol. ***148****, 265–273.*

Grosjean, H. and Fiers, W. (1982) Preferential codon usage in prokaryotic genes: the optimal codon-anticodon interaction energy and the selective codon usage in efficiently expressed genes. Gene ***18****, 199–209.*

Gu, J., Stephenson, C.G., and Iadarola M.J. (1994) Recombinant proteins attached to a Ni-NTA column: Use in affinity purification of antibodies. BioTechniques ***17****, 257–262.*

Guan, C., Li, P., Riggs,. P.D., and Inouye, H. (1988) Vectors that facilitate the expression and purification of foreign peptides in Escherichia coli *by fusion to maltose binding protein. Gene* ***67****, 21–30.*

Guthrie, C. and Fink, G. R. eds. (1992) Methods in Enzymology. Volume 194. Guide to yeast genetics and molecular biology. Academic Press, San Diego, CA, USA.

He, M., Jin, L., and Austen, B. (1993) J. Protein Chem. 12(1), 1-5.

Hochuli, E. (1989) Genetically designed affinity chromatography using a novel metal chelate absorbent, Biologically Active Molecules 217–239.

Hochuli, E. (1990) Purification of recombinant proteins with metal chelate adsorbant. In: Genetic engineering, principle and method. Setlow, J.K., ed. ***12****, 87–98.*

Hochuli, E., Bannwarth, W., Dobeli, H., Gentz, R., and Stüber, D. (1988) Genetic approach to facilitate purification of recombinant proteins with a novel metal chelate adsorbent. Bio/Technology ***6****, 1321–1325.*

Hochuli, E., Dobeli, H., and Schacher, A. (1987) New metal chelate adsorbent selective for proteins and peptides containing neighboring histidine residues. J. Chromatog. ***411****, 177–184.*

Holzinger, A., Phillips, K.S. and Weaver, T.E. (1996) Single-step purification/solubilization of recombinant proteins: application to surfactant protein B. BioTechniques ***20****, 804–808.*

Jaenicke, R. and Rudolph, R. (1990). Folding proteins. In: Protein structure – a practical approach. Creighton, T.E. ed. IRL Press, New York.

Janknecht, R., de Martynoff, G., Lou, J., Hipskind, R.A., Nordheim, A., and Stunnenberg, H. G. (1991) Rapid and efficient purification of native histidine-tagged protein expressed by recombinant vaccinia virus. Proc. Natl. Acad. Sci. USA ***88****, 8972-8976.*

Kaslow, D. C. and Shiloach, J. (1994) Production, purification, and immunogenicity of a malaria transmission-blocking vaccine candidate: TBV25H expressed in yeast and purified using Ni-NTA-agarose. BioTechnology ***12****, 494–499.*

LaVallie, E.R., DiBlasio, E.A., Kovacic, S., Grant, K.L., Schendel, P.F., and McCoy, J.M. (1993) A thioredoxin gene fusion system that circumvents inclusion body formation in the E. coli *cytoplasm. Bio/Technology* ***11****, 187–193.*

Le Grice, S.F.J. and Grueninger-Leitch, F. (1990) Rapid purification of homodimer HIV-I reverse transcriptase by metal chelate affinity chromatography. Eur. J. Biochem. ***187****, 307–314.*

Lindner, P., Guth, B., Wülfing, C., Krebber, C., Steipe, B., Müller, F., and Plückthun, A. (1992) Purification of native proteins form the cytoplasm and periplasm of Escherichia coli *using IMAC and histidine tails: A comparison of proteins and protocols. Methods: A Companion to Methods in Enzymology* ***4****, 41–55.*

Luckow, V. A. (1991) Cloning and expression of heterologous genes in insect cells with baculovirus vectors. In: Recombinant DNA technology and applications, Prokop, A., Bajpai, R.K., and Ho, C., eds. McGraw-Hill, New York. pp. 97–152.

Maina, C.V., Riggs, P.D., Grandia, A.G., Slatko, B.E., Moran, L.S., Tagliamonte, J.A., McReynolds, L.A., and Guan, C. (1988) An Escherichia coli *vector to express and purify foreign proteins by fusion to and separation from maltose binding protein. Gene* ***74****, 365–373.*

Masters, J.N., and Attardi, G. (1983). The nucleotide sequence of the cDNA coding for the human dihydrofolic acid reductase. Gene ***21****, 59–63.*

Miller, L.K. (1988) Baculoviruses as gene expression vectors. Annu. Rev. Microbiol. ***42****, 177–199.*

Miyazaki, J., Takaki, S.; Tashiro, F., Tominaga A., Takatsu, K., Yamamura K., (1989), Gene ***79****, 269-277*

O'Reilly, D.R., Miller, L.K., and Luckow, V.A. (1992) Baculovirus Expression Vectors. W.H. Freeman and Company, New York.

Owen, W.G., Esmon, C.T., and Jackson, C.M., (1974) J. Biol. Chem. ***249*** *, 594-605*

Palmer, I. and Wingfield, P.T. (1995) Preparation and extraction of insoluble (inclusion-body) proteins from Escherichia coli. *In: Current protocols in protein science, Vol. 1, Coligan, J. E., Dunn, B. M., Ploegh, H. L., Speicher, D. W., and Wingfield, P.T., eds. John Wiley and Sons, New York, pp. 6.3.1–6.3.15.*

Porath, J., Carlsson, J., Olsson, I., and Belfrage, G. (1975) Metal chelate affinity chromatography, a new approach to protein fractionation. Nature ***258,*** *598–599.*

Romanos, M. A., Scorer, C. A., and Clare, J. J. (1992). Foreign gene expression in yeast: a review. Yeast ***8****, 423–488.*

Rosenberg, A.H., Goldman, E., Dunn, J.J., Studier, F.W., and Zubay, G. (1993) Effects of consecutive AGG codons on translation in Escherichia coli *demonstrated with a versatile codon test system. J. Bacteriol* ***175****, 716–722.*

Sambrook, J., Fritsch, E.F., and Maniatis, T. (1989). Molecular Cloning: A Laboratory Manual, 2nd Edition, New York: Cold Spring Harbor Laboratory Press, Cold Spring Harbor.

Schäfer, F., Römer, U., Emmerlich, M., Blümer, J., Lubenow, H., and Steinert, K. (2002a). Automated High-Throughput Purification of 6xHis-Tagged Proteins. J. Biomol. Tech. ***13****, 131.*

Schäfer, F., Schäfer, A., and Steinert, K. (2002b). A Highly Specific System for Efficient Enzymatic Removal of Tags from Recombinant Proteins. J. Biomol. Tech. ***13****, 158.*

Schein, C.H. (1989) Production of soluble recombinant proteins in bacteria. Bio/Technology ***7****, 1141–1149.*

Schein, C.H. and Noteborn, M.H.M. (1988) Formation of soluble recombinant proteins in Escherichia coli *is favored by low growth temperature. BioTechnology* ***6****, 291–294.*

Schmitt, J., Hess, H., and Stunneberg, H.G. (1993a) Affinity purification of histidine-tagged proteins. Molecular Biology Reports ***18****, 223–230.*

Schwarz, E., Scherrer, G., Hobom, G., and Kössel, H. (1978). Nucleotide sequence of cro, cII *and part of the* 0 *gene in phage lambda DNA. Nature* ***272****, 410–414.*

Shine, J. and Dalgarno, L. (1974) The 3′-terminal sequence of Escherichia coli *16S ribosomal RNA: Complementary to nonsense triplets and ribosome binding sites. Proc. Nat. Acad. Sci. U.S.A.* ***71****, 1342–1346.*

Smith, D.B. and Johnson, K.S. (1988) Single-step purification of polypeptides expressed in Escherichia coli *as fusions with glutathione S-transferase. Gene* ***67****, 31–40.*

Stüber, D., Bannwarth, W., Pink, J. R. L., Meloen, R. H., and Matile, H. (1990) New B-cell epitopes in the Plasmodium falciparum *malaria circumsporozoite protein. Eur. J. Immunol.* ***20****, 819–824.*

Stüber, D., Matile, H., and Garotta, G. (1990) System for high-level production in Escherichia coli *and rapid purification of recombinant proteins: application to epitope mapping, preparation of antibodies, and structure-function analysis. In: Immunological Methods, Lefkovits, I. and Pernis, B., eds., vol. IV, Academic Press, New York, pp. 121–152.*

Studier, F.W., Rosenberg, A.H., Dunn, J.J., and Dubendorff, J.W. (1990) Use of T7 RNA polymerase to direct expression of cloned genes. Methods Enzymol. ***185****, 60–89.*

Sulkowski, E. (1985) Purification of proteins by IMAC. Trends Biotechnol. ***3****, 1–7.*

Sutcliffe, J.G. (1979). Complete nucleotide sequence of the E. coli *plasmid pBR322. Cold Spring Harbor Symp. Quant. Biol.* ***43****, 77–90.*

Takacs, B. J. (1979) Immunological methods, *Lefkovits, I. and Pernis, B., eds., vol. 1, Academic Press, New York, p. 81.*

Takacs, B. J. and Girard, M.-F. (1991) Preparation of clinical grade proteins produced by recombinant DNA technologies. J. Immunol. Methods ***143****, 231–240.*

Waeber, U., Buhr, A., Schunk, T., and Erni, B. (1993) The glucose transporter of Escherichia coli*: purification and characterization by Ni^{+2} chelate affinity chromatography of the $IIBC^{Glc}$ subunit. FEBS Letters* ***324****, 109–112.*

Wahle, S., Rohweder, H., Ribbe, J., and Steinert, K. (1999) Purification of 6xHis-tagged proteins from mammalian expression systems using Ni-NTA Magnetic Agarose Beads. QIAGEN News 1999 No. 4, 3.

Wingfield, P. T. (1995a) Overview of the purification of recombinant proteins produced in Escherichia coli. *In: Current protocols in protein science, vol. 1, Coligan, J. E., Dunn, B. M., Ploegh, H. L., Speicher, D. W., and Wingfield, P.T. eds. John Wiley and Sons, New York, pp. 6.1.1–6.1.22.*

Wingfield, P. T. (1995b) Preparation of soluble proteins from Escherichia coli. *In:* Current protocols in protein science, vol. 1, *Coligan, J. E., Dunn, B. M., Ploegh, H. L., Speicher, D. W., and Wingfield, P.T., eds. Wiley and Sons, New York, pp. 6.2.1–6.2.15.*

Wingfield, P. T., Palmer, I., and Liang, S.-M. (1995) Folding and purification of insoluble (inclusion-body) proteins from Escherichia coli. *In: Current protocols in protein science, vol. 1, Coligan, J. E., Dunn, B. M., Ploegh, H. L., Speicher, D. W., and Wingfield, P.T. eds. Wiley and Sons, Inc. New York, pp. 6.5.1–6.5.27.*

Ordering Information

Product	Contents	Cat. No.
**QIA*express* Kits**		
QIA*express* Type IV Kit	pQE-30, pQE-31, pQE-32 (N-terminal 6xHis)	32149
QIA*express* Type ATG Kit	pQE-60, pQE-70 (C-terminal 6xHis)	32169
**pQE expression vectors**		
C-Terminus pQE Vector Set	25 µg each: pQE-16, pQE-60, pQE-70	32903
N-Terminus pQE Vector Set	25 µg each: pQE-9, pQE-30, pQE-31, pQE-32, pQE-40	32915
cis-Repressed pQE Vector Set	25 µg each: pQE-80L, pQE-81L, pQE-82L	32923
pQE-100 DoubleTag Vector DNA	25 µg pQE-100 (lyophilized)	33003
pQE Sequencing-Primer Set	0.1 A_{260} unit each: Primer - Promoter Region, Primer - Type III/IV, Primer - Reverse Sequencing (3.0, 2.8, 3.1 µg, respectively; lyophilized)	34051
E. coli Host Strains	One stab culture each: *E. coli* M15[pREP4], SG13009[pREP4]	34210
pQE-30 Xa Vector	25 µg pQE-30 Xa Vector DNA	33203
pQE-TriSystem Vector	25 µg pQE-TriSystem Vector DNA	33903
QIA*express* UA Cloning Kit	100 µl 2x Ligation Master Mix, 1 µg pQE-30 UA Vector DNA (50 ng/µl), distilled water	32179
QIAGEN A-Addition Kit (40)	For 40 A-addition reactions: 5x QIAGEN A-Addition Master Mix, distilled water (1.7 ml)	231994
**PCR-product cleanup kits**		
MinElute PCR Purification Kit (50)	50 MinElute Spin Columns, Buffers, Collection Tubes (2 ml)	28004
QIAquick PCR Purification Kit (50)	For purification of 50 PCR reactions: 50 QIAquick Spin Columns, Buffers, Collection Tubes (2 ml)	28104

Ordering Information

Product	Contents	Cat. No.
**Ni-NTA Matrices**		
Ni-NTA Agarose (25 ml)	25 ml nickel-charged resin (max. pressure: 2.8 psi)	30210
Ni-NTA Agarose (100 ml)	100 ml nickel-charged resin (max. pressure: 2.8 psi)	30230
Ni-NTA Agarose (500 ml)	500 ml nickel-charged resin (max. pressure: 2.8 psi)	30250
Ni-NTA Superflow (25 ml)	25 ml nickel-charged resin (max. pressure: 140 psi)	30410
Ni-NTA Superflow (100 ml)	100 ml nickel-charged resin (max. pressure: 140 psi)	30430
Ni-NTA Superflow (500 ml)	500 ml nickel-charged resin (max. pressure: 140 psi)	30450
Ni-NTA Superflow Columns (12 x 1.5 ml)	For 12 6xHis-tagged protein preps: 12 polypropylene columns containing 1.5 ml Ni-NTA Superflow	30622
Ni-NTA Superflow Columns (144 x 1.5 ml)	For 144 6xHis-tagged protein preps: 12 x 12 polypropylene columns containing 1.5 ml Ni-NTA Superflow	30624
Ni-NTA Spin Columns (50)	50 Spin Columns, Collection Tubes	31014
Ni-NTA Spin Kit (50)	50 Ni-NTA Spin Columns, Reagents, Buffers, Collection Tubes (2 ml)	31314
Polypropylene Columns (1 ml)	50/pack, 1 ml capacity	34924
Polypropylene Columns (5 ml)	50/pack, 5 ml capacity	34964
**Factor Xa System**		
Factor Xa Protease	400 units Factor Xa Protease (2U/µl)	33223
Xa Removal Resin	2 x 2.5 ml Xa Removal Resin	33213
**TAGZyme System**		
TAGZyme Kit	For exoproteolytic removal of affinity tags from up to 10 mg protein	34300

Ordering Information

Product	Contents	Cat. No.
**Anti·His Antibodies**		
RGS·His Antibody (100 µg)	100 µg mouse anti-RGS(His)$_4$ (lyophilized, with BSA, for 1000 ml working solution)	34610
RGS·His Antibody, BSA-free (100 µg)	100 µg mouse anti-RGS(His)$_4$ (lyophilized, BSA-free, for 1000 ml working solution)	34650
RGS·His Antibody, BSA-free (1 mg)	1 mg mouse anti-RGS(His)$_4$ antibody (lyophilized, BSA-free)	Inquire
Penta·His Antibody, BSA-free (100 µg)	100 µg mouse anti-(His)$_5$ (lyophilized, BSA-free, for 1000 ml working solution)	34660
Penta·His Antibody, BSA-free (1 mg)	1 mg mouse anti-(His)$_5$ antibody (lyophilized, BSA-free)	Inquire
Tetra·His Antibody, BSA-free (100 µg)	100 µg mouse anti-(His)$_4$ (lyophilized, BSA-free, for 1000 ml working solution)	34670
Tetra·His Antibody, BSA-free (1 mg)	1 mg mouse anti-(His)$_4$ antibody (lyophilized, BSA-free)	Inquire
Anti·His Antibody Selector Kit	RGS·His Antibody, Penta·His Antibody, Tetra·His Antibody, all BSA-free, 3 µg each	34698

Ordering Information

Product	Contents	Cat. No.
**Anti·His HRP Conjugates**		
RGS·His HRP Conjugate Kit	125 µl RGS·His HRP Conjugate, 5 g Blocking Reagent, 50 ml Blocking Reagent Buffer, 10x Concentrate	34450
Penta·His HRP Conjugate Kit	125 µl Penta·His HRP Conjugate, 5 g Blocking Reagent, 50 ml Blocking Reagent Buffer, 10x Concentrate	34460
Tetra·His HRP Conjugate Kit	125 µl Tetra·His HRP Conjugate, 5 g Blocking Reagent, 50 ml Blocking Reagent Buffer, 10x Concentrate	34470
**Ni-NTA Conjugates**		
Ni-NTA AP Conjugate	Alkaline-phosphatase–conjugated Ni-NTA (lyophilized, for 500 ml working solution)	34510
Ni-NTA HRP Conjugate	Horseradish-peroxidase–conjugated Ni-NTA (lyophilized, for 500 ml working solution)	34530
**Related Products**		
6xHis Protein Ladder	6xHis-tagged marker proteins (lyophilized, for 50–100 lanes on western blots)	34705
**Transfection Reagents**		
SuperFect Transfection Reagent (1.2 ml)	For 40 transfections in 60 mm dishes or 160 transfections in 12-well plates	301305
Effectene Transfection Reagent (1 ml)	1 ml Effectene Reagent, Enhancer, Buffer; for 40 transfections in 60 mm dishes or 160 transfections in 12-well plates	301425
PolyFect Transfection Reagent (1.0 ml)	For 25–65 transfections in 60 mm dishes, or 50–100 transfections in 6-well plates	301105

Ordering Information

Product	Contents	Cat. No.
**Ni-NTA HisSorb Strips and Plates**		
Ni-NTA HisSorb Strips (24)	2 racks of 12 x Ni-NTA-coated 8-well strips in 96-well format	35023
Ni-NTA HisSorb Plates (5)	5 Ni-NTA–coated, transparent 96-well plates	35061
**Ni-NTA Magnetic Bead System**		
Ni-NTA Magnetic Agarose Beads (2 x 1 ml)	2 x 1 ml nickel-charged magnetic agarose beads (5% suspension)	36111
Ni-NTA Magnetic Agarose Beads (6 x 1 ml)	6 x 1 ml nickel-charged magnetic agarose beads (5% suspension)	36113
12-Tube Magnet	Magnet for separating magnetic beads in 1.5 ml or 2 ml tubes	36912
96-Well Magnet Type A	Magnet for separating magnetic beads in wells of 96-well microplates, 2 x 96-Well Microplates FB	36915
96-Well Microplates FB (24)	96-well microplates with flat-bottom wells, 24 per case, for use with the 96-Well Magnet	36985

Trademarks

Patented or patent-pending and/or registered or registration-pending trademarks of QIAGEN: QIAGEN®, QIA*express*®, QIA*expressionist*™, QIAquick®, BioRobot®, Effectene™, HisSorb™, Ni-NTA, MinElute™, Penta·His™, PolyFect®, RGS·His™, SuperFect™, Tetra·His™.
Hoffmann-La Roche owns patents and patent applications pertaining to the application of Ni-NTA resin (Patent series: RAN 4100/63: USP 4.877.830, USP 5.047.513, EP 253 303 B1), and to 6xHis-coding vectors and His-labeled proteins (Patent series: USP 5.284.933, USP 5.310.663, EP 282 042 B1). All purification of recombinant proteins by Ni-NTA chromatography for commercial purposes, and the commercial use of proteins so purified, require a license from Hoffmann-La Roche.

ABTS is a registered trademark of Boehringer Mannheim GmbH. BaculoGold is a trademark of Becton, Dickinson and Company. BacVector is a registered trademark of Novagen, Inc. CDP-Star is a trademark of Tropix, Inc. Coomassie is a trademark of ICI Organics Inc. DAPase, pGAPase, Qcyclase, and TAGZyme are trademarks of UNIZYME. FPLC is a registered trademark of Pharmacia Biotech AB. High Five is a trademark of Invitrogen Corporation Igepal is a registered trademark of Rhone-Poulenc, Inc. Macintosh is a registered trademark of Apple Computer, Inc. Microsoft is a registered trademark of Microsoft Corporation. Superflow is a trademark of Sterogene Bioseparations Inc. Triton is a registered trademark of Union Carbide Inc. Tween is a registered trademark of ICI Americas Inc. Registered names, trademarks, etc. used in this document, even when not specifically marked as such, are not to be considered unprotected by law.

The PCR process is covered by U.S. Patents 4,683,195 and 4,683,202 and foreign equivalents owned by Hoffmann-La Roche AG.

QIAGEN Companies

Please see the inside front cover for contact information for your local QIAGEN office.

QIAGEN Distributors

Argentina
Tecnolab S.A.
Tel: (011) 4555 0010
Fax: (011) 4553 3331
E-mail: info@tecnolab.com.ar
Web site: www.tecnolab.com.ar

Austria
VWR International GmbH
Tel: (01) 576 00 0
Fax: (01) 576 00 600
E-mail: info@at.vwr.com
Web site: www.vwr.com

Belgium/Luxemburg
Westburg b.v.
Tel: 0800-1-9815
Fax: (31) 33-4951222
E-mail: info@westburg.nl
Web site: www.westburg.nl

Brazil
Uniscience do Brasil
Tel: 011 3622 2320
Fax: 011 3622 2323
E-mail: info@uniscience.com
Web site: www.uniscience.com

China
Gene Company Limited
Tel: (852)2896-6283
Fax: (852)2515-9371
E-mail:
Hong Kong: info@genehk.com
Beijing: gene@public2.bta.net.cn
Shanghai: gene@public.sta.net.cn
Chengdu: gene@public.cd.sc.cn
Guangzhou:
gzyitao@public.guangzhou.gd.cn

Croatia
INEL Medicinska Tehnika d.o.o.
Tel: (01) 2984-898
Fax: (01) 6520-966
E-mail:
inel-medicinska-tehnika@zg.tel.hr

Cyprus
Scientronics Ltd
Tel: 02-357 22 765416
Fax: 02-357 22 764614
E-mail: a.sarpetsas@biotronics.com.cy

Czech Republic
BIO-CONSULT spol. s.r.o.
Tel/Fax: (420) 2 417 29 792
E-mail: bio-cons@login.cz
Web site: www.bio-consult.cz

Denmark
VWR International A/S
Tel: 43 86 87 88
Fax: 43 86 87 90
E-mail: info@dk.vwr.com
Web site: www.vwr.com

Egypt
Clinilab
Tel: 52 57 212
Fax: 52 57 210
E-mail: Clinilab@link.net

Finland
VWR International Oy
Tel: (09) 804 551
Fax: (09) 8045 5200
E-mail: info@fi.vwr.com
Web site: www.vwr.com

Greece
BioAnalytica S.A.
Tel: (210)-640 03 18
Fax: (210)-646 27 48
E-mail: bioanalyt@hol.gr

Hungary
Kasztel-Med Co. Ltd.
Tel: (01) 385 3887
Fax: (01) 381 0695
E-mail: info@kasztel.hu
Web site: www.kasztel.hu

India
Genetix
Tel: (011)-2542 1714
or (011)-2515 9346
Fax: (011)-2546 7637
E-mail: genetix@nda.vsnl.net.in

Israel
Westburg (Israel) Ltd.
Tel: 08-6650813/4
or 1-800 20 22 20 (toll free)
Fax: 08-6650934
E-mail: info@westburg.co.il
Web site: www.westburg.co.il

Korea
LRS Laboratories, Inc.
Tel: (02) 924-86 97
Fax: (02) 924-86 96
E-mail: webmaster@lrslab.co.kr
Web site: www.lrslab.co.kr

Malaysia
RESEARCH BIOLABS SDN. BHD.
Tel: (603)-8070 3101
Fax: (603)-8070 5101
E-mail: biolabs@tm.net.my
Web site: www.researchbiolabs.com

Mexico
Quimica Valaner S.A. de C.V.
Tel: (55) 55 25 57 25
Fax: (55) 55 25 56 25
E-mail: qvalaner@infosel.net.mx

The Netherlands
Westburg b.v.
Tel: (033)-4950094
Fax: (033)-4951222
E-mail: info@westburg.nl
Web site: www.westburg.nl

New Zealand
Biolab Ltd
Tel: (09) 980 6700
or 0800 933 966
Fax: (09) 980 6788
E-mail:
biosciences@nzl.biolabgroup.com
Web site: www.biolabgroup.com/nzl

Norway
VWR International AS
Tel: 22 90 00 00
Fax: 22 90 00 40
E-mail: info@no.vwr.com
Web site: www.vwr.com

Poland
Syngen Biotech Sp.z.o.o.
Tel: (071) 351 41 06
or 0601 70 60 07
Fax: (071) 351 04 88
E-mail: info@syngen.pl
Web site: www.syngen.pl

Portugal
IZASA PORTUGAL, LDA
Tel: (21) 424 7312
Fax: (21) 417 2674

Singapore
Research Biolabs Pte Ltd
Tel: 62731066
Fax: 62734914
E-mail: biolabs@singnet.com.sg

Slovak Republic
BIO-CONSULT Slovakia spol. s.r.o.
Tel/Fax: (02) 5022 1336
E-mail: bio-cons@post.sk
Web site: www.bio-consult.cz

Slovenia
MEDILINE d.o.o.
Tel: (01) 830-80-40
Fax: (01) 830-80-70
or (01) 830-80-63
E-mail: mediline@siol.net

South Africa
Southern Cross Biotechnology
(Pty) Ltd
Tel: (021) 671 5166
Fax: (021) 671 7734
E-mail: info@scb.co.za

Spain
IZASA, S.A.
Tel: (93) 902.20.30.90
Fax: (93) 902.22.33.66
E-mail: suministros@izasa.es

Sweden
VWR International AB
Tel: (08) 621 34 00
Fax: (08) 760 45 20
E-mail: info@se.vwr.com
Web site: www.vwr.com

Taiwan
TAIGEN Bioscience Corporation
Tel: (02) 2880 2913
Fax: (02) 2880 2916
E-mail: taigen@ms10.hinet.net

Thailand
Theera Trading Co. Ltd.
Tel: (02) 412-5672
Fax: (02) 412-3244
E-mail: theetrad@samart.co.th

Turkey
Medek Medikal Ürünler
ve Saglik Hizmetleri A. S.
Tel: (216) 302 15 80
Fax: (216) 302 15 88
E-mail: makialp@med-ek.com

All other countries
QIAGEN GmbH, Germany

1024473 06/2003

QIAGEN
