# Supplementary material for: Ragweed Major Allergen Amb a 11 Recombinant Production and Clinical Implications
Source: Biomolecules. 2023 Jan 16;13(1):182. doi: 10.3390/biom13010182 (PMC9855870; doi:10.3390/biom13010182)
Supplement: Supplementary file 1 [file biomolecules-13-00182-s001.zip › biomolecules-2125843-supplementary/Table S1-Biomolecules-Tamas.pdf]

Table nr. 3. Logistic regression analysis

Dependent Y	Asthma symptoms score = 3 or more

Method	Backward
Enter variable if P<	0.05
Remove variable if P>	0.25

Sample size	150
Positive cases (symptoms score ≥3)	53 (35.33%)
Negative cases (symptoms score <3)	97 (64.67%)

Overall Model Fit

Null model -<br>2 Log Likelihood	194.846
Full model -2 Log Likelihood	167.112
Chi-squared	27.733
DF	6
Significance level	P = 0.0001
Cox & Snell R^2	0.1688
Nagelkerke R^2	0.2321

Coefficients and Standard Errors

Variable	Coefficient	Std. Error	Wald	P
IgE sensitization to Amb a 10	0.60315	0.50962	1.4007	0.2366
IgE sensitization to Amb a 11	1.57292	0.49899	9.9363	0.0016
IgE sensitization to Amb a 6	-0.67496	0.43405	2.4181	0.1199
Positive dog SPT	2.01305	0.60417	11.1016	0.0009
Positive fungi SPT	-1.03032	0.59917	2.9570	0.0855
Domestic exposure to cat	1.20677	0.48511	6.1883	0.0129
Constant	-2.06244	0.50600	16.6139	<0.0001

Variables not included in the model
Age (>40 years)
Allergy history (>6 years)
Positive HDM SPT
Positive family allergic disease history
Polysensitization status
IgE sensitization to Amb a 4
IgE sensitization to Amb a 5
IgE sensitization to Amb a 8
IgE sensitization to Amb a 9
IgE sensitization to Amb a 12
Patients sex
Professional exposure (dust, smoke, solvents)
Current or passive smoker status
Positive cat SPT
Positive gramineae SPT

Domestic exposure to dog
Domestic exposure to rodents

Odds Ratios and 95% Confidence Intervals

Variable	Odds ratio	95% CI
IgE sensitization to Amb a 10	1.8279	0.6732 to 4.9630
IgE sensitization to Amb a 11	4.8207	1.8128 to 12.8193
IgE sensitization to Amb a 6	0.5092	0.2175 to 1.1922
Positive dog SPT	7.4861	2.2907 to 24.4646
Positive fungi SPT	0.3569	0.1103 to 1.1549
Domestic exposure to cat	3.3427	1.2917 to 8.6502

Hosmer & Lemeshow test

Chi-squared	3.0063
DF	6
Significance level	P = 0.8081

Group	Y=0		Y=1		Total
	Observed	Expected	Observed	Expected	
1	15	14.092	0	0.908	15
2	12	13.220	3	1.780	15
3	17	17.931	6	5.069	23
4	11	9.558	3	4.442	14
5	27	26.660	16	16.340	43
6	8	8.514	9	8.486	17
7	5	5.346	10	9.654	15
8	2	1.680	6	6.320	8

Classification table (cut-off value p=0.5)

Actual group	Predicted group		Percent correct
	0	1	
Y = 0	86	11	88.66%
Y = 1	30	23	43.40%
Percent of cases correctly classified			72.67%

ROC curve analysis

Area under the ROC curve (AUC)	0.743
Standard Error	0.0410
95% Confidence interval	0.665 to 0.810

MedCalc® Statistical Software version 20.113 (MedCalc Software Ltd, Ostend, Belgium; https://www.medcalc.org; 2022)
